# Supplementary material for: Insights into the pathogenesis and differential diagnosis of clival lesions in an individual from a 16th-century-CE mass grave at Mohács (Southwestern Hungary)
Source: PLoS One. 2026 Jan 16;21(1):e0340762. doi: 10.1371/journal.pone.0340762 (PMC12810923; doi:10.1371/journal.pone.0340762)

S3 Figure: A possible pathogenetic scenario that could account for the development of the cranial lesions observed in MMG3-75: tuberculosis involved the meninges first (i.e., tuberculous meningitis) and then reached the clivus (i.e., tuberculous clival osteomyelitis) (image by Marcos De Andrés Montero and Olga Spekker)

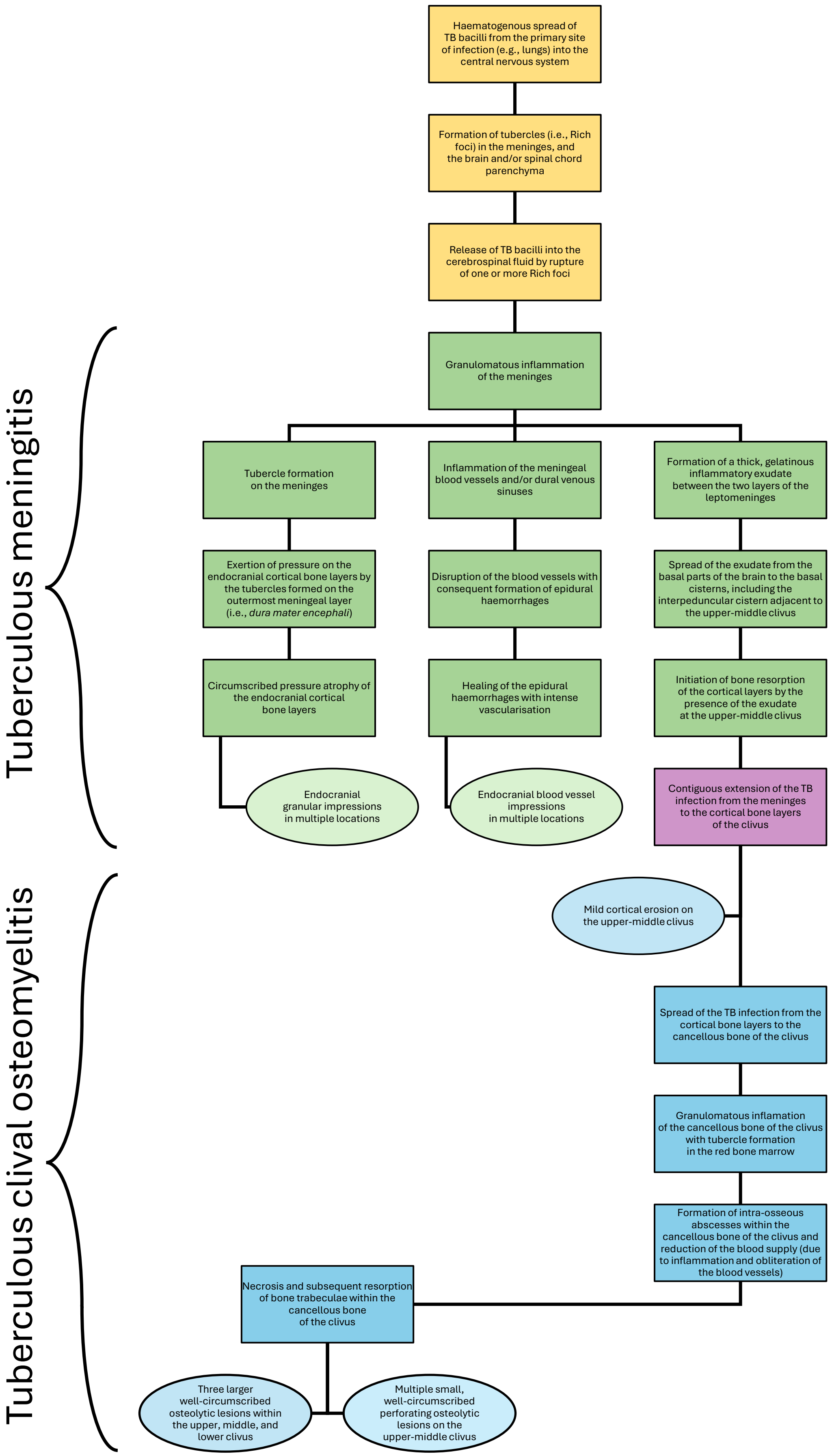

Supplement: S3 Fig — (PDF) [file pone.0340762.s003.pdf]
