# Supplementary material for: Insights into the pathogenesis and differential diagnosis of clival lesions in an individual from a 16th-century-CE mass grave at Mohács (Southwestern Hungary)
Source: PLoS One. 2026 Jan 16;21(1):e0340762. doi: 10.1371/journal.pone.0340762 (PMC12810923; doi:10.1371/journal.pone.0340762)
Supplement: S1 Text — (DOCX) [file pone.0340762.s004.docx]

**S1 Text: Detailed refutation of Genchi & colleagues’ (2025) hypothesis that endocranial granular impressions are signs of tuberculosis infection rather than clinically active tuberculosis disease.**

In the palaeopathological literature, endocranial granular impressions (GIs) were described as specific signs of tuberculous meningitis (TBM) by Schultz [1-3], Schultz & Schmidt-Schultz [4], and Spekker & colleagues [5-6]. However, in a recent paper by Genchi & co-workers [7], the authors argued against that GIs can be considered as pathognomonic features of clinically active TB(M) disease; instead, they stated that GIs should be interpreted as signs of TB infection. The authors’ argument is based on that they found a quite high frequency of cases with GIs in a mediaeval (10^th^–12^th^-century-CE) osteoarchaeological series (Pieve di Pava, Italy) [7] that seems to be in conflict with modern disease prevalence data as TBM is a very rare extra-pulmonary form, occurring in less than 1% of all clinically active TB cases today [8-10]. Genchi & colleagues [7] state that their hypothesis is supported by 1) the short duration of TBM that is not long enough to allow bony changes (e.g., GIs) to develop on the endocranial surface and 2) the presence of cases with GIs not only among TB patients but also non-TB individuals from documented skeletal collections (Hamann-Todd Human Osteological Collection [11] and Robert J. Terry Anatomical Skeletal Collection [5-6]). However, all of the authors’ [7] above arguments are refutable:

1. Genchi & co-workers [7] calculated only the frequency of cases with GIs (and based on this, the number of diagnosed cases with clinically active TBM disease/frequency of TBM), not the prevalence of TBM, in the examined osteoarchaeological series from mediaeval Pieve di Pava. Then, the authors compared the calculated frequency of cases with GIs/frequency of TBM to modern disease prevalence data and assumed that if GIs would be pathognomonic features of clinically active TB(M) disease, the prevalence of TBM would be too high in the once-lived population of Pieve di Pava (compared to a prevalence of less than 1% of all clinically active TB cases today). However, only the prevalence of TBM in mediaeval Pieve di Pava may be appropriate to be compared to modern disease prevalence data. As it has been highlighted by Milner & Boldsen [12], estimating the prevalence of a disease (e.g., TBM) in osteoarchaeological series is no easy task: “*Estimating disease prevalence from archaeological bones and teeth is no easy task (Boldsen and Milner, 2012; DeWitte and Stojanowski, 2015; Ortner, 1991; Wood et al., 1992; Wright and Yoder, 2003).*”. It is much more complicated than considering only the frequency of a lesion type (e.g., GIs) or the number of diagnosed cases (e.g., number of diagnosed cases with clinically active TB(M) disease) in the examined sample [12];
2. Only disease prevalence data deriving from a representative sample may be appropriate to be used for comparing to modern disease prevalence data but at the current state of research, it is not possible to properly estimate the prevalence of TBM in the once-lived population of Pieve di Pava.

On the one hand, the sample examined by Genchi & colleagues [7] does not seem to be representative of mediaeval Pieve di Pava. First, from the more than 900 excavated individuals, only 383 skeletons were subjected to an anthropological analysis, and only 212 were evaluated for the presence of GIs on the inner skull surface, which is less than one-fourth of the excavated individuals. Second, as Milner & Boldsen [12] argues: “*Perhaps it is best to start with a misconception: we are primarily concerned with skeletons. They are indeed what are observed, but they are most assuredly not what we wish to know about. When estimating the prevalence of a pathological condition in the past, it is the once-living people who are of concern. They are the people who would be examined, measured, and described if it were possible to be magically transported back in time. The skeletons we look at, in contrast, are from the unfortunates who failed to survive to the next age interval. They are, in effect, life’s failures each step of the way from infancy to old age.*”; “*Identifying the people at risk from whatever is of interest is critical because the population represented by skeletons has a major effect on the research questions that can be asked of the data at hand. Not just any group of skeletons can be considered to represent a population for analytical purposes. Skeletons, of course, are a sample of the population of interest, even if they are from a completely excavated cemetery. That does not necessarily present a problem because in many fields small samples are routinely used to say something useful about much larger populations. The difficulty when examining skeletal remains lies in ascertaining the representativeness of the sample, a problem commonly overlooked by tacit agreement.* *Sometimes it is even confused with the size of the sample, such as when many skeletons, without further consideration, are reflexively thought to ensure better results.*”; “*One reason it might be a good idea to avoid the word population when referring to excavated human remains pertains to the very nature of archaeological materials. Skeletons are necessarily a biased sample of the people who were once alive, hence the population from which the bones were derived. As pointed out here and elsewhere (e.g., Wood et al., 1992), what comes to a bioarchaeologist’s attention are people sampled at the moment of their death. They are assuredly not the full range of people who would be observed if one could actually survey a past living population. To once again state the obvious, one cannot avoid sampling dead people when examining skeletons. For bioarchaeological purposes, therefore, the ideal sample is one that is biased only by the fact that the individuals examined are those who were removed by death from the population at each age.*”. “*In some instances, it is possible for researchers to identify the limits of the group being examined, hence have something approximating a population. The matter is simplified when the past community associated with the cemetery was a reasonably stable group of people over time. Examples might include cemeteries associated with villages of subsistence agriculturalists or medieval peasants that tended to be spatially discrete communities, were characterized by relatively little internal socioeconomic differentiation, and were often associated with single burial areas.*”; and “*It follows that all skeletal assemblages are not equally useful for paleoepidemiological purposes. Questionable examples include skeletons from burial grounds associated with monasteries, hospitals, and shrines that attracted people from distant places (Inskip et al., 2015; Müldner et al., 2009; Roffey et al., 2017; Torino et al., 2015). Even when those institutions mostly drew from local communities, the skeletons might not be representative of the people who inhabited those places. Medieval hospitals, for example, also doubled as almshouses (Harvey and Oeppen, 2001). In such situations, poor people could be overrepresented relative to the surrounding population.*”. Based on the above, the sample examined by Genchi & co-workers [7] cannot be considered representative of the once-lived population of Pieve di Pava. For instance, the authors state in the Archaeological background section that 1) subadults are underrepresented (“*Most individuals excavated were adults, while infants and young children were largely underrepresented (Riccomi, 2021); it is likely that they were buried in a distinct funerary area that has not yet been archeologically identified (Felici, 2016).*”) and 2) the cemetery is associated with a religious building (“*The area surrounding the religious building was intensively used between the 10th and 12th centuries, as documented by the large number of west-east orientated burials (Felici, 2016). Although archeological investigations have not been able to identify the settlement associated with the religious building, several anthropological, paleopathological, and biochemical studies revealed interesting aspects of the living conditions, health, and diet of a substantial number of individuals from this site (Ricci et al., 2012; Fornaciari, 2016; Riccomi, 2021; Riccomi et al., 2021; Kinkopf et al., 2022; Monaco et al., 2022).*”). Based on the above arguments of Milner & Boldsen [12], these two aspects already make the representativeness of the examined sample from mediaeval Pieve di Pava highly questionable.

On the other hand, right now, it is impossible to properly estimate the prevalence of clinically active TB(M) disease in osteoarchaeological series as the existing palaeoepidemiological methods are not yet appropriate for that. Hopefully, in the future, it will become possible – there are already some promising studies [e.g., 13] that try to assess the prevalence of clinically active TB disease in osteoarchaeological series. Nonetheless, even if such palaeoepidemiological methods will be available in the future, the sample examined by Genchi & colleagues [7] (at least in its present form) would not be appropriate for these kind of analyses as it is not representative of the once-lived population of Pieve di Pava;

1. By comparing the calculated frequency of cases with GIs/frequency of TBM in mediaeval Pieve di Pava to modern disease prevalence data and then, stating that GIs cannot be pathognomonic features of clinically active TB(M) disease (as otherwise the prevalence of TBM would be too high in the once-lived population of Pieve di Pava compared to a prevalence of less than 1% of all clinically active TB cases today), Genchi & co-workers [7] assume that the prevalence of TB(M) in the past (e.g., mediaeval period) was no different from (or very similar to) its prevalence today. However, the prevalence of TB(M) can be influenced by numerous factors (e.g., nutritional status, age, sex, and genetic factors of the host, and/or genetic factors of the pathogen) [14-15].

Based on modern disease prevalence data, TB is a very rare disease in developed countries today [16]. If the authors’ above assumption would be correct, this would mean that TB was a very rare disease also in the past, but this is not true as we know that for instance, in the 19^th^ century and at the beginning of the 20^th^ century, the prevalence of TB disease was much higher [17-18]. We also know that for instance, after the introduction of the BCG vaccine, the prevalence of TBM substantially decreased as the BCG vaccination has a significant protective effect against the disease [19-20]. Despite this, the reported prevalence of TBM (e.g., ~7–12% of all cases with clinically active TB disease in [21]) was higher a couple of decades ago than today (e.g., ~1% of all cases with clinically active TB disease in [22]). Furthermore, recent studies [23-24] showed that because of a decrease in BCG immunization coverage or BCG vaccine supply shortages, the number of paediatric cases with TBM significantly increased in Bosnia and Herzegovina, and South Africa, respectively. Therefore, it is not correct if the authors assume that the prevalence of TBM in the past was no different from (or very similar to) its prevalence today. Currently, we do not know anything about how the prevalence of clinically active TB(M) disease has changed over time as we cannot properly estimate the prevalence of TB(M) in past human populations (e.g., mediaeval Pieve di Pava). Nevertheless, it has been reported in some palaeopathological papers [e.g., 25-27] that before the introduction of the BCG vaccine and anti-TB chemotherapy with antibiotics, the prevalence of TB and its forms (e.g., TBM), and the frequency of some TB-associated bony changes were different from today’s trends.

Finally, our understanding of the global burden of TBM today is limited but it is likely that numerous cases are not diagnosed; thus, the prevalence of TBM, especially in high-TB-burden countries, may be much higher than previously thought [28-29];

1. By questioning the possibility of the development of endocranial bony changes (e.g., GIs) in TBM patients, Genchi & colleagues [7] also assume that the natural history and duration of TB(M) disease in the past (e.g., mediaeval period) were no different from (or very similar to) its natural history and duration today. However, like the prevalence of TB(M), these aspects of the disease can be influenced by numerous factors [14-15].

As it has been highlighted by Spekker & co-workers [5] or Madai & colleagues (see Supplementary File 2 in [30]), in the modern medical literature, there are reports of cases (some come from the pre-antibiotic era, some from after the introduction of anti-TB chemotherapy with antibiotics) in which TBM had a protracted course (several months or even years) [e.g., 31-37]. At least in such cases (the prevalence of which is not known neither today nor in the past (e.g., mediaeval period)), the duration of the disease is long enough to allow skeletal lesions (e.g., GIs) to develop on the inner skull surface.

Furthermore, as it has been highlighted by Schultz [1-3] or Spekker & co-workers [5-6,38], GIs are the result of pressure atrophy – they match in their position to the tubercles of the *dura mater encephali* that can cause bone resorption (atrophy) by exploring pressure on the adjacent endocranial surface. For instance, in Schultz [1]: “*However, there are also specific changes, which are only caused by a tuberculous process of the meninges. These characteristic lesions are small, roundish, and relatively flat impressions in the endocranial lamina of the skull and are caused by pressure atrophy of the tuberculars.*”. Or in Spekker & colleagues [5]: “*According to the results of Templin [28], Templin & Schultz [29], Schultz [24–26], and Schultz & Schmidt-Schultz [27], GIs may be established by pressure atrophy of the tubercles formed in the dura mater during later stages of TBM [24–29].*”. Or in Spekker & co-workers [38]: “*In later stages of the disease, not only the leptomeninges but also the outermost meningeal layer (i.e., the dura mater encephali) could have become affected by the pathological process [66, 84, 85]. As the dura mater encephali is directly adherent to the inner surface of the cranium, the gradually growing and coalescing meningeal tubercles could have exerted localised pressure on the underlying bone, inducing temporary, circumscribed bone atrophy and subsequent formation of endocranial GIs (Figs 2, 4, 5, 6A, 6B, 6D–6E, 15 and 17) [62, 65, 66, 69]. By definition, GIs are small (0.5–1.0 mm in diameter), relatively shallow (less than 0.5 mm in depth), roundish impressions with smooth margins and walls [62–66, 69]. They can appear separately or confluently and are usually grouped in clusters on the inner surface of the cranium [62–66, 69]. Granular impressions match in their position to those tubercles of the dura mater encephali, which cause bone resorption by exploring pressure on the underlying bone [62–66, 69].*”.

Similar to GIs, abnormally pronounced digital impressions (APDIs) are the result of pressure atrophy – they match in their position to the cerebral gyri that can cause bone resorption (atrophy) by exploring pressure on the adjacent endocranial surface. In case of APDIs, this pathological process (i.e., the formation of APDIs by pressure atrophy) requires several weeks. For instance, in Spekker & colleagues [39]: “*In adulthood, the presence of APDIs over the upper portion of the skull indicates a prolonged rise in the intracranial pressure: according to estimates, the formation of APDIs secondary to elevated intracranial pressure (eICP) requires at least ten weeks [29,30,32–36].*”.

Based on the above, we can conclude that the formation of GIs by pressure atrophy (which are much smaller lesions than APDIs) can require several weeks, as well. Thus, even in cases with TBM where there is no initiation of anti-TB chemotherapy with antibiotics and the disease do not have a protracted course but a typical one, the duration of TBM can be long enough to allow skeletal lesions (e.g., GIs or APDIs) to develop, especially in consideration that the period between the onset of the signs and symptoms of the disease and the death of the patient, which is preceded by the onset of the formation of tubercles on the meninges, is already several weeks or months (usually two to eight weeks) [40-41];

1. By stating that the presence of cases with GIs not only among TB patients but also non-TB individuals from documented skeletal collections (Hamann-Todd Human Osteological Collection [11] and Robert J. Terry Anatomical Skeletal Collection [5-6]) supports that GIs are signs of TB infection rather than clinically active TB(M) disease, Genchi & co-workers [7] seem to overlook that in the two aforementioned documented skeletal collections, individuals were divided into two groups based on what they died of – TB group (those who died of TB) and non-TB group (those who died of non-TB causes) – as there is information about their recorded cause of death only. We do not know the complete medical history of these individuals; thus, even if some of them died of a non-TB cause (e.g., accident, cancer or cardiovascular disease), it does not mean that they did not suffer from clinically active TB(M) disease (with presenting signs and symptoms) at the time of death or that they were not diagnosed with TB(M) disease before they died. The only thing we know is what they died of – TB or a non-TB cause. Based on the above, we do not know if someone who died of a non-TB cause; and thus, was placed into the non-TB groups of the above-mentioned two documented skeletal collections had clinically active TB(M) disease or not (i.e., was a TB(M) patient or not) at the time of death. Consequently, in the non-TB groups of the Hamann-Todd Human Osteological Collection and the Robert J. Terry Anatomical Skeletal Collection, there may be at least a few TB(M) patients [11,13] and this is why it is not surprising that some of the individuals who died of non-TB causes presented GIs, indicating that they suffered from clinically active TB(M) disease (even though TB(M) was not their recorded cause of death).

Not to mention that there can be cases in which an inaccurate cause of death was recorded. As it has been highlighted by Spekker & colleagues [5] regarding the Robert J. Terry Anatomical Skeletal Collection: “*It must be noted that even if the recorded cause of death of individuals surveyed in the Terry Collection may not have been TB, individuals could still have suffered from the disease but their death was attributed to another medical condition [18–19]. Moreover, there is always the possibility that an inaccurate cause of death was registered on the morgue record and/or death certificate of individuals from the Terry Collection. Thus, it is possible that in the aforementioned six cases, the observed endocranial and non-endocranial bony changes resulted from TB. In summary, the findings of our study confirm those of Schultz [e.g., 24–26] and Schultz & Schmidt-Schultz [27] that GIs can be considered as pathognomonic features of TBM; and therefore, the paleopathological diagnosis of TBM can be established with a high certainty when GIs are present in ancient human bone remains. The localization pattern and distribution of GIs on the endocranial surface resemble that of the tubercles observed in the affected meninges during the pathogenesis of TBM that further strengthens their tuberculous origin*.”. Santos [42] has also mentioned regarding another pre-antibiotic-era documented skeletal collection, the Coimbra Identified Skeletal Collection (individuals of the collection died during the first half of the 20^th^ century CE), that “*Medical knowledge at that time suggests many cases of tuberculosis were misdiagnosed and diseases diagnosed as enteritis in children, typhoid in children and teenagers, pertussis, influenza, meningitis and pulmonary diseases, such as pleurisy and pneumonia (Carvalho, 1907), and also bronchitis (Carvalho, 1907; Porto, 1927) and emphysema, very frequent in elderly people (Porto, 1927; Lopo de Carvalho, 1935), were in fact tuberculosis. Moreover, tuberculosis in different organs of the body, such as liver, kidneys, suprarenal glands and genitals, among others, was very often not recognised (Carvalho, 1907).*”, which further strengthens the above argument that some of the individuals who died of a non-TB cause; and thus, were placed into the non-TB groups of the aforementioned documented skeletal collections, could have suffered from clinically active TB(M) disease (i.e., could have been TB(M) patients) at the time of death (even though TB(M) was not their recorded cause of death).

Furthermore, as it has been presented by Spekker & co-workers [5], in the Robert J. Terry Anatomical Skeletal Collection, from the 193 individuals in the non-TB group, only six showed GIs (6/193; ~3%). Based on the above, there is a possibility that an inaccurate cause of death was recorded in at least some of these six cases but even if the recorded cause of death is accurate, these individuals are very likely to have been TB patients who died of non-TB causes. In the same paper by Spekker & colleagues [5], the S2 Table (“*Basic biographic data of individuals in the NTB group (N=193)*”) contains detailed information (e.g., age at death, sex, and cause of death based on the morgue record (MR) and/or death certificate (DC)) about the individuals in the non-TB group. Based on these data, the recorded cause of death of the aforementioned six individuals were the following: 1) Terry No. 4R died of antral carcinoma (MR)/metastasis of the left orbit (DC); 2) Terry No. 197R died of breast carcinoma (MR); 3) Terry No. 272 died of cardiac decompensation, nephritis (MR); 4) Terry No. 465 died of post-operative wound of the chest (MR)/aortic aneurysm (DC); 5) Terry No. 506 died of myocarditis (MR)/asthma, myocarditis (DC); and 6) Terry No. 1378 died of peritonitis (MR). As it can be seen, none of these non-TB causes of death can be responsible for the development of GIs on the inner skull surface, which also supports that the aforementioned six individuals were very likely to have suffered from TB(M) (i.e., were TB patients) but died of a non-TB cause (e.g., cancer or peritonitis) that was concurrently present with TB;

1. The Hamann-Todd Human Osteological Collection and the Robert J. Terry Anatomical Skeletal Collection were established when and where many people, possibly everyone, were exposed to TB bacilli [11,13,43]; thus, individuals from the above-mentioned documented skeletal collections are likely to had TB infection – in the TB group, they were not only infected by TB but the infection progressed into clinically active TB disease in all cases, whereas in the non-TB group, they had at least latent TB infection that may have progressed into clinically active TB disease in an unknown number of cases. As it has been highlighted by Hershkovitz & co-workers [11] regarding the Hamann-Todd Human Osteological Collection: “*The presence of SES only among modern adults is explained by the nature of the HTH sample, i.e., comprised of black and white men and women born between the years 1825–1910 (prior to the initiation and widespread application of antimicrobial therapy). Moreover, all were of low socioeconomic status, living in a highly polluted urban industrial area. Of the 3,100 documented subjects in the collection, 570 had reportedly died of TB. The true number is probably much higher.*”. Or by Dangvard Pedersen & colleagues [13] regarding the Robert J. Terry Anatomical Skeletal Collection: “*Considering when and where these people lived, many of those who were not listed as sufferers of TB, and possibly all of them, were exposed to M. tuberculosis complex bacteria at some point in their lives. That is because TB was widespread in 18th to early-20th century North America, Europe, and Africa (Bates and Stead, 1993; Daniel, 2009; Madsen et al., 1942). It is likely that TB was not noted in the sparse documentation available on some individuals who had the disease with its accompanying skeletal lesions. A more immediate or obvious cause of death could have been reported, TB might not have been mentioned because of social stigma associated with acknowledging and reporting it, or the disease was not identified because diagnostic procedures were inexact and not always thorough.*”.

Based on the above, if Genchi & co-workers’ [7] hypothesis would be correct and GIs would be signs of TB infection rather than clinically active TB(M) disease, the frequency of cases with GIs in the non-TB groups should be very similar to their frequency in the TB groups (as in the non-TB groups, the majority of the individuals (if not all of them) are likely to had at least latent TB infection, whereas in the TB groups, all individuals are unquestionably to had not only TB infection but clinically active TB disease). However, as it has been presented by Spekker & colleagues [5], in the Robert J. Terry Anatomical Skeletal Collection, the frequency of cases with GIs was about 10 times higher in the TB group than in the non-TB group. This difference between the two groups was statistically extremely significant (“*The χ^2^ testing of the frequencies of GIs in individuals with TB as the cause of death and individuals with NTB causes of death revealed a statistically extremely significant difference between the two groups (χ^2^ = 47.922, df = 1, P<0.0001).*”) [5], which confirms that GIs are signs of clinically active TB(M) disease rather than TB infection;

1. If Genchi & co-workers’ [7] hypothesis would be correct and it would be the meningeal tubercles developing at the beginning of the TB infection rather than the meningeal tubercles forming during the pathogenesis of TBM that exert pressure on the adjacent endocranial surface for a long time (not weeks but more likely years or decades); and thus, lead to the development of GIs, the frequency of cases with GIs should be much higher not only in the non-TB groups of the Hamann-Todd Human Osteological Collection and Robert J. Terry Anatomical Skeletal Collection, where the majority of the individuals (if not all of them) are likely to had at least latent TB infection, but also in the TB groups, where all individuals are unquestionably to had not only TB infection but clinically active TB disease. However, as it has been presented by Spekker & colleagues [5], even in the TB group of the Robert J. Terry Anatomical Skeletal Collection, where everyone was unquestionably infected by TB, GIs were present in less than one-third of the examined cases (68/234; ~29%); and
2. The localisation pattern of GIs on the endocranial surface is not random as we whould expect it if Genchi & co-workers’ [7] hypothesis would be correct and it would be the meningeal tubercles forming at the beginning of the TB infection after haematogenous dissemination of TB bacilli into the central nervous system that exert pressure on the inner skull surface for a long time; and thus, result in the development of GIs. As it has been presented by Schultz [1], Schultz & Schmidt-Schultz [4], and Spekker & colleagues [5], instead of being randomly scattered all over the endocranial surface, GIs are localised on the skull base and lower lateral skull vault.

During the pathogenesis of TBM, the formation of meningeal tubercles presents a very specific pattern (first the disease involves the meninges of the basal areas but in the end, it extends to the meninges covering the cerebral convexities). The localisation pattern of GIs on the endocranial surface follows this specific pattern of tubercle development during the pathogenesis of TBM (see Figure 1 in [5]): GIs are most frequently located on the endocranial surface of the squamous part of the occipital bone (adjacent to the cerebellum), the squamous part of the temporal bone (adjacent to the respective temporal lobe), and the orbital parts of the frontal bone (adjacent to the respective frontal lobes). Sometimes, the greater wings of the sphenoid bone (adjacent to the respective temporal lobes) and the parietal bones (close to the squamous suture) can also exhibit GIs. As it has been highlighted by Spekker & co-workers [5]: “*The localization pattern and distribution of GIs on the endocranial surface resemble that of the tubercles observed in the affected meninges during the pathogenesis of TBM that further strengthens their tuberculous origin.*”.

In summary, the currently available scientific evidence is not in favour of Genchi & colleagues’ [7] hypothesis that GIs are signs of TB infection (rather than clinically active TB(M) disease) but supports the previous hypothesis [1-6] that GIs are pathognomonic features of TB(M) (rather than TB infection).

**REFERENCES**

1. Schultz, M. The role of tuberculosis in infancy and childhood in prehistoric and historic populations in *Tuberculosis: Past and Present* (eds Pálfi, G., Dutour, O., Deák, J. & Hutás, I.) 503-507 (TB Foundation & Golden Book Publisher, 1999).
2. Schultz, M. Paleohistopathology of bone: A new approach to the study of ancient diseases. *Am. J. Phys. Anthropol*. **116**(Suppl. 33), 106-147; [10.1002/ajpa.10024](https://doi.org/10.1002/ajpa.10024) (2001).
3. Schultz, M. Light microscopic analysis in skeletal paleopathology in *Identification of pathological conditions in human skeletal remains* (ed Ortner, D. J.) 73-107; [10.1016/B978-012528628-2/50043-0](https://doi.org/10.1016/B978-012528628-2/50043-0) (Academic Press, 2003).
4. Schultz, M. & Schmidt-Schultz, T. H. Is it possible to diagnose TB in ancient bone using microscopy?. *Tuberculosis* **95**, S80-S86; [10.1016/j.tube.2015.02.035](https://doi.org/10.1016/j.tube.2015.02.035) (2015).
5. Spekker, O. et al. Tracking down the White Plague: The skeletal evidence of tuberculous meningitis in the Robert J. Terry Anatomical Skeletal Collection. *PLOS ONE* **15**, e0230418; [10.1371/journal.pone.0230418](https://doi.org/10.1371/journal.pone.0230418) (2020).
6. Spekker, O., Hunt, D. R., Berthon, W., Molnár, E. & Pálfi, G. Insights into the diagnostic efficacy and macroscopic appearance of endocranial bony changes indicative of tuberculous meningitis: Three example cases from the Robert J. Terry Anatomical Skeletal Collection. *Int. J. Osteoarchaeol*. **32**, 444-456; [10.1002/oa.3079](https://doi.org/10.1002/oa.3079) (2022).
7. Genchi, M. L., Giuffra, V., Campana, S. & Riccomi, G. Are endocranial granular impressions pathognomonic of tuberculous meningitis or a marker of tuberculous infection? An investigation on a medieval osteoarcheological assemblage from Italy. *Int. J. Paleopathol*. **49**, 81-92; [10.1016/j.ijpp.2025.03.002](https://doi.org/10.1016/j.ijpp.2025.03.002) (2025).
8. Gauba, C. & Varma, M. Tuberculosis of the central nervous system. *Apollo Med*. **2**, 21-28; [10.1016/S0976-0016(12)60056-8](https://doi.org/10.1016/S0976-0016(12)60056-8) (2005).
9. Kim, J. et al. Procalcitonin as a diagnostic and prognostic factor for tuberculosis meningitis. *J. Clin. Neurol*. **12**, 332-339; [10.3988/jcn.2016.12.3.332](https://doi.org/10.3988/jcn.2016.12.3.332) (2016).
10. Seid, G., Alemu, A., Dagne, B. & Gamtesa, D. F. Microbiological diagnosis and mortality of tuberculosis meningitis: Systematic review and meta-analysis. *PLOS ONE* **18**, e0279203; [10.1371/journal.pone.0279203](https://doi.org/10.1371/journal.pone.0279203) (2023).
11. Hershkovitz, I. et al. *Serpens endocrania symmetrica* (SES): A new term and a possible clue for identifying intrathoracic disease in skeletal populations. *Am. J. Phys. Anthropol*. **118**, 201-216; [10.1002/ajpa.10077](https://doi.org/10.1002/ajpa.10077) (2002).
12. Milner, G. R. & Boldsen, J. L. Life not death: Epidemiology from skeletons. Int. J. Paleopathol. **17**, 26-39; [10.1016/j.ijpp.2017.03.007](https://doi.org/10.1016/j.ijpp.2017.03.007) (2017).
13. Dangvard Pedersen, D., Milner, G. R., Kolmos, H. J. & Boldsen, J. L. The association between skeletal lesions and tuberculosis diagnosis using a probabilistic approach. *Int. J. Paleopathol*. **27**, 88-100; [10.1016/j.ijpp.2019.01.001](https://doi.org/10.1016/j.ijpp.2019.01.001) (2019).
14. Maciel, E. L. & Reis-Santos, B. Determinants of tuberculosis in Brazil: From conceptual framework to practical application. *Rev. Panam. Salud. Publica*. **38**, 28-34 (2015).
15. Gowrishankar, N. C. Tuberculosis: Natural history. *Pediatr. Inf. Dis*. **1**, 4-6; [10.5005/jp-journals-10081-1102](https://doi.org/10.5005/jp-journals-10081-1102) (2019).
16. World Health Organization (WHO). *Global tuberculosis report 2024* (WHO, 2024).
17. Carthy, A. F. The treatment of tuberculosis in Ireland from the 1890s to the 1970s. A case study of medical care in Leicester. PhD Thesis (Department of History, National University of Ireland, 2015).
18. Hakosalo H. Lust for life: Coping with tuberculosis in late nineteenth-century Europe. *Med. Hist*. **64**, 516-532; [10.1017/mdh.2020.43](https://doi.org/10.1017/mdh.2020.43) (2020).
19. Walker, V., Selby, G. & Wacogne, I. Does neonatal BCG vaccination protect against tuberculous meningitis?. *Arch. Dis. Child*. **91**, 789-791; [10.1136/adc.2006.098459](https://doi.org/10.1136/adc.2006.098459) (2006).
20. Pereira, S. M., Dantas, O. M. S., Ximenes, R. & Barreto, M. L. BCG vaccine against tuberculosis: Its protective effect and vaccination policies. *Rev. Saúde. Pública*. **41**(Suppl. 1), 59-66. [10.1590/s0034-89102007000800009](https://doi.org/10.1590/s0034-89102007000800009) (2007).
21. Thilothammal, N., Krishnamurthy, P. V., Runyan, D. K. & Banu, K. Does BCG vaccine prevent tuberculous meningitis? *Arch. Dis. Child*. **74**, 144-147. [10.1136/adc.74.2.144](https://doi.org/10.1136/adc.74.2.144) (1996).
22. Dodd, P. J. et al. The global burden of tuberculous meningitis in adults: A modelling study. *PLOS Glob. Public Health* **1**, e0000069; [10.1371/journal.pgph.0000069](https://doi.org/10.1371/journal.pgph.0000069) (2021).
23. Puvacic, S., Dizdarevic, J., Santic, Z. & Mulaomerovic, M. Protective effect of neonatal BCG vaccines against tuberculous meningitis. *Bosn. J. Basic. Med. Sci*. **4**, 46-49; [10.17305/bjbms.2004.3460](https://doi.org/10.17305/bjbms.2004.3460) (2004).
24. du Preez, K. et al. Global shortages of BCG vaccine and tuberculous meningitis in children. *The Lancet Global Health* **7**, e28-e29; [10.1016/S2214-109X(18)30474-1](https://doi.org/10.1016/S2214-109X(18)30474-1) (2019).
25. Jankauskas, R. Tuberculosis in Lithuania: Paleopathological and historical correlations in *Tuberculosis: Past and Present* (eds Pálfi, G., Dutour, O., Deák, J. & Hutás, I.) 551-558 (TB Foundation & Golden Book Publisher, 1999).
26. Holloway, K. L., Link, K., Rühli, F. & Henneberg, M. Skeletal lesions in human tuberculosis may sometimes heal: An aid to palaeopathological diagnoses. *PLOS ONE* **8**, e62798; [10.1371/journal.pone.0062798](https://doi.org/10.1371/journal.pone.0062798) (2013).
27. Santos, A. L. Archives and skeletons: An interdisciplinary approach to the study of paleopathology of tuberculosis. *Tuberculosis* **95**, S109-S111; [10.1016/j.tube.2015.02.014](https://doi.org/10.1016/j.tube.2015.02.014) (2015).
28. Seddon, J. A., Solomons, R., Tugume, L., Prasad, K. & Bahr, N. C. The current global situation for tuberculous meningitis: Epidemiology, diagnostics, treatment and outcomes. *Wellcome Open Res*. **4**, 167; [10.12688/wellcomeopenres.15535.1](https://doi.org/10.12688/wellcomeopenres.15535.1) (2019).
29. Abdella, A. et al. Tuberculous meningitis in children: Treatment outcomes at discharge and its associated factors in Eastern Ethiopia: A five years retrospective study. *Infect. Drug Resist*. **15**, 2743-2751; [10.2147/IDR.S365753](https://doi.org/10.2147/IDR.S365753) (2022).
30. Madai, Á. et al. Chasing the “White Plague” in the Barbaricum of the Carpathian Basin – A case with tuberculous meningitis discovered in a Sarmatian-period (2^nd^–3^rd^-century-CE) storage pit from the archaeological site of Kiskundorozsma–Daruhalom-dűlő II (Hungary). *Tuberculosis* **152**, 102632; [10.1016/j.tube.2025.102632](https://doi.org/10.1016/j.tube.2025.102632) (2025).
31. Griffith, J. P. C. *The diseases of infants and children* (W. B. Saunders Company, 1919).
32. Schmidt, B. Chronische leptomeningitis cerebrospinalis tuberculosa. Lung 96, 124-138; [10.1007/BF02144352](https://doi.org/10.1007/BF02144352) (1941).
33. Green, J. R. Chronic tuberculous meningo-encephalitis. *J. Neuropathol. Exp. Neurol*. **2**, 148-163; [10.1097/00005072-194304000-00005](https://doi.org/10.1097/00005072-194304000-00005) (1943).
34. Saito H. Pathogenesis of tuberculous meningitis. *Acta Tuberc. Jpn*. **6**, 20-36 (1956).
35. Traub, M., Leake, J., Scholtz, C. & Thakkar, C. Chronic untreated tuberculous meningitis. *J. Neurol*. **233**, 254-256; [10.1007/BF00314030](https://doi.org/10.1007/BF00314030) (1986).
36. Kent, S. J., Crowe, S. M., Yung, A., Lucas, C. R. & Mijch, A. M. Tuberculous meningitis: A 30-year review. *Clin. Infect. Dis*. **17**, 987-994; [10.1093/clinids/17.6.987](https://doi.org/10.1093/clinids/17.6.987) (1993).
37. Newton, R. W. Tuberculous meningitis. *Arch. Dis. Child*. **70**, 364-366; [10.1136/adc.70.5.364](https://doi.org/10.1136/adc.70.5.364) (1994).
38. Spekker, O. et al. White Plague among the “forgotten people” from the Barbaricum of the Carpathian Basin–Cases with tuberculosis from the Sarmatian-period (3^rd^–4^th^ centuries CE) archaeological site of Hódmezővásárhely–Kenyere-ér, Bereczki-tanya (Hungary). *PLOS ONE* **19**, e0294762; [10.1371/journal.pone.0294762](https://doi.org/10.1371/journal.pone.0294762) (2024).
39. Spekker, O. et al. Tracking down the White Plague. Chapter three: Revision of endocranial abnormally pronounced digital impressions as paleopathological diagnostic criteria for tuberculous meningitis. *PLOS ONE* **16**, e0249020; [10.1371/journal.pone.0249020](https://doi.org/10.1371/journal.pone.0249020) (2021).
40. Brouwer, M. C. & van de Beek, D. 19 – Acute and chronic meningitis. *Infect. Dis.* **1**, 177-188.e2; [10.1016/B978-0-7020-6285-8.00019-8](https://doi.org/10.1016/B978-0-7020-6285-8.00019-8) (2017).
41. Lin, E. L.-Y., Gulhane, S. A., Kumar, M. S., Lakkamaneni, S. T. & Lekkala, P. A case report of tuberculous meningitis resulting in irreversible visual impairment due to delayed diagnosis. *Clin. Case Rep*. **12**, e9334; [10.1002/ccr3.9334](https://doi.org/10.1002/ccr3.9334) (2024).
42. Santos, A. L. TB files: New hospital data (1910–1936) on the Coimbra Identified Skeletal Collection. in *Tuberculosis: Past and Present* (eds Pálfi, G., Dutour, O., Deák, J. & Hutás, I.) 127-134 (TB Foundation & Golden Book Publisher, 1999).
43. Hunt, D. R. & Albanese, J. History and demographic composition of the Robert J. Terry anatomical collection. *Am. J. Phys. Anthropol*. **127**, 406-417; [10.1002/ajpa.20135](https://doi.org/10.1002/ajpa.20135) (2005).
