# Supplementary material for: Insights into the pathogenesis and differential diagnosis of clival lesions in an individual from a 16th-century-CE mass grave at Mohács (Southwestern Hungary)
Source: PLoS One. 2026 Jan 16;21(1):e0340762. doi: 10.1371/journal.pone.0340762 (PMC12810923; doi:10.1371/journal.pone.0340762)
Supplement: Attachment — Submitted filename: response_to_reviewers.doc [file pone.0340762.s006.doc]

Dr. Mark Spigelman, MD, PhD

Academic Editor

PLOS ONE

14 November 2025

Dear Dr. Spigelman,

We are very thankful for your and the reviewers’ insightful and constructive comments regarding our manuscript entitled “*Insights into the pathogenesis and differential diagnosis of clival lesions in an individual from a 16^th^-century-CE mass grave at Mohács (southwestern Hungary)*” that was submitted to PLOS ONE (manuscript ID: PONE-D-25-40158). We are sure that you and the reviewers helped us to improve the quality of our manuscript. The main text and a figure file have been modified following the suggestions, and a new figure and two supplementary figures have been created and added. The revised and new files have been uploaded to the submission site of PLOS ONE.

**Responses to the Academic Editor’s comments:**

1) “*Your paper is well written and quite comprehensive and together with your excellent and comprehensive bibliography I would like to have it accepted for publication.
   I have had 4 reviewers comment on its contents with unusually conflicting results. The paper is worthy of publication but it does require significant changes/alterations.*”.

Thank you very much for your kind and encouraging words regarding our manuscript. We are truly grateful for your positive evaluation of our work and for highlighting the comprehensiveness of both the manuscript and its bibliography.

We also appreciate the reviewers’ thoughtful feedback. While we recognise that some of the comments reflect differing perspectives, we fully understand that significant revisions are necessary to meet the standards required for publication. During the revision process, we have been committed to carefully addressing all the points raised and to improving the manuscript accordingly.

2) “*Reviewer 1: Is harsh in rejecting your paper but has alerted us to some significant problems but I believe if you approach the comments and provide answers to her criticism, the paper can still warrant publication. Reviewer 2: Agrees to publication but rises a number of areas which you should address. Reviewer 3: Similarly asks for some significant corrections. Reviewer 4: notes only a small problem with the anatomical description of the skeleton that should be easily corrected. I would ask you to consider each of the reviewers' views and specifically consider the title to be more general such as "A differential diagnosis of clival osteomyelitis etc etc" or something similar.*”.

We have carefully considered all four reviewers’ comments and suggestions, and have done our best to address every concern raised. In the revised version of our manuscript, we have provided detailed responses to each point and made the corresponding changes to the text.

Following your suggestion, we have also revised the title of our manuscript (“*The first archaeological case of tuberculous clival osteomyelitis with associated meningitis from a 16^th^-century-CE mass grave from Mohács (southwestern Hungary)*”) to be more general. The new title of our manuscript is: “*Insights into the pathogenesis and differential diagnosis of clival lesions in an individual from a 16^th^-century-CE mass grave at Mohács (southwestern Hungary)*”.

We hope that these revisions adequately address the concerns raised and improve the quality of our manuscript.

3) “*Further the suggestion be more specific about which piece of bone was used for DNA analysis and if it was located close or a part of the actual lesion? Same for the tooth. This is important, as if he were suffering from active TB and meningitis, it is unlikely he could have been involved in battle as a soldier*.”
a) Thank you very much for this important and insightful comment. In the revised version of our manuscript (lines 235–237), we have clarified which specific skeletal elements were used for the aDNA analysis in the *Palaeogenetic analysis* subsection of the Methods section (we sampled the upper right lateral incisor and a small fragment of the left parietal bone):

“*Hard tissue samples (the upper right lateral incisor and a fragment of the left parietal bone) of MMG3-75 were subjected to DNA-based analysis as described by Jäger & colleagues [118].*”.

Importantly, the skull sample was not taken from the area of the clival lesions or their immediate surroundings. This was a deliberate decision as we aimed to avoid damaging the pathological bony changes through invasive sampling. Our intention was to preserve the lesions, considering their importance for potential future investigations.

As a comparative example, we would like to refer to a previously studied case of leprosy (SG38) from our skeletal collection, in which a sampling for aDNA and lipid biomarker analyses was conducted directly from the rhinomaxillary region exhibiting pathological (leprosy-related) lesions. Unfortunately, the bony changes were completely destroyed during this previous sampling process, rendering any subsequent re-examination impossible (Tihanyi & colleagues, 2024). We sought to avoid a similar outcome in the case presented in our current manuscript (MMG3-75).

b) We would also like to note that, although the five known burial pits at the Mohács National Memorial Site, including mass grave No. 3, are associated with the aftermath of the Battle of Mohács (1526), at this stage of research, the identities of the individuals buried in these pits are unknown and any interpretation must be considered hypothetical. Over time, three main hypotheses have emerged (Papp, 1961; K. Zoffmann, 1982; Bertók & co-workers, 2022, 2023; Varga, 2023; Drusza, 2024; Viczián & Szeberényi, 2024):
(1) The first hypothesis proposes that the individuals buried in mass grave No. 3 – and, more generally, those interred in the five known mass graves at the Mohács National Memorial Site – were soldiers who fought in the main engagement of the Battle of Mohács and were killed either during the combat itself or while attempting to retreat. This theory has already been questioned. Although the exact location of the battle is currently unknown, all locations proposed to date lie several hundred metres, or even several kilometres, away from the five known mass graves, raising the question of why the Ottoman forces in charge of the burial would have transported the bodies of fallen Hungarian soldiers themselves, or arranged for them to be transported by others, over such a long distance. Nevertheless, the hypothesis cannot be entirely dismissed, as research into the precise location of the combat zone is still ongoing;
(2) The second hypothesis posits that these individuals were members of the Hungarian camp – soldiers guarding it and/or civilians (such as support personnel serving there and/or family members of soldiers) – who may have been killed when the camp was overrun by the Ottoman forces during the battle. This interpretation is supported by recent findings indicating that the location of the five known mass graves can be associated with one of the Hungarian camps established prior to the battle. It is possible that the burial pits were originally defensive ditches surrounding the camp, which were subsequently used by the Ottomans for the interment of the enemy dead; and
(3) The third hypothesis assumes that these individuals were captives (soldiers and/or civilians taken not only during the battle itself, but also in raids preceding or following it), who were systematically executed after the Battle of Mohács; the executions likely took place in the vicinity of the overrun Hungarian camp. This interpretation is supported by both Christian and Ottoman contemporary sources, which mention that Sultan Süleyman did not permit captives to be retained, and that during the victory divan held a few days after the battle (31 August 1526), he ordered the execution of 1,500–2,000 of them. Recent findings estimating that between 1,500–1,700 individuals were buried in the five known mass graves at the Mohács National Memorial Site correspond well with the numbers of executed captives reported in these written sources, thereby strengthening this hypothesis.

It is also possible that those interred in these five burial pits do not represent a single, homogeneous group, but rather a mixture of people executed after the battle, individuals killed when the Hungarian camp was overrun, and others who perished nearby during the fighting or retreat. In other words, the composition of the five known mass graves may not reflect an “either-or” scenario, but rather a combination of these different circumstances.

The taphonomic processes that have taken place over the 500 years since the burial of the individuals have resulted in a variably, but overall poorly preserved and partially commingled state of the more than 300 skeletons interred in mass grave No. 3. This, together with the absence of associated grave goods, greatly hinders efforts to determine the identity of these individuals and the circumstances of their deaths (a situation that also applies to the other four known mass graves at the Mohács National Memorial Site). Observations on other skeletons from mass grave No. 3 – such as evidence of advanced-stage syphilis in at least one individual, signs of tuberculous meningitis in several individuals, and the presence of skeletal elements of adolescents (12–14 years of age based on unfused epiphyses) and possible adult female(s) – further raise the question of whether all individuals interred here were directly involved in the Battle of Mohács.

Consequently, at the current stage of research, none of the aforementioned hypotheses regarding the identity of the individuals buried in mass grave No. 3 (or in the other four known mass graves at the Mohács National Memorial Site) or the circumstances of their deaths can be definitively confirmed or ruled out, and other, less-explored possibilities – such as these individuals being inhabitants of nearby settlements who were killed while assisting retreating Hungarian troops or during Ottoman raids – also remain plausible.

Based on the above, a multitude of possibilities must be considered, and the hypothesis that MMG3-75 was directly involved in the Battle of Mohács represents only one among them, so it remains unclear whether they were a soldier fighting in the battle, a member of the Hungarian camp or a civilian from a nearby settlement, etc. Likewise, the exact circumstances of MMG3-75’s death remain uncertain – whether they perished in the battle, died while retreating or were executed afterwards, etc. To clarify this point in the manuscript, we have inserted two sentences immediately following the relevant part of the *Materials* section (lines 150–156 in the revised version of our manuscript):

“*It is important to note that, to date, several hypotheses have been proposed regarding the identity of the individuals buried in the five known mass graves at the Mohács National Memorial Site, suggesting that they may have been soldiers, members of the Hungarian camp or civilians from nearby settlements [77,79]. Ongoing research seeks to reassess these hypotheses and to shed further light on both the identities of these individuals and the circumstances of their death – whether they perished in the battle, died while retreating or were executed afterwards.*”.

In addition, it should be emphasised that the Hungarian (Christian) army at the Battle of Mohács was a heterogeneous and socially diverse force, reflecting the feudal levy system and the extraordinary mobilisation pressures of the time (Nagy-L, 2024). It included the king’s personal retinues, banner units raised by magnates and lesser nobility, peasants and other dependents under their authority, as well as foreign mercenary contingents recruited from German, Czech, and other regions. The composition of the army thus ranged from professional or noble soldiers to conscripted civilians and peasants, with considerable variation in equipment, training, and battlefield role among these groups.

From the summer of 1526, the Hungarian crown increasingly resorted to desperate measures to summon troops against the advancing Ottoman forces (Kovács, 2023; Nemes, 2024). Royal orders mandated, in stages, that nobles provide men from among their peasants: initially every fifth, then every second, and ultimately all able-bodied subjects – including the sick, infirm, and even monks – were required to join the army. These summons were accompanied by the ceremonial display of the “bloody sword” (an ancient signal of military levy), which communicated the urgency of the measure and the severe consequences for noncompliance, including accusations of treason, confiscation of property, and execution.

Taken together, this suggests that even individuals with serious health conditions could have been present in the Hungarian forces, either as part of formal military units (professional soldiers or members of a feudal banner) or as conscripted levies (civilians or peasants). Accordingly, MMG3-75 cannot definitively be interpreted as a professional soldier; this younger adult male could have belonged to any of these groups and, despite severe illness, been present within the army.

While it remains uncertain whether MMG3-75 was actually present as a soldier during the Battle of Mohács, even if he was, the severe pathological condition we infer (i.e., tuberculous clival osteomyelitis with associated meningitis) would not necessarily have prevented him from taking an active role either in the main engagement of the battle or in the defence of the camp. Patients with tuberculous clival osteomyelitis, sometimes complicated by tuberculous meningitis, are typically young adults who develop seemingly mild or nonspecific signs and symptoms such as headache, fever, vomiting or weight loss over a period of several months (usually 1–3, but sometimes up to 6–8 months) (Sagar & colleagues, 2018; Flynn & co-workers, 2022; Iyer & colleagues, 2022; Karim & co-workers, 2025). Despite this apparently benign clinical picture at presentation, radiological investigations often reveal extensive and severe intracranial involvement. Therefore, while MMG3-75’s illness may have affected his physical capacity, it cannot be taken as definitive evidence that he was unable to participate in the army, especially considering that, at the time of conscription or even during the battle itself, he may have still been in the relatively benign phase of his disease, and thus would not necessarily have appeared, or felt, severely ill. All of this, however, remains entirely hypothetical.

We hope this clarification resolves any ambiguity regarding the context of the burial and the identity of MMG3-75.

**REFERENCES:**

Bertók G, Neményi R, Pálfi G, Simon B. 2022. A mohácsi III. számú tömegsír új kutatása. *Magyar Régészet* **11**(1): 44–53. DOI: [10.36245/mr.2022.1.2](https://doi.org/10.36245/mr.2022.1.2)

Bertók G, Neményi R, Simon B. Az 1526. évi mohácsi csatához köthető tömegsírok felfedezésének története és a III. számú tömegsír új feltárásának eredményei. [The history of the discovery of the mass graves associated with the Battle of Mohács in 1526 and the achievements of the new excavation of mass grave No. 3.]. In: Varga S, editor. *Elsüllyedt Mohács. Újabb Tanulmányok a Mohácsi Csatával Kapcsolatos Kutatások Eredményeiből. [Sunken Mohács. New Studies Using the Research Achievements Relating to the Battle of Mohács.]*. Budapest, Hungary: Martin Opitz Kiadó; 2023. pp. 9–19.

Drusza T. A mohácsi csata rekonstrukciója. Új adatok – új megközelítés – új hipotézis. [Reconstruction of the Battle of Mohács. New data – new approach – new hypothesis.]. In: Varga S, editor. *Temetetlen Mohács. Az 1526. és az 1687. évi csata új kutatási eredményei*. *[Unburied Mohács. New Research Results on the Battles of 1526 and 1687.]*. Budapest, Hungary: Martin Opitz Kiadó; 2024. pp. 463–502.

Flynn WP, Ntuli Y, Zhang H, Tiberi S. 2021. A case of clival tuberculosis and associated meningitis. *Journal of Clinical Tuberculosis and Other Mycobacterial Diseases* **25**: 100273. DOI: [10.1016/j.jctube.2021.100273](https://doi.org/10.1016/j.jctube.2021.100273)

Iyer AS, Patil PV, Pandey D, Kute BS, Shetty BB. 2022. Tubercular skull base osteomyelitis – A case report. *IDCases* **27**: e01360. DOI: [10.1016/j.idcr.2021.e01360](https://doi.org/10.1016/j.idcr.2021.e01360)

Karim S, Kurup B, Solomon R, Shobhavat L, Gandhi D, Verma S. 2025. Nasopharyngeal mass with skull base invasion due to tuberculosis. *Indian Journal of Pediatrics* **92**(10): 1129. DOI: 10.1007/s12098-025-05665-w

Kovács V. A véres kard körülhordozása a Magyar Királyságban Mohács előestéjén. [Carrying around the Bloody Sword before the Battle of Mohács.]. In: Varga S, editor. *Elsüllyedt Mohács. Újabb Tanulmányok a Mohácsi Csatával Kapcsolatos Kutatások Eredményeiből. [Sunken Mohács. New Studies Using the Research Achievements Relating to the Battle of Mohács.]*. Budapest, Hungary: Martin Opitz Kiadó; 2023. pp. 145–154.

K. Zoffmann Z. *Az 1956-os mohácsi csata 1976-ban feltárt tömegsírjainak embertani vizsgálata*. Budapest, Hungary: Akadémiai Kiadó; 1982.

Nagy-L I. A mohácsi csata 4D-ben. Adalékok a csata harcászati rekonstrukciójához. [The Battle of Mohács in 4D. Additions to the reconstruction of the tactics of the battle.]. In: Varga S, editor. *Temetetlen Mohács. Az 1526. és az 1687. évi csata új kutatási eredményei*. *[Unburied Mohács. New Research Results on the Battles of 1526 and 1687.].* Budapest, Hungary: Martin Opitz Kiadó; 2024. pp. 443–462.

Nemes G. A győri székeskáptalan bandériuma a késő középkorban. [The banderium of the Cathedral Chapter of Győr in the Late Middle Ages.]. In: Varga S, editor. *Temetetlen Mohács. Az 1526. és az 1687. évi csata új kutatási eredményei*. *[Unburied Mohács. New Research Results on the Battles of 1526 and 1687.].* Budapest, Hungary: Martin Opitz Kiadó; 2024. pp. 193–203.

Papp L. A mohácsi csatahely kutatása. In: Dombay J, editor. A *Janus Pannonius Múzeum Évkönyve 1960*. Pécs, Hungary: Janus Pannonius Museum; 1961. pp. 197–251.

Sagar P, Raj A, Wadhwa V, Singh I. 2018. Tubercular osteomyelitis of the clivus. *The Turkish Journal of Ear Nose Throat* **28**(1): 44–47. DOI: [10.5606/Tr-ENT.2018.49932](http://dx.doi.org/10.5606/Tr-ENT.2018.49932)

Tihanyi B, Samu L, Koncz I, Hergott K, Medgyesi P, Pálfi G, Szabó KÁ, Kis L, Marcsik A, Molnár E, Spekker O. 2024. A glimpse into the the past of Hansen’s disease – Re-evaluation and comparative analysis of cases with leprosy from the Avar period of the Trans-Tisza region, Hungary. *Tuberculosis* **148**: 102552. DOI: 10.1016/j.tube.2024.102552

Varga S. Megjegyzések a mohácsi csata utáni kivégzések kérdéséhez. [Comments on the executions after the Battle of Mohács.]. In: Varga S, editor. *Elsüllyedt Mohács. Újabb Tanulmányok a Mohácsi Csatával Kapcsolatos Kutatások Eredményeiből. [Sunken Mohács. New Studies Using the Research Achievements Relating to the Battle of Mohács.]*. Budapest, Hungary: Martin Opitz Kiadó; 2023. pp. 107–116.

Viczián I, Szeberényi J. A mohácsi csata igaz története, helyszíneinek és eseményeinek földrajzi rekonstrukciója [The true history of the Battle of Mohács: A geographical reconstruction of its sites and events.]. In: Varga S, editor. *Temetetlen Mohács. Az 1526. és az 1687. évi csata új kutatási eredményei*. *[Unburied Mohács. New Research Results on the Battles of 1526 and 1687.]*. Budapest, Hungary: Martin Opitz Kiadó; 2024. pp. 411–442.

4) “*The suggestion that you should look at TB-related lipids appears to be an excellent one, as it may make your original suggestion of clival TB a more acceptable primary diagnosis.*”.

We fully agree with your suggestion that the investigation of tuberculosis-related lipid biomarkers would be highly beneficial. Such analyses could indeed provide important additional evidence and make tuberculous clival osteomyelitis a more acceptable primary diagnosis. We consider this a valuable direction for future research and intend to carry out lipid biomarker analyses once the necessary resources and conditions become available.

In addition, following **Reviewer 3**’s recommendations, we have created a separate *Conclusions* section, distinct from the *Discussion*, where we now provide a detailed summary of our study’s limitations. Among these limitation points, we explicitly refer to the lack of lipid-based analyses as an important aspect for future research (lines 801–815 in the revised version of our manuscript):

“*Although the endogenous human DNA content recovered from the two hard tissue samples in MMG3-75 was less than 0.01%, indicating generally poor biomolecular preservation in this younger adult male, this does not necessarily preclude the potential success of alternative molecular diagnostic approaches, such as lipid biomarker analyses. Lipid biomarker-based investigations could provide valuable additional evidence to support the morphology-based diagnosis of TB in skeletal human remains – particularly in cases where endogenous DNA preservation is poor, as in MMG3-75. However, like aDNA analyses, these methods also require invasive sampling, preferably from the lesion-affected bone or areas adjacent to it. Targeted sampling of these bone regions would be crucial to demonstrate a direct causal link between the clival alterations and any detected remnants of Mycobacterium tuberculosis DNA, and hence between these bony changes and TB itself. Nonetheless, because of the unique nature of the clival lesions observed in MMG3-75, we believe that preservation currently outweighs the potential benefits of further invasive molecular investigations. We remain committed, however, to exploring these approaches in the future, once the necessary resources and conditions (e.g., less-destructive sampling techniques) become available;*”.

**Responses to the reviewers’ comments:**

1) **Reviewer 1** commented that “*The paper describes a supposed case of tuberculous osteomyelitis of the clivus in a male individual from a mass grave dated back to the 16th century. The manuscript is well written, but there are crucial criticisms according to which the final diagnosis cannot be supported. The authors start from a pre-defined diagnosis (already anticipated at the beginning of the results!) and continue by proposing it without strong scientific evidence; the differential diagnosis of the lesions to the clivus is oriented to exclude other pathologies, but without strong justification. In summary, as the lesions to the clivus can be caused by many other conditions, and as GIs are not pathognomonic of TB meningitis but only of TB infection (and not disease), the diagnosis of clivus TB osteomyelitis is not strongly supported and lacks empirical evidence.*”. And “*In conclusion, as the lesions to the clivus can be caused by other diseases, and in the case of MMG3-75 there is no strong support that they are caused by TB, as well as the fact that GIs are not pathognomonic of TB meningitis, the diagnosis proposed in this paper is too weak to be sustained.*”.

We would like to thank you for the time and effort dedicated to evaluating our manuscript, and for providing a detailed and critical assessment. We appreciate your comments and acknowledge the opportunity to clarify and further substantiate several aspects of our study.

While we respectfully disagree with some of your interpretations and conclusions, we have carefully considered each point raised and have provided detailed responses below. Wherever possible, we have revised our manuscript to improve clarity and to address your concerns, or we have explained why we believe certain criticisms are not applicable in this specific case.

We hope that our point-by-point responses will help to clarify our reasoning and demonstrate that our diagnostic interpretation is supported by (macromorphological) evidence and a thorough differential diagnosis.

2) **Reviewer 1** noted that “*There are no pathognomonic features of skeletal TB in MMG3-75: no characteristic lesions to the vertebrae, ribs, or any other skeletal elements; by admission of the authors, TB osteomyelitis of the clivus is a very rare clinical entity and should be accompanied by lesions of the CVJ, which are absent in the present case. The lesions observed in the skeleton MMG3-75 could be attributed to other pathologies.*”.

We sincerely thank you for the detailed and thoughtful comments on the diagnosis of tuberculosis in MMG3-75, which we have carefully considered and address point by point below.

a) As for your comment “*There are no pathognomonic features of skeletal TB in MMG3-75: no characteristic lesions to the vertebrae, ribs, or any other skeletal elements;*”, we respectfully note that it is not entirely clear to us why the presence or, in this case, the absence of vertebral or costal changes characteristic of skeletal tuberculosis would necessarily affect the interpretation of the clival lesions and endocranial alterations observed in MMG3-75 as potentially being of tuberculous origin.
   (1) Tuberculosis is a highly variable disease: (1) in many cases, it manifests solely in the lungs (i.e., pulmonary tuberculosis); (2) in others, the pulmonary form is present simultaneously with one or more extra-pulmonary manifestations (e.g., tuberculous pleurisy, skeletal tuberculosis, or tuberculous meningitis); and (3) there are cases without pulmonary involvement but with one or more concurrent extra-pulmonary forms. Importantly, this variability means that not every tuberculosis case presents with the pulmonary manifestation and/or one or more extra-pulmonary forms concurrently.

In MMG3-75, the clival lesions (mild cortical erosion and multiple, well-circumscribed osteolytic lesions) may represent an extra-pulmonary manifestation (i.e., tuberculous clival osteomyelitis, a subtype of extra-spinal tuberculous osteomyelitis, which is a rare form of skeletal tuberculosis), whereas the endocranial granular impressions and abnormal blood vessel impressions are consistent with another extra-pulmonary manifestation (i.e., tuberculous meningitis, which is not a subtype of skeletal tuberculosis but instead a form of central nervous system tuberculosis). In contrast, the alterations referred to in your comment, such as vertebral and costal changes, would be consistent with other manifestations of tuberculosis: vertebral changes would indicate spinal tuberculosis, the most frequent subtype of skeletal tuberculosis, whereas the costal changes would correspond to extra-spinal tuberculous osteomyelitis of the ribs. However, the forms of skeletal tuberculosis underlying vertebral and costal changes (i.e., spinal tuberculosis and extra-spinal tuberculous osteomyelitis of the ribs) do not necessarily occur concurrently with the manifestations that can account for the cranial alterations observed in MMG3-75 (i.e., tuberculous meningitis and tuberculous clival osteomyelitis). Accordingly, it is not surprising that no additional lesions characteristic of skeletal tuberculosis (e.g., vertebral or costal changes) were observed in MMG3-75.

Another possible reason for the absence of the alterations mentioned in your comment is that MMG3-75 may have died before the forms of skeletal tuberculosis responsible for producing these bony changes (i.e., spinal tuberculosis and extra-spinal tuberculous osteomyelitis of the ribs) had sufficient time to generate detectable skeletal lesions. It is also possible that such manifestations of the disease did not have time to develop at all before this younger adult male’s death.

Accordingly, the absence of the alterations referred to in your comment – such as vertebral changes suggestive of spinal tuberculosis or costal changes indicative of extra-spinal tuberculous osteomyelitis of the ribs – does not preclude a tuberculous origin for the cranial lesions detected in MMG3-75.

(2) It is also important to note that even if the alterations mentioned in your comment had been observed in MMG3-75, none of them could be considered specific for tuberculosis. Each would require a differential diagnosis, as we applied for the clival lesions in MMG3-75. For instance, vertebral changes could be caused not only by spinal tuberculosis but also by other granulomatous spinal infections (e.g., fungal infections such as candidiasis, aspergillosis, coccidioidomycosis, and blastomycosis; and bacterial infections such as actinomycosis and brucellosis), pyogenic spinal infections, compression fractures, spinal bone metastases, or multiple myeloma (Aufderheide & Rodríguez-Martín, 1998; Ortner, 2003; Agrawal & colleagues, 2006; Garg & Somvanshi, 2011; Pigrau-Serrellach & Rodríguez-Pardo, 2013; Rivas-Garcia & co-workers, 2013; Esteves & colleagues, 2017; Dean & co-workers, 2019). Nevertheless, even if tuberculosis could be established as the primary diagnosis for these skeletal lesions (e.g., spinal tuberculosis in the case of vertebral changes or extra-spinal tuberculous osteomyelitis of the ribs in the case of costal changes), this would not necessarily bring us closer to confirming a tuberculous origin for the cranial lesions detected in MMG3-75.

As noted above, the alterations referred to in your comment and the cranial bony changes observed in MMG3-75 correspond to different manifestations of tuberculosis. Consequently, neither spinal tuberculosis nor extra-spinal tuberculous osteomyelitis of the ribs could account for the clival bony changes or endocranial alterations, as the former would explain only vertebral changes and the latter only costal changes. Therefore, no direct causal link could be established between the skeletal lesions referred to in your comment and those detected in the skull of MMG3-75.

As **Reviewer 3** also highlighted (“*The first one is the lack of evidence that supports the hypothesis of any relationship between the lesions at the clivus and the other endocranial lesions.*”), even the presence of endocranial granular impressions, which are tuberculosis-specific bony changes indicative of tuberculous meningitis, is not sufficient to assert that the clival lesions in MMG3-75 were caused by tuberculosis. Although we outlined two possible pathogenetic pathways by which the tuberculous infection could have spread from the clivus to the meninges, or vice versa, and how the sequential development of the cranial bony changes (both the clival and endocranial alterations) observed in MMG3-75 might have occurred, the presence of endocranial granular impressions serves only as indirect evidence supporting the hypothesis that the clival lesions were also caused by tuberculosis (specifically tuberculous clival osteomyelitis).

This is because the presence of granular impressions in MMG3-75 provides corroborative (macromorphological) evidence only for tuberculous meningitis, which cannot account for the clival bony changes, and not for concurrent tuberculous clival osteomyelitis, which could explain these alterations. This is precisely why it was necessary to perform a detailed differential diagnosis of the clival lesions detected in MMG3-75.

As suggested by **Reviewer 3**, we have now included a separate *Conclusions* section in the revised version of our manuscript (lines 759–767). In this section, we provide a detailed overview of our study’s limitations, including this aspect:

“*Nonetheless, a direct causal link between TB and the clival lesions cannot be established, as the presence of GIs in MMG3-75 provides (macromorphological) evidence only for TBM, which cannot account for the clival bony changes, and not for concurrent TB clival osteomyelitis, which could explain these alterations. Therefore, while TB clival osteomyelitis is indeed a plausible underlying cause of the observed clival lesions, we cannot conclusively assert that it occurred alongside TBM in this younger adult male, nor can we exclude the possibility that another pathological condition concomitantly present with TB in MMG3-75 contributed to the formation of these alterations.*”.

b) As for your comment “*The lesions observed in the skeleton MMG3-75 could be attributed to other pathologies.*”, we fully agree with you that this is a crucial consideration, and we carefully addressed it when establishing the diagnosis in MMG3-75. Based on the macromorphological characteristics and co-occurrence of the clival lesions detected in this younger adult male (mild cortical erosion and multiple, well-circumscribed osteolytic lesions), tuberculous clival osteomyelitis was initially proposed as a hypothetical diagnosis. This interpretation was further supported by the presence of tuberculosis-specific granular impressions in multiple locations on the endocranial surface, which provided corroborative (macromorphological) evidence that MMG3-75 suffered from tuberculosis (specifically tuberculous meningitis) at the time of death.

Nonetheless, we were fully aware that this does not constitute direct causal link between tuberculosis and the observed clival bony changes. As mentioned above, the presence of endocranial granular impressions in MMG3-75 does not demonstrate that tuberculous clival osteomyelitis – the manifestation of tuberculosis that could explain the detected clival alterations – occurred concomitantly with tuberculous meningitis in this younger adult male; it merely increases the likelihood that both forms of the disease may have been present. Consequently, while tuberculous clival osteomyelitis represents a plausible underlying cause of the observed clival lesions, we cannot definitively assert that it co-occurred with tuberculous meningitis in this younger adult male. Likewise, we cannot entirely rule out the possibility that another pathological condition, presenting simultaneously with tuberculosis in MMG3-75, contributed to the formation of these bony changes.

This consideration made a detailed differential diagnosis of the clival bony changes detected in MMG3-75 indispensable. During this process, we evaluated both non-infectious and infectious aetiologies. Non-infectious causes included clival malignancies, such as chordoma, chondrosarcoma, and bone metastases, while infectious causes encompassed pyogenic osteomyelitis (e.g., due to *Staphylococcus* or *Pseudomonas* spp.) and fungal osteomyelitis (e.g., caused by *Aspergillus* or *Mucor* spp.) [24,26,50,53,64-65]. Taking into account the macromorphological characteristics of the clival alterations (e.g., location, and the absence of marginal sclerosis, reactive new bone formation in the surrounding bone, and sequestrum), along with the individual’s age-at-death and sex, we were able to either exclude these alternative pathological conditions or consider them less likely to account for the observed clival lesions. Consequently, tuberculous clival osteomyelitis emerged as the most plausible – and therefore primary – diagnosis for MMG3-75.

We would like to note that **Reviewer 4** expressed a very positive opinion regarding our differential diagnosis section, identifying it as one of the strengths of the manuscript (“*The strength of this paper is in listed and explained differential diagnoses…*”). **Reviewer 2** also gave a favourable evaluation, describing the section (lines 436–510 in the original version of our manuscript) as “*thorough, scientifically accurate, and providing further assurance for the results*”.

c) As for your comment “*by admission of the authors, TB osteomyelitis of the clivus is a very rare clinical entity and should be accompanied by lesions of the CVJ, which are absent in the present case.*”, it appears that there may be a misunderstanding/misinterpretation. As we highlighted in the *Introduction* section of the original version of our manuscript (lines 108–110): “*Tuberculous involvement of the clivus usually results from contiguous spread of the infection from the CVJ [21,59-60]; TB clival osteomyelitis sparing the CVJ is extremely rare [50,59,61-63]*.”.

This indicates that, based on the modern medical literature, in the majority of reported cases, the tuberculous infection spreads contiguously from the cranio-vertebral junction to the clivus, subsequently leading to tuberculous clival osteomyelitis. However, this does not imply that this is the only possible route of infection – in at least four published cases (Shenoy & Raja, 2004; Bhavanam & colleagues, 2014; Gandhi & co-workers, 2022; Kharosekar & colleagues, 2025), it was explicitly stated that the cranio-vertebral junction was not affected by the disease. Beyond contiguous spread from the cranio-vertebral junction, other potential pathways that have been proposed in the modern medical literature include haematogenous dissemination and extension from adjacent pharyngeal lymph nodes (Devi & co-workers, 2003; Shenoy & Raja, 2004; Richardus & colleagues, 2011; Bhavanam & co-workers, 2014; Sagar & colleagues, 2018; Gandhi & co-workers, 2022; Karim & colleagues, 2025; Kharosekar & co-workers, 2025).

In other words, the absence of skeletal lesions at the cranio-vertebral junction of MMG3-75 does not preclude the diagnosis of tuberculous clival osteomyelitis. It should also be taken into account that less than two dozen cases of tuberculous clival osteomyelitis have been documented in the modern medical literature to date (Selvapandian & Chandy, 1993; Devi & colleagues, 2003; Shenoy & Raja, 2004; Mancusi & co-workers, 2009; Richardus & colleagues, 2011; Bhavanam & co-workers, 2014; Joshi & colleagues, 2017; Sagar & co-workers, 2018; Shashidhar & colleagues, 2018; Tiberi & co-workers, 2018; Flynn & colleagues, 2021; Kumar & co-workers, 2021; Ghandi & colleagues, 2022; Iyer & co-workers, 2022; Rohini & colleagues, 2022; Karim & co-workers, 2025; Kharosekar & colleagues, 2025), making it difficult – and indeed inappropriate – to draw broad conclusions or establish definitive trends from such a limited number of observations. Furthermore, in a number of these reported cases, the involvement of the cranio-vertebral junction was not specified (neither its presence nor its absence was mentioned), and the precise route by which the clivus became infected by tuberculosis was not discussed at all.

To prevent potential misunderstandings or misinterpretations, such as those reflected in the comment quoted from you, and to clarify that the clivus can become infected by tuberculosis not only through contiguous extension from the cranio-vertebral junction but also via other routes (e.g., haematogenous spread), we have rephrased the relevant part of the *Introduction* section in the revised version of our manuscript (lines 108–112), which now reads as follows:

“*Tuberculous involvement of the clivus usually results from contiguous spread of the infection from the CVJ [21,59-60**], while TB clival osteomyelitis sparing the CVJ is rare [50,59,61-65]. It has been suggested that the clivus may also become infected by TB bacilli via alternative routes, such as haematogenous dissemination or extension from adjacent pharyngeal lymph nodes [23,50,53,59,61,65-67].*”.

We hope that these clarifications help to elucidate certain aspects of our study, contribute to a clearer understanding of our reasoning, and resolve any potential misunderstandings concerning the diagnosis of tuberculous clival osteomyelitis in MMG3-75.

**REFERENCES:**

Agrawal A, Timothy J, Shetty MS, Shetty L, Shetty JP. 2006. Paediatric perspective of spinal tuberculosis. *Paediatrics Today* **9**(1): 25–34.

Aufderheide AC, Rodríguez-Martín C. *The Cambridge encyclopedia of human paleopathology*. Cambridge, UK: Cambridge University Press; 1998. pp. 118–141.

Bhavanam SH, Rajesh A, Uppin MS. 2014. Tubercular osteomyelitis of spheno-clival region presenting with lateral rectus palsy. *Neurology India* **62**(2): 198–200. DOI: 10.4103/0028-3886.132400

Dean A, Zyck S, Toshkezi G, Galgano M, Marawar S. 2019. Challenges in the diagnosis and management of spinal tuberculosis: case series. *Cureus* **11**(1):e3855. DOI: [10.7759/cureus.3855](https://doi.org/10.7759/cureus.3855)

Devi BI, Tyagi AK, Bhat DI, Santosh V. 2003. Tuberculous osteitis of clivus. *Neurology India* **51**(1): 69–70.

Esteves S, Catarino I, Lopes D, Sousa C. 2017. Spinal tuberculosis: rethinking an old disease. *Journal of Spine* **6**(1): 358. DOI: 10.4172/2165-7939.1000358

Flynn WP, Ntuli Y, Zhang H, Tiberi S. 2021. A case of clival tuberculosis and associated meningitis. *Journal of Clinical Tuberculosis and Other Mycobacterial Diseases* **25**: 100273. DOI: [10.1016/j.jctube.2021.100273](https://doi.org/10.1016/j.jctube.2021.100273)

Gandhi AS, Nadkarni TD, Balasubramaniam S. 2022. Clival tuberculosis. A case report. *Neurology India* **70**(2): 775–777. DOI: [10.4103/0028-3886.344665](https://doi.org/10.4103/0028-3886.344665)

Garg RK, Somvanshi DS. 2011. Spinal tuberculosis: a review. *The Journal of Spinal Cord Medicine* **34**(5): 440–454. DOI: 10.1179/2045772311Y.0000000023

Iyer AS, Patil PV, Pandey D, Kute BS, Shetty BB. 2022. Tubercular skull base osteomyelitis – A case report. *IDCases* **27**: e01360. DOI: [10.1016/j.idcr.2021.e01360](https://doi.org/10.1016/j.idcr.2021.e01360)

Joshi VP, Valsangkar S, Valsangkar A, Dekhne A, Nivargi S, Agrawal A. 2017. Tuberculosis of the clivus. *Indian Journal of Neurosurgery* **6**: 72–75. DOI: [10.1055/s-0036-1588032](http://dx.doi.org/10.1055/s-0036-1588032)

Karim S, Kurup B, Solomon R, Shobhavat L, Gandhi D, Verma S. 2025. Nasopharyngeal mass with skull base invasion due to tuberculosis. *Indian Journal of Pediatrics* **92**(10): 1129. DOI: 10.1007/s12098-025-05665-w

Kharosekar H, Mankar Y, Pujara R, Pujara B, Rathi S. 2025. Primary spheno-clival tuberculoma – Rare lesion. *Clinical Neuroscience and Neurological Research International Journal* **6**(1): 180071. DOI: 10.63235/CNNRIJ.180071

Kumar S, Kumar A, Gadhavi H, Maheshwari V. 2021. Tubercular skull base osteomyelitis in an immunocompetent individual: A rare entity. *Interdisciplinary Neurosurgery* **24**: 100816. DOI: [10.1016/j.inat.2020.100816](https://doi.org/10.1016/j.inat.2020.100816)

Mancusi G, Marks B, Czerny C, Thalhammer F, Thurnher D, Riedl M, Dekan G, Knerer B 2005. Tuberkulöse Osteomyelitis des Klivus mit Beteiligung des Nasopharynx [Tuberculosis of the clivus and the nasopharynx]. *HNO* **53**(12): 1081–1084. DOI: [10.1007/s00106-005-1279-1](https://doi.org/10.1007/s00106-005-1279-1)

Ortner DJ. Infectious diseases: tuberculosis and leprosy. In: Ortner DJ, editor. *Identification of pathological conditions in human skeletal remains*. San Diego, CA, USA: Academic Press; 2003. pp. 227–271.

Pigrau-Serrallach C, Rodríguez-Pardo D. 2013. Bone and joint tuberculosis. *European Spine Journal* **22**(Suppl. 4): S556–S566. DOI: 10.1007/s00586-012-2331-y

Richardus RA, Jansen JC, Steens SCA, Arend SM. 2011. Two immigrants with tuberculosis of the ear, nose, and throat region with skull base and cranial nerve involvement. *Case Reports in Medicine* **2011**: 675807. DOI: 10.1155/2011/675807

Rivas-Garcia A, Sarria-Estrada S, Torrents-Odin C, Casas-Gomila L, Franquet E. 2013. Imaging findings of Pott’s disease. *European Spine Journal* **22**(Suppl. 4): S567–S578. DOI: 10.1007/s00586-012-2333-9

Rohini R, Badole P, Pati SK, Meher J, Venkat N. 2022. Tuberculous skull base osteomyelitis with cerebral venous sinus thrombosis in an immunocompetent adolescent: A case report. *Cureus* **14**(4): e23865. DOI: 10.7759/cureus.23865

Sagar P, Raj A, Wadhwa V, Singh I. 2018. Tubercular osteomyelitis of the clivus. *The Turkish Journal of Ear Nose and Throat* **28**(1): 44–47. DOI: [10.5606/Tr-ENT.2018.49932](http://dx.doi.org/10.5606/Tr-ENT.2018.49932)

Selvapandian S, Chandy MJ. 1993. Tuberculous granuloma of the clivus. *British Journal of Neurosurgery* **7**(5): 581–582. DOI: [10.3109/02688699308995084](https://doi.org/10.3109/02688699308995084)

Shashidhar A, Bharath RD, Rao MB, Arivazhagan A. 2018. Clival lesion in a young girl: Neoplastic or inflammatory?. *Pediatric Neurosurgery* **53**(4): 280–281. DOI: [10.1159/000489011](https://doi.org/10.1159/000489011)

Shenoy SN, Raja A. 2004. Tuberculous granuloma of the spheno-clival region. *Neurology India* **52**(1): 129–130.

Tiberi S, Payen MC, Manika K. 2018. Clinical cases in tuberculosis. *ERS Monographs* **82**: 446–460.

3) **Reviewer 1** noted that “*There are many other conditions that can cause erosion and lesions on the clivus, as highlighted by the authors in the differential diagnosis, but they exclude them without a strong justification or clear rationale. For example, they exclude skull-base chondrosarcoma because the erosion in MMG3-75 is mild and there are well-circumscribed osteolytic lesions, but they do not consider that this individual could present an initial stage of the tumour. Again, they exclude other conditions based on their clinical rarity, but the TB clivus osteomyelitis is extremely rare as well.*”. A similar concern was also raised by **Reviewer 3**, who noted that “*The differential diagnosis at the end of the paper rejects some possible explanations for other causes of these lesions, arguing that they are very rare, but also that the clivus lesions in TB are. Therefore, the evidence offered is not quite convincing.*”.

We thank both of you for this thoughtful and constructive comment. However, it seems that our reasoning in the original version of the manuscript (lines 436–510) may have been misunderstood or misinterpreted. We did not intend to imply that, during the detailed differential diagnosis of the clival alterations observed in MMG3-75 (mild cortical erosion and multiple, well-circumscribed osteolytic lesions), the other possible aetiologies considered alongside tuberculous clival osteomyelitis (chordoma, chondrosarcoma, bone metastases, and pyogenic or fungal osteomyelitides) were excluded or regarded as less likely based on their rarity. Rather, this assessment was grounded in the macromorphological characteristics of the osteolytic lesions (e.g., localisation, and the absence of marginal sclerosis, reactive new bone formation or sequestrum) and the estimated age-at-death of MMG3-75 (30–39 years), which we believe are more consistent with tuberculous clival osteomyelitis and thus make it the most likely primary diagnosis in this younger adult male. We are, of course, fully aware that not only the other possible aetiologies considered, but also tuberculous clival osteomyelitis itself represents an extremely rare pathological condition. This is precisely why we consider the presentation and publication of this case (MMG3-75) to be of particular importance.

As mentioned above, **Reviewer 4** regarded our differential diagnosis section very positively, highlighting it as one of the key strengths of the manuscript (“*The strength of this paper is in listed and explained differential diagnoses…*”). **Reviewer 2** also evaluated this section favourably, describing it (lines 436–510 in the original version of our manuscript) as “*thorough, scientifically accurate, and providing further assurance for the results*”.

Nevertheless, to further strengthen the scientific justification of our diagnosis (i.e., tuberculous clival osteomyelitis as the primary diagnosis), we have expanded the description of the aetiologies considered in the differential diagnosis, with particular emphasis on the macromorphological characteristics of the osteolytic lesions:

“*… in TB osteomyelitis, the osteolytic lesions are typically well-defined, with minimal or no marginal sclerosis and little or no reactive new bone formation in the surrounding bone, while sequestration is relatively uncommon [70-73].*” (tuberculous clival osteomyelitis; lines 114–116 in the revised version of our manuscript);

“*… which cause extensive lytic bone destruction without evidence of surrounding sclerosis* *(i.e., well-defined or ill-defined osteolytic lobulated lesions without sclerotic margins) [54-55,58,159,165,167,170-172].*” (clival chordoma; lines 580–582 in the revised version of our manuscript);

“*… which cause lytic bone destruction* *(i.e., ill-defined osteolytic lesions) [159,171-172,177-178,180,186,189].*” (clival chondrosarcoma; lines 611–612 in the revised version of our manuscript);

“*… the disease can be classified as 1) (predominantly) osteolytic, 2) (predominantly) osteoblastic, or 3) mixed [172,195,197,199,201-202]; consequently, it exhibits highly variable morphological patterns and radiological appearances [172].*” (clival bone metastasis; lines 645–647 in the revised version of our manuscript); and

“*In pyogenic osteomyelitis, osteolytic lesions are typically ill-defined, often with thick sclerotic margins; reactive new bone formation in the surrounding bone and sequestrum are common [71-73,239-240]. Fungal osteomyelitis can show similar features (e.g., ill-defined osteolytic lesions or marginal sclerosis), but they are less consistent and may be absent [240-241].*” (pyogenic and fungal clival osteomyelitides; lines 690–694 in the revised version of our manuscript).

We would also like to highlight that **Reviewer 2** recommended placing clearer emphasis on the similarities and differences between our case (MMG3-75) and the alternative diagnoses (chordoma, chondrosarcoma, bone metastases, and pyogenic or fungal osteomyelitides) to improve clarity for the reader. Accordingly, we have revised the relevant part of our manuscript so that at the end of each discussion of a potential aetiology, explicit comparisons are provided with MMG3-75, outlining the main features that support or argue against each pathological condition:

“*The biological sex (male) and estimated age at death (30–39 years) of MMG3-75 do not argue against the diagnosis of clival chordoma, as these malignant bone tumours show no sex predilection and most commonly affect individuals belonging to the 20–40-year age group. In contrast, the osteolytic lesions in MMG3-75, although also lacking surrounding sclerosis as typically seen in this tumour type, display a markedly different spatial pattern. They are neither situated along the midline nor confined to the spheno-occipital synchondrosis but are instead scattered across the clivus of this younger adult male in an apparently random manner (Figs 3–4 and 8–9). Furthermore, the three larger osteolytic lesions identified within the clivus of MMG3-75 are clearly separated from one another and show no evidence of interconnection,* *thus lacking the lobulated appearance that would be expected in a clival chordoma (Fig 9). Taken together,* *the combination of the spatial distribution pattern and non-lobulated morphology of the osteolytic lesions observed in this younger adult male is difficult to reconcile with a clival chordoma, which typically arises from the spheno-occipital synchondrosis and presents as a centrally located (midline), well-circumscribed, expansile, lobulated soft-tissue mass. Based on these observations, clival chordoma can be ruled out with high certainty as a diagnostic option in MMG3-75;*” (lines 585–600 in the revised version of our manuscript);

“*Although* *the biological sex (male) and estimated age at death (30–39 years) of MMG3-75 do not rule out the possibility of a skull-base (clival) chondrosarcoma, he falls slightly below the typical age range (middle-aged adults) for this tumour type. Furthermore, several morphological features, particularly the spatial distribution of the osteolytic lesions in the clivus of this younger adult male, argue against this diagnosis. The well-circumscribed lesions are neither laterally located (off-midline) nor confined to the petro-clival synchondroses, but are instead randomly scattered across the clivus of MMG3-75 (Figs 3–4 and 8–9), with the three larger osteolytic lesions being spatially separate and showing no signs of interconnection. Although a larger, non-perforating osteolytic lesion is present within the clivus of this younger adult male on each side, adjacent to the petro-clival synchondroses, a skull-base (clival) chondrosarcoma would be expected to arise in the synchondral cartilage and progressively erode the surrounding bone, compromising the cortical layers and producing perforating lesions. In contrast, CBCT images (Fig 8) reveal no evidence of erosion in the bones adjacent to either petro-clival synchondrosis (neither in the laterally adjacent petrous apex nor in the medially adjacent clivus), providing strong evidence that these osteolytic lesions did not develop from the synchondral cartilage inward into the surrounding bone. The observed spatial distribution of the osteolytic lesions detected in MMG3-75, together with the intact cortical bone adjacent to the petro-clival synchondroses and the lack of the characteristic ring-and-arc calcification pattern on the CBCT images (Fig 8), is difficult to reconcile with a skull-base (clival) chondrosarcoma, which typically presents as a laterally located (off-midline), well-circumscribed, expansile, lobulated soft-tissue mass with a ring-and-arc pattern of chondroid calcification, mostly arising from the petro-clival synchondrosis. Taken together, these observations strongly suggest that a skull-base (clival) chondrosarcoma is unlikely to account for the mild cortical erosion and multiple, well-circumscribed osteolytic lesions observed in MMG3-75;*” (lines 614–638 in the revised version of our manuscript);

“*Although the biological sex (male) of MMG3-75 is consistent with the slight male predominance reported for clival bone metastases, his estimated age at death (30–39 years) is younger than that of the older individuals typically affected by this pathological condition. Furthermore, beyond the clivus, no similar osteolytic lesions were identified elsewhere in the skull or postcranial skeleton of this younger adult male, whereas secondary bone malignancies usually present as multiple alterations, most often involving different parts of the axial skeleton. Finally, all clival changes in MMG3-75 are purely lytic, with no evidence of new bone formation. However, purely osteolytic or osteoblastic metastatic lesions are exceedingly rare; in most cases, both osteoclastic resorption and osteoblastic new bone formation are present to some extent, resulting in mixed alterations. Considering the relatively young estimated age at death of MMG3-75, the purely lytic nature of the clival lesions and their restriction to the clivus (with no similar alterations detected elsewhere in the axial skeleton), bone metastasis can be regarded as a less likely diagnosis in this younger adult male;*” (lines 656–669 in the revised version of our manuscript); and

“*The estimated age at death (30–39 years) of MMG3-75 is not in favour of the diagnosis of pyogenic or fungal skull-base (clival) osteomyelitides, as he is considerably younger than the elderly individuals typically affected by these pathological conditions. Moreover, these osteomyelitides usually arise secondary to ear or paranasal sinus infections, but CBCT images (Fig 8) show that both the middle and inner ears of MMG3-75 were intact, and the posterior wall of the sphenoid sinus – which became visible due to post-mortem damage – was also unaffected (Fig 4b). Although marginal sclerosis, reactive new bone formation, and sequestrum are frequently observed at the osteolytic lesions in such osteomyelitides, especially in pyogenic cases, none of these features were detected in this younger adult male, as demonstrated by the CBCT images (Fig 8). Considering the relatively young estimated age at death of MMG3-75, the absence of any evidence for preceding ear (i.e., otitis media or otitis interna) or paranasal sinus infection (i.e., sphenoid sinusitis), and the lack of marginal sclerosis, reactive new bone formation or sequestrum, it is highly improbable that a pyogenic or fungal skull-base (clival) osteomyelitis was responsible for the development of the mild cortical erosion and multiple, well-circumscribed osteolytic lesions observed in this younger adult male.*” (lines 695–710 in the revised version of our manuscript).

We hope these additions meet your expectations, and provide a clearer and more convincing argument.

As for your other comment (“*For example, they exclude skull-base chondrosarcoma because the erosion in MMG3-75 is mild and there are well-circumscribed osteolytic lesions, but they do not consider that this individual could present an initial stage of the tumour.*”), we would like to respectfully note that we are unsure of the source of the statement in your comment regarding the exclusion of clival chondrosarcoma “*because the erosion in MMG3-75 is mild and there are well-circumscribed osteolytic lesions*.” Nowhere in our manuscript do we make this claim.

It is possible that your misinterpretation of our discussion regarding clival chondrosarcoma stems from the way the information was presented in the original version of our manuscript (lines 458–472):

“*Chondrosarcomas constitute a rare, heterogeneous group of malignant bone tumours of the appendicular and axial skeleton, which make up about 20% of all primary bone malignancies [167-169]. The skull base is involved in about 1% of all chondrosarcoma cases, with a predilection for the synchondroses, especially the petro-clival synchondrosis [168-176]; most of these tumours are located off-midline [149,171,174,177]. Skull-base chondrosarcomas are slow-growing, locally aggressive tumours with a high local recurrence rate and low metastatic potential, which are considered to originate from multipotent mesenchymal cells or from embryonic cartilaginous remnants of the cranium [166-167,175-176,178-183]. They are usually laterally located (off-midline), well-circumscribed, expansile, lobulated soft-tissue masses with a ring-and-arc pattern of chondroid calcification, which cause lytic bone destruction [149,161,166-167,169,175,178]. Skull-base chondrosarcomas can develop at any age but tend to occur in middle-aged adults, and affect females and males equally [58,167,170,176,178,181]. Based on the above, it is unlikely that a skull-base chondrosarcoma resulted in the formation of the mild cortical erosion and the multiple, well-circumscribed osteolytic lesions detected at the clivus of MMG3-75.*”.

As **Reviewer 2** correctly noted, although the *Results* section of the original version of our manuscript provided a detailed description of the cranial alterations observed in MMG3-75, including the clival changes (mild cortical erosion and multiple, well-circumscribed osteolytic lesions), and the *Discussion and Conclusions* section of the original version of our manuscript offered a thorough account of each potential aetiology (including the types and macromorphological characteristics of clival lesions they can produce), we did not explicitly compare the bony changes observed in MMG3-75 with those typically associated with each potential pathological condition (i.e., chordoma, chondrosarcoma, bone metastases, and pyogenic or fungal osteomyelitides). Consequently, the reader was left to recall the lesion details from the *Results* section and mentally perform the comparison while reading the *Discussion* section, ultimately determining whether a given aetiology can be reasonably excluded as a possible diagnosis, or considered less likely than tuberculous clival osteomyelitis.

To address this issue, as noted above, we have now expanded the discussion of each potential aetiology to include a direct comparison with the lesions observed in MMG3-75, so that the reader no longer needs to perform this comparison mentally, thereby helping to prevent potential misunderstandings or misinterpretations, such as those reflected in the comment quoted from you.

We sincerely hope that these clarifications and the added comparisons improve the clarity of our differential diagnosis and address any concerns, and we thank you for the thoughtful comments that helped us strengthen our manuscript.

4) **Reviewer 1** commented that “*The authors support the diagnosis of TB osteomyelitis of the clivus by linking it to the presence of GIs on the endocranial surface. However, GIs cannot be a manifestation of TB meningitis simply because this is a very rare complication of TB with a very rapid outcome, of which an individual would not live long enough for skeletal manifestations to develop. In this case, the authors affirm that the lesions on the clivus developed following TB infection from the meninges; again, this is not possible as meningitis has a rapid clinical outcome, and the patient should have died before developing the erosion and lesions to the clivus, a process that requires a much longer time. In the paper of Genchi et al. (2025), GIs were found in half of the individuals examined from the Medieval population of Pava, and this strongly demonstrates that they are pathognomonic of TB infection and not TB meningitis; in fact, it is unreasonable to hypothesize that half of the population died from this very rare complication of the central nervous system. Considering that today the prevalence of TB meningitis is 1-5%, and that in the Medieval population of Pava GIs affected half of the skeletal assemblage, even admitting that in the past the prevalence of TB meningitis could have been higher than today, it is absolutely unlikely that the disease reached a prevalence of 50%. The pathophysiology of the human body cannot be changed during a few hundred years! Another point is that cases of protracted course of TB meningitis are reported in the modern clinical literature, but are absolutely exceptional; also, in this case, the authors cannot base their argumentation on rare exceptions, and again if TB meningitis is rapidly fatal today there are no reasons to suppose than in the past the course of this condition was longer enough to leave bone changes. Therefore, to support the hypothesis that GIs are a manifestation of TB meningitis is simply illogical. On the other hand, in the work of Spekker et al., 2020 carried out on the Robert J. Terry Anatomical Skeletal Collection, the prevalence of GIs was observed on individuals with a diagnosis of TB, not TB meningitis; this is a fundamental difference. Additionally, nor in that work nor in previous works reporting GIs as related to TB meningitis (Schultz, 1999; Schultz, 2001; Schultz, 2003; Schultz and Schmidt-Schultz, 2015) it was discussed on a clinical and pathophysiological basis the reason why GIs were supposed as a manifestation of TB meningitis.*”.

We thank you for your detailed and thoughtful comments. However, we must express our surprise regarding this critique, as it appears to question the diagnostic value of endocranial granular impressions in relation to clinically active tuberculosis disease (specifically tuberculous meningitis) by adopting the same arguments presented in **Genchi & colleagues (2025)**.

To briefly recall, as stated in the *Discussion and Conclusions* section of the original version of our manuscript (lines 367–376), we summarised the reasoning of Genchi & co-workers (2025) as follows:

“*The authors’ argument is based on that they found a quite high frequency of cases with GIs in a mediaeval (10^th^–12^th^-century-CE) osteoarchaeological series (Pieve di Pava, Italy) [128] that seems to be in conflict with modern disease prevalence data as TBM is a very rare extra-pulmonary form, occurring in less than 1% of all clinically active TB cases today [124,129–130]. Genchi & co-workers [128] state that their hypothesis is supported by (1) the short duration of TBM that is not long enough to allow bony changes (e.g., GIs) to develop on the endocranial surface and (2) the presence of cases with GIs not only among TB patients but also non-TB individuals from documented skeletal collections (Hamann-Todd Human Osteological Collection [94] and Robert J. Terry Anatomical Skeletal Collection [96,99]).*”.

These aforementioned arguments of Genchi & colleagues (2025) have already been explicitly addressed and refuted in the original version of our manuscript. Specifically, in the main text (lines 381–429), we presented three counterarguments against the hypothesis proposed by Genchi & co-workers (2025), which suggests that endocranial granular impressions represent merely signs of tuberculosis infection rather than clinically active tuberculosis disease (specifically tuberculous meningitis). These counterarguments were formulated in defence of the earlier, long-standing hypothesis proposed by Schultz (1999, 2001, 2003) and later strengthened by Schultz & Schmidt-Schultz (2015) and Spekker & colleagues (2020a, 2022), which interprets endocranial granular impressions as specific signs of clinically active tuberculosis disease (specifically tuberculous meningitis) rather than merely tuberculosis infection.

While we considered these three counterarguments already sufficient enough to demonstrate the weaknesses of Genchi & co-workers’ (2025) hypothesis and to support the validity of the earlier interpretation (Schultz, 1999, 2001, 2003; Schultz & Schmidt-Schultz, 2015; Spekker & colleagues, 2020a, 2022), five additional, more detailed counterarguments were presented in the *S1 Text*. In that file, we also provided an extended discussion of the three counterarguments already summarised in the main text.

Through our detailed counterarguments, we reaffirmed that the earlier, long-standing hypothesis (Schultz, 1999, 2001, 2003; Schultz & Schmidt-Schultz, 2015; Spekker & co-workers, 2020a, 2022) remains valid: the presence of endocranial granular impressions reflects clinically active tuberculosis disease (specifically tuberculous meningitis), rather than merely tuberculosis infection. As **Reviewer 4** explicitly noted, “*Special weight and importance of the work is given by Detailed refutation of Genchi & colleagues’ (2025) hypothesis that endocranial granular impressions are signs of tuberculosis infection rather than clinically active tuberculosis disease in Supporting Information where authors in eight points strengthen the previous hypothesis that GIs are pathognomonic features of TB(M) (rather than TB infection).*”.

Accordingly, your criticism seems to revisit issues that have already been carefully considered and addressed in the original version of our manuscript, rather than offering commentary on the counterarguments that we explicitly provided. Nonetheless, in the following section, we provide a point-by-point response to your individual statements, reiterating and clarifying our reasoning.

a) As for your comments “*However, GIs cannot be a manifestation of TB meningitis simply because this is a very rare complication of TB with a very rapid outcome, of which an individual would not live long enough for skeletal manifestations to develop.*” and “*Another point is that cases of protracted course of TB meningitis are reported in the modern clinical literature, but are absolutely exceptional; also, in this case, the authors cannot base their argumentation on rare exceptions, and again if TB meningitis is rapidly fatal today there are no reasons to suppose than in the past the course of this condition was longer enough to leave bone changes. Therefore, to support the hypothesis that GIs are a manifestation of TB meningitis is simply illogical.*”, we thank you for this observation. However, we would like to point out that the same argument, also presented by Genchi & colleagues (2025) – which appears to underlie both the authors’ and your assumption that endocranial granular impressions cannot represent signs of clinically active tuberculosis disease (specifically tuberculous meningitis), but only indicate tuberculosis infection – have already been discussed and refuted in detail in the original version of our manuscript, both in the main text and in the *S1 Text*.

In the main text (lines 396–414), briefly summarised:

“*By questioning the possibility of the development of endocranial bony changes (e.g., GIs) in TBM patients, Genchi & co-workers [128] also assume that the natural history and duration of TB(M) disease in the past (e.g., mediaeval period) were no different from its natural history and duration today. However, like the prevalence of TB(M), these aspects of the disease can be influenced by numerous factors [131-132]. As it has been highlighted by Spekker & colleagues [96] and Madai & co-workers (see Supplementary File 2 in [138]), in the modern medical literature, there are reports of cases (some come from the pre-antibiotic era, some from after the introduction of anti-TB chemotherapy with antibiotics) in which TBM had a protracted course (several months or even years) [e.g., 139-145]. At least in such cases (the prevalence of which is not known neither today nor in the past (e.g., mediaeval period)), the duration of the disease is long enough to allow skeletal lesions (e.g., GIs) to develop on the inner skull surface. On the other hand, the formation of GIs by pressure atrophy can require several weeks. Thus, even in cases with TBM where there is no initiation of antibiotic therapy and the disease do not have a protracted course but a typical one, the duration of TBM can be long enough to allow endocranial bony changes (e.g., GIs) to develop, especially in consideration that the period between the onset of the signs and symptoms of the disease and the death of the patient, which is preceded by the onset of the formation of tubercles on the meninges, is already several weeks or months (usually two to eight weeks) [146-148];*”.

In the *S1 text* (lines 143–193), discussed in more detail:

“*By questioning the possibility of the development of endocranial bony changes (e.g., GIs) in TBM patients, Genchi & colleagues [7] also assume that the natural history and duration of TB(M) disease in the past (e.g., mediaeval period) were no different from (or very similar to) its natural history and duration today. However, like the prevalence of TB(M), these aspects of the disease can be influenced by numerous factors [14-15].*

*As it has been highlighted by Spekker & co-workers [5] or Madai & colleagues (see Supplementary File 2 in [30]), in the modern medical literature, there are reports of cases (some come from the pre-antibiotic era, some from after the introduction of anti-TB chemotherapy with antibiotics) in which TBM had a protracted course (several months or even years) [e.g., 31-37]. At least in such cases (the prevalence of which is not known neither today nor in the past (e.g., mediaeval period)), the duration of the disease is long enough to allow skeletal lesions (e.g., GIs) to develop on the inner skull surface.*

*Furthermore, as it has been highlighted by Schultz [1-3] or Spekker & co-workers [5-6,38], GIs are the result of pressure atrophy – they match in their position to the tubercles of the dura mater encephali that can cause bone resorption (atrophy) by exploring pressure on the adjacent endocranial surface. For instance, in Schultz [1]: “However, there are also specific changes, which are only caused by a tuberculous process of the meninges. These characteristic lesions are small, roundish, and relatively flat impressions in the endocranial lamina of the skull and are caused by pressure atrophy of the tuberculars.”. Or in Spekker & colleagues [5]: “According to the results of Templin [28], Templin & Schultz [29], Schultz [24–26], and Schultz & Schmidt-Schultz [27], GIs may be established by pressure atrophy of the tubercles formed in the dura mater during later stages of TBM [24–29].”. Or in Spekker & co-workers [38]: “In later stages of the disease, not only the leptomeninges but also the outermost meningeal layer (i.e., the dura mater encephali) could have become affected by the pathological process [66, 84, 85]. As the dura mater encephali is directly adherent to the inner surface of the cranium, the gradually growing and coalescing meningeal tubercles could have exerted localised pressure on the underlying bone, inducing temporary, circumscribed bone atrophy and subsequent formation of endocranial GIs (Figs 2, 4, 5, 6A, 6B, 6D–6E, 15 and 17) [62, 65, 66, 69]. By definition, GIs are small (0.5–1.0 mm in diameter), relatively shallow (less than 0.5 mm in depth), roundish impressions with smooth margins and walls [62–66, 69]. They can appear separately or confluently and are usually grouped in clusters on the inner surface of the cranium [62–66, 69]. Granular impressions match in their position to those tubercles of the dura mater encephali, which cause bone resorption by exploring pressure on the underlying bone [62–66, 69].”.*

*Similar to GIs, abnormally pronounced digital impressions (APDIs) are the result of pressure atrophy – they match in their position to the cerebral gyri that can cause bone resorption (atrophy) by exploring pressure on the adjacent endocranial surface. In case of APDIs, this pathological process (i.e., the formation of APDIs by pressure atrophy) requires several weeks. For instance, in Spekker & colleagues [39]: “In adulthood, the presence of APDIs over the upper portion of the skull indicates a prolonged rise in the intracranial pressure: according to estimates, the formation of APDIs secondary to elevated intracranial pressure (eICP) requires at least ten weeks [29,30,32–36].”.*

*Based on the above, we can conclude that the formation of GIs by pressure atrophy (which are much smaller lesions than APDIs) can require several weeks, as well. Thus, even in cases with TBM where there is no initiation of anti-TB chemotherapy with antibiotics and the disease do not have a protracted course but a typical one, the duration of TBM can be long enough to allow skeletal lesions (e.g., GIs or APDIs) to develop, especially in consideration that the period between the onset of the signs and symptoms of the disease and the death of the patient, which is preceded by the onset of the formation of tubercles on the meninges, is already several weeks or months (usually two to eight weeks) [40-41];*”.

In summary, we can conclude that the duration of tuberculous meningitis can be sufficient to allow the formation of bony changes, such as endocranial granular impressions, not only in cases with a protracted disease course but also in those with a “typical” disease course. This is because the development of endocranial granular impressions is driven by pressure atrophy: during tuberculous meningitis, the tubercles that form on the *dura mater encephali* exert localised pressure on the adjacent endocranial surface, which leads to focal resorption of the cortical bone layers and the subsequent formation of granular impressions on the inner skull surface. This process takes several weeks, as evidenced by the formation of abnormally pronounced digital impressions – a similar pressure-atrophy-driven endocranial alteration type, which typically requires about 10 weeks to develop. Considering that, in untreated tuberculous meningitis, the interval between the onset of clinical signs and symptoms and death usually spans a few weeks (on average 2–8 weeks), and that the formation of meningeal tubercles necessarily precedes the appearance of clinical signs and symptoms, even patients with a “typical” disease course may have sufficient time for the development of endocranial granular impressions, even if not in every single case.

It is important to note that the argument questioning the association between endocranial granular impressions and tuberculous meningitis – specifically, the claim that these lesions could not form due to the allegedly too rapid course of the disease – raised both by you and by Genchi & colleagues (2025), has previously appeared in the literature (Roberts & co-workers, 2009) and has been refuted on multiple occasions (Spekker & colleagues, 2020a; Madai & co-workers, 2025) before the preparation of our current manuscript.

In addition to our above argument which we have elaborated in both the main text (lines 396–414) and the *S1 Text* (lines 143–193), another important line of evidence supporting the association between endocranial granular impressions and tuberculous meningitis is that they have a very specific localisation pattern on the inner surface of the skull. Although this argument was not included in the main text of the original version of our manuscript, it was discussed in detail in the *S1 Text* (lines 315–335):

“*The localisation pattern of GIs on the endocranial surface is not random as we would expect it if Genchi & co-workers’ [7] hypothesis would be correct and it would be the meningeal tubercles forming at the beginning of the TB infection after haematogenous dissemination of TB bacilli into the central nervous system that exert pressure on the inner skull surface for a long time; and thus, result in the development of GIs. As it has been presented by Schultz [1], Schultz & Schmidt-Schultz [4], and Spekker & colleagues [5], instead of being randomly scattered all over the endocranial surface, GIs are localised on the skull base and lower lateral skull vault.*

*During the pathogenesis of TBM, the formation of meningeal tubercles presents a very specific pattern (first the disease involves the meninges of the basal areas but in the end, it extends to the meninges covering the cerebral convexities). The localisation pattern of GIs on the endocranial surface follows this specific pattern of tubercle development during the pathogenesis of TBM (see Figure 1 in [5]): GIs are most frequently located on the endocranial surface of the squamous part of the occipital bone (adjacent to the cerebellum), the squamous part of the temporal bone (adjacent to the respective temporal lobe), and the orbital parts of the frontal bone (adjacent to the respective frontal lobes). Sometimes, the greater wings of the sphenoid bone (adjacent to the respective temporal lobes) and the parietal bones (close to the squamous suture) can also exhibit GIs. As it has been highlighted by Spekker & co-workers [5]: “The localization pattern and distribution of GIs on the endocranial surface resemble that of the tubercles observed in the affected meninges during the pathogenesis of TBM that further strengthens their tuberculous origin.”*.”

Since your comment only referred to the rapid course (and rarity) of tuberculous meningitis as an argument against the possibility that the disease can give rise to endocranial granular impressions, without acknowledging, refuting, or accepting our above counterargument regarding the characteristic localisation pattern of these lesions, we believe it is important to also include this counterargument in the main text of the revised version of our manuscript. Its inclusion there will help ensure that its significance is not overlooked, as seems to have occurred when it appeared only in the *S1 Text*. The slightly shortened version of the argument is as follows (lines 541–552 in the revised version of our manuscript):

“*The localisation pattern of GIs on the endocranial surface is not random as we would expect it if Genchi & co-workers’ [136] hypothesis would be correct and it would be the meningeal tubercles forming at the beginning of the TB infection after haematogenous dissemination of TB bacilli into the central nervous system that exert pressure on the inner skull surface for a long time; and thus, result in the development of GIs. As it has been presented by Schultz [99], Schultz & Schmidt-Schultz [103], and Spekker & colleagues [104], instead of being randomly scattered on the endocranial surface, GIs are localised on the skull base and lower lateral skull vault. During the pathogenesis of TBM, the formation of meningeal tubercles presents a very specific pattern (first the disease involves the meninges of the basal areas but in the end, it extends to the meninges covering the cerebral convexities). The localisation pattern of GIs on the endocranial surface follows this specific pattern of tubercle development during the pathogenesis of TBM (see Figure 1 in [104]).*”.

In summary, the localisation pattern of granular impressions on the inner skull surface is not random but corresponds closely to the characteristic distribution of meningeal tubercles during the course of tuberculous meningitis, thereby supporting their association with the disease.

Finally, concerning the first part of your comment (“*However, GIs cannot be a manifestation of TB meningitis simply because this is a very rare complication of TB*”), we do not see a direct causal link between the rarity of tuberculous meningitis and the formation of endocranial granular impressions. In other words, although tuberculous meningitis seems to be a very rare manifestation of tuberculosis nowadays, its rarity does not preclude the development of endocranial granular impressions in the affected individuals, as there are other diseases – for instance, those considered in the differential diagnosis of the clival lesions in MMG3-75 – that, despite being very rare, can still give rise to bony changes.

b) As for your comment “*In this case, the authors affirm that the lesions on the clivus developed following TB infection from the meninges; again, this is not possible as meningitis has a rapid clinical outcome, and the patient should have died before developing the erosion and lesions to the clivus, a process that requires a much longer time.*”, we would like to clarify that we do not understand the basis of this statement, which appears to be a misinterpretation. At no point of the original version of our manuscript did we claim that in MMG3-75, the clival lesions necessarily developed following the spread of tuberculosis from the meninges into the clivus.

What we did in the original version of our manuscript was to outline two possible pathogenetic scenarios in the *Discussion and Conclusions* section (lines 310–360) regarding how the observed mild cortical erosion and multiple, well-circumscribed osteolytic lesions at the clivus, as well as the granular impressions and abnormal blood vessel impressions in multiple locations on the endocranial surface, might have developed sequentially if they were indeed associated with tuberculosis in MMG3-75. In the first pathogenetic scenario (lines 310–340), we proposed that the disease might have spread from the clivus (i.e., tuberculous clival osteomyelitis) to the meninges (i.e., tuberculous meningitis):

“*The pathological skeletal lesions observed in the skull remains of MMG3-75 suggest that this younger adult male suffered from TB with concomitant involvement of the skull base (clivus) and the meninges. Although TB clival osteomyelitis usually results from contiguous spread of the TB infection from adjacent structures, predominantly the CVJ [21,59-60], there was no skeletal evidence of TB involvement of the atlanto-occipital joint, sphenoid sinus, or either petrous pyramids in MMG3-75, making this scenario less likely. Rather, the disease may have developed following haematogenous spread of TB bacilli from a primary focus elsewhere in the body of MMG3-75 [50,59] that unfortunately could not be identified during the palaeopathological examination of the skeleton. In MMG3-75, the presence of TB bacilli within the cancellous bone of the clivus may have triggered granulomatous inflammation with subsequent formation of the three larger, well-circumscribed osteolytic lesions observed within the upper, middle, and lower parts of the clivus (Figs 7a and 8) [29-30,33,35,39,41-44]. As the pathological process progressed, the TB infection may have spread from the cancellous bone to the cortical layers, resulting in perforations [29-30], with the consequent development of the multiple, very small, well-circumscribed osteolytic lesions observed in the upper-middle clivus of MMG3-75 (Figs 2–3). On the endocranial surface, the mild cortical erosion of the upper-middle clivus suggests the presence of an accumulation of TB granulation tissue at the sites of perforation (Figs 2–3 and 7c) [28,30,35,40,44,48-51].*

*In addition to the above, the presence of endocranial alterations indicative of TBM (GIs and ABVIs) in multiple locations on the inner skull surface of MMG3-75 (Figs 4–6) suggests that the TB infection may have extended from the clivus towards the meninges, at least the dura mater encephali. In the palaeopathological literature, endocranial GIs have been described as specific signs of TBM [91-93,95-96,99]. Their development is secondary to the pressure atrophy of the cortical bone layers, caused by the tubercles formed on the dura mater encephali as a result of its TB granulomatous inflammation [91,95-96,99]. Unlike GIs, endocranial ABVIs are not specific to TBM as they can be caused by many other aetiologies (e.g., bacterial meningitis, traumas, scurvy, brain tumours, and haemorrhages) [90,92-94,97,99,118-119]. In TBM, their formation is secondary to the healing of an epidural haematoma and associated vascularisation that develops following granulomatous inflammation and consequent disruption of at least some of the meningeal blood vessels and/or dural venous sinuses [90,92-93,97,99].*

In the second, alternative scenario (lines 341–360), we suggested that the disease could have spread in the opposite direction, from the meninges (i.e., tuberculous meningitis) to the clivus (i.e., tuberculous clival osteomyelitis):

“*An alternative scenario is that TB clival osteomyelitis may have developed following contiguous spread of the TB infection from the meninges towards the clivus of MMG3-75. In TBM, the primary event is the formation of a thick, gelatinous inflammatory exudate between the two layers of the leptomeninges (i.e., the pia mater encephali and the arachnoid mater encephali) [52,120-123]. The TB meningeal exudate is initially confined to the basal parts of the brain (the superior aspect of the cerebellum, the floor of the third ventricle, the anteromedial surface of the temporal lobes, and the inferomedial surface of the frontal lobes) but can rapidly spread to the basal cisterns (i.e., the interpeduncular and chiasmatic (suprasellar) cisterns, the former of which is adjacent to the upper-middle clivus) [52,120-122,124-125]. In later stages of the disease, the TB infection can spread to other cisterns (e.g., prepontine and ambient cisterns) [52,125-127]; finally, it can reach the ependymal surfaces of the ventricles, the choroid plexuses, and the cerebral convexities [52,120,124-125,127]. In MMG3-75, the TB meningeal exudate may have extended towards the interpeduncular cistern; its presence on top of the upper-middle clivus may have initiated bone resorption resulting in the mild cortical erosion observed (Figs 2–3 and 7c) [28,30,35,40,44,48-51]. Later, not only the cortical layers but also the cancellous bone of the clivus may have been affected by the TB infection; eventually, its granulomatous inflammation may have led to the development of the multiple, very small, well-circumscribed perforating osteolytic lesions in the upper-middle clivus (Figs 2–3) and the three larger, well-circumscribed osteolytic lesions within the upper, middle, and lower parts of the clivus of MMG3-75 (Figs 7a and 8) [29-30,33,35,39,41-44].*”.

**Reviewer 2** provided a positive assessment of the section on the possible pathogenetic scenarios, stating that “*2.) The description of the possible scenarios regarding pathogenesis (line 332–360) is detailed and well formulated. However, for readers with different scientific expertise it could be difficult to follow. I would suggest adding a schematic figure summarizing the process either with small pictures or boxes containing one- or two-word prompts for each step*.”. In response to this valuable suggestion, the revised version of our manuscript now includes two supplementary figures (*S2 Figure* and *S3 Figure*) that illustrate these two pathogenetic scenarios*.*

It should also be emphasised that we never took a position on which of these two scenarios is more likely, as this cannot be determined based on the available data. This limitation is explicitly noted in the newly created *Conclusions* section (lines 739–745), which was separated from the *Discussion* in the revised version of our manuscript in response to **Reviewer 3**’s suggestion, where we discuss the diagnostic challenges in establishing tuberculous clival osteomyelitis as a primary diagnosis in MMG3-75:

“*It should be noted that although we were able to outline these potential pathogenetic scenarios, illustrating how the cranial bony changes could have developed if our hypothesis is correct (i.e., if TB was indeed the underlying cause in MMG3-75), unfortunately, we could not clarify which of these scenarios took place in MMG3-75, nor could we establish the primary focus of the TB infection within this younger adult male’s body (from where the TB bacilli presumably first reached either the clivus (S2 Figure) or the meninges (S3 Figure) via haematogenous spread).*”.

Among the co-authors of our manuscript, five hold medical degrees (András Palkó, Ádám Perényi, Árpád Szabó, Tímea Katalin Mai, and Olivier Dutour), and their opinions differ regarding which pathogenetic scenario was more likely in MMG3-75. For this reason alone, we could not take the position that you have attributed to us, namely that we chose the second scenario (where the disease spread from the meninges to the clivus).

Furthermore, we would also like to note that the second part of your statement (“*again, this is not possible as meningitis has a rapid clinical outcome, and the patient should have died before developing the erosion and lesions to the clivus, a process that requires a much longer time*”) is also inaccurate. Modern medical literature reported that, although rarely, tuberculosis can indeed spread from the meninges to the cranial bones, leading to tuberculous calvarial (Park & co-workers, 2024) or skull-base (Kolbinger & colleagues, 1994; Vaid & co-workers, 2010) osteomyelitis. This supports our position that, contrary to your assertion, the development of bony changes in the context of tuberculous meningitis is not impossible, and it is likewise not inconceivable that tuberculosis can spread from the meninges to the cranial bones (both calvaria and skull base).

In Park & co-workers (2024):

“*Although tuberculosis can affect various parts of the central nervous system, tuberculous meningitis is the most lethal form. Tuberculous meningoencephalitis and cranial tuberculosis are extremely rare. Therefore, the authors present an autopsy case of tuberculous meningoencephalitis resulting in skull perforation.*” (English abstract).

In Kolbinger & colleagues (1994):

“*A 34-year-old Vietnamese patient presented with tuberculous meningoencephalitis complicated by hydrocephalus, diabetes insipidus, and radiological evidence of skull base erosion. Despite antituberculous treatment and corticosteroids multiple basal tuberculomas developed.*” (English abstract).

In Vaid & co-workers (2010):

“*The skull base and CV junction are secondarily involved by direct extension of disease from the cranial vault, paranasal sinus, or mastoid–middle ear complex, and rarely due to meningitis.*” (page 76).

It is important to note that, from a diagnostic perspective, it is of secondary importance which of the two pathogenetic scenarios was more likely or which one actually occurred in MMG3-75. Both pathways are theoretically possible, and our purpose in outlining these scenarios was not to determine the precise chronological order in which the cranial bony changes observed in this younger adult male developed, but rather to demonstrate that, if our working hypothesis is correct (i.e., tuberculosis was indeed the underlying cause in MMG3-75), there are plausible pathogenetic pathways – in fact, more than one – that could account for the sequential development of both the clival lesions (mild cortical erosion and multiple, well-circumscribed osteolytic lesions) and the endocranial alterations (granular impressions and abnormal blood vessel impressions).

c) As for your comment “*In the paper of Genchi et al. (2025), GIs were found in half of the individuals examined from the Medieval population of Pava, and this strongly demonstrates that they are pathognomonic of TB infection and not TB meningitis; in fact, it is unreasonable to hypothesize that half of the population died from this very rare complication of the central nervous system. Considering that today the prevalence of TB meningitis is 1-5%, and that in the Medieval population of Pava GIs affected half of the skeletal assemblage, even admitting that in the past the prevalence of TB meningitis could have been higher than today, it is absolutely unlikely that the disease reached a prevalence of 50%. The pathophysiology of the human body cannot be changed during a few hundred years!*”, thank you for this observation and for referring to the study by Genchi & colleagues (2025). However, we would like to note that your statement reflects the same misunderstanding/misinterpretation that appears in Genchi & co-workers’ (2025) paper itself. As we already pointed out in the main text (lines 381–395) and in the *S1 Text* (lines 19–142) of the original version of our manuscript, the conclusions drawn by Genchi & colleagues (2025) regarding endocranial granular impressions are not supported by the available palaeopathological or clinical evidence. We have already discussed these issues and presented multiple counterarguments in detail in the cited sections.

In the *S1 Text* (lines 19–110) we elaborated in detail on two counterarguments that were not addressed in the main text of the original version of our manuscript:

“*Genchi & co-workers [7] calculated only the frequency of cases with GIs (and based on this, the number of diagnosed cases with clinically active TBM disease/frequency of TBM), not the prevalence of TBM, in the examined osteoarchaeological series from mediaeval Pieve di Pava. Then, the authors compared the calculated frequency of cases with GIs/frequency of TBM to modern disease prevalence data and assumed that if GIs would be pathognomonic features of clinically active TB(M) disease, the prevalence of TBM would be too high in the once-lived population of Pieve di Pava (compared to a prevalence of less than 1% of all clinically active TB cases today). However, only the prevalence of TBM in mediaeval Pieve di Pava may be appropriate to be compared to modern disease prevalence data. As it has been highlighted by Milner & Boldsen [12], estimating the prevalence of a disease (e.g., TBM) in osteoarchaeological series is no easy task: “Estimating disease prevalence from archaeological bones and teeth is no easy task (Boldsen and Milner, 2012; DeWitte and Stojanowski, 2015; Ortner, 1991; Wood et al., 1992; Wright and Yoder, 2003).”. It is much more complicated than considering only the frequency of a lesion type (e.g., GIs) or the number of diagnosed cases (e.g., number of diagnosed cases with clinically active TB(M) disease) in the examined sample [12];*” (lines 19–34); and

“*Only disease prevalence data deriving from a representative sample may be appropriate to be used for comparing to modern disease prevalence data but at the current state of research, it is not possible to properly estimate the prevalence of TBM in the once-lived population of Pieve di Pava.*

*On the one hand, the sample examined by Genchi & colleagues [7] does not seem to be representative of mediaeval Pieve di Pava. First, from the more than 900 excavated individuals, only 383 skeletons were subjected to an anthropological analysis, and only 212 were evaluated for the presence of GIs on the inner skull surface, which is less than one-fourth of the excavated individuals. Second, as Milner & Boldsen [12] argues: “Perhaps it is best to start with a misconception: we are primarily concerned with skeletons. They are indeed what are observed, but they are most assuredly not what we wish to know about. When estimating the prevalence of a pathological condition in the past, it is the once-living people who are of concern. They are the people who would be examined, measured, and described if it were possible to be magically transported back in time. The skeletons we look at, in contrast, are from the unfortunates who failed to survive to the next age interval. They are, in effect, life’s failures each step of the way from infancy to old age.”; “Identifying the people at risk from whatever is of interest is critical because the population represented by skeletons has a major effect on the research questions that can be asked of the data at hand. Not just any group of skeletons can be considered to represent a population for analytical purposes. Skeletons, of course, are a sample of the population of interest, even if they are from a completely excavated cemetery. That does not necessarily present a problem because in many fields small samples are routinely used to say something useful about much larger populations. The difficulty when examining skeletal remains lies in ascertaining the representativeness of the sample, a problem commonly overlooked by tacit agreement. Sometimes it is even confused with the size of the sample, such as when many skeletons, without further consideration, are reflexively thought to ensure better results.”; “One reason it might be a good idea to avoid the word population when referring to excavated human remains pertains to the very nature of archaeological materials. Skeletons are necessarily a biased sample of the people who were once alive, hence the population from which the bones were derived. As pointed out here and elsewhere (e.g., Wood et al., 1992), what comes to a bioarchaeologist’s attention are people sampled at the moment of their death. They are assuredly not the full range of people who would be observed if one could actually survey a past living population. To once again state the obvious, one cannot avoid sampling dead people when examining skeletons. For bioarchaeological purposes, therefore, the ideal sample is one that is biased only by the fact that the individuals examined are those who were removed by death from the population at each age.”. “In some instances, it is possible for researchers to identify the limits of the group being examined, hence have something approximating a population. The matter is simplified when the past community associated with the cemetery was a reasonably stable group of people over time. Examples might include cemeteries associated with villages of subsistence agriculturalists or medieval peasants that tended to be spatially discrete communities, were characterized by relatively little internal socioeconomic differentiation, and were often associated with single burial areas.”; and “It follows that all skeletal assemblages are not equally useful for paleoepidemiological purposes. Questionable examples include skeletons from burial grounds associated with monasteries, hospitals, and shrines that attracted people from distant places (Inskip et al., 2015; Müldner et al., 2009; Roffey et al., 2017; Torino et al., 2015). Even when those institutions mostly drew from local communities, the skeletons might not be representative of the people who inhabited those places. Medieval hospitals, for example, also doubled as almshouses (Harvey and Oeppen, 2001). In such situations, poor people could be overrepresented relative to the surrounding population.”. Based on the above, the sample examined by Genchi & co-workers [7] cannot be considered representative of the once-lived population of Pieve di Pava. For instance, the authors state in the Archaeological background section that 1) subadults are underrepresented (“Most individuals excavated were adults, while infants and young children were largely underrepresented (Riccomi, 2021); it is likely that they were buried in a distinct funerary area that has not yet been archeologically identified (Felici, 2016).”) and 2) the cemetery is associated with a religious building (“The area surrounding the religious building was intensively used between the 10th and 12th centuries, as documented by the large number of west-east orientated burials (Felici, 2016). Although archeological investigations have not been able to identify the settlement associated with the religious building, several anthropological, paleopathological, and biochemical studies revealed interesting aspects of the living conditions, health, and diet of a substantial number of individuals from this site (Ricci et al., 2012; Fornaciari, 2016; Riccomi, 2021; Riccomi et al., 2021; Kinkopf et al., 2022; Monaco et al., 2022).”). Based on the above arguments of Milner & Boldsen [12], these two aspects already make the representativeness of the examined sample from mediaeval Pieve di Pava highly questionable.*

*On the other hand, right now, it is impossible to properly estimate the prevalence of clinically active TB(M) disease in osteoarchaeological series as the existing palaeoepidemiological methods are not yet appropriate for that. Hopefully, in the future, it will become possible – there are already some promising studies [e.g., 13] that try to assess the prevalence of clinically active TB disease in osteoarchaeological series. Nonetheless, even if such palaeoepidemiological methods will be available in the future, the sample examined by Genchi & colleagues [7] (at least in its present form) would not be appropriate for these kind of analyses as it is not representative of the once-lived population of Pieve di Pava;”* (lines 35–110).

For clarity, we briefly summarise these two counterarguments here:

(1) Frequency vs. prevalence:

Genchi & colleagues (2025) calculated only the frequency of cases with endocranial granular impressions in the examined skeletal assemblage from mediaeval Pieve di Pava. Importantly, they inspected the endocranial surfaces of only about 200 skeletons, whereas the entire osteoarchaeological series comprises more than 900 individuals; therefore, the reported lesion frequency (i.e., the number of cases with endocranial granular impressions) was obtained from a limited subset of the skeletal assemblage from mediaeval Pieve di Pava. From this limited subset (~200 skeletons), the authors inferred disease prevalence (i.e., the prevalence of tuberculous meningitis in the once-lived population of mediaeval Pieve di Pava) and compared it to modern disease prevalence data (i.e., the prevalence of tuberculous meningitis today); essentially treating the observed lesion frequency as equivalent to disease prevalence.

However, lesion frequency cannot be equated with disease prevalence (Milner & Boldsen, 2017), and it is thus clear that comparing the lesion frequency (i.e., the number of cases with endocranial granular impressions) from the examined subset of the skeletal assemblage of mediaeval Pieve di Pava to the modern prevalence of tuberculous meningitis is unfounded and misleading. The appropriate approach would have been to compare the estimated prevalence of tuberculous meningitis in the once-lived population of mediaeval Pieve di Pava with the modern prevalence data of tuberculous meningitis; and

(2) Methodological and representational limitations:

Unfortunately, Genchi & co-workers (2025) cannot fulfil the approach outlined in point (1), as reliable estimation of the prevalence of tuberculous meningitis in the once-lived population of mediaeval Pieve di Pava is currently not possible. There are two main reasons for this:

(a) Although there are some promising attempts, currently no validated palaeoepidemiological methods exist that would allow reliable estimation of disease prevalence – not only for tuberculous meningitis, but for any disease – in human osteoarchaeological series; and
(b) The subset examined by Genchi & colleagues (2025) is not representative of the once-lived population of Pieve di Pava. Importantly, not only is the subset analysed by Genchi & co-workers (2025) unrepresentative, but even the entire skeletal assemblage from mediaeval Pieve di Pava cannot be considered representative of the once-lived population (e.g., children under the age of 2 years are missing; and the cemetery is associated with a religious building) (Milner & Boldsen, 2017).

Therefore, even if validated palaeoepidemiological methods existed that could reliably estimate the prevalence of tuberculous meningitis in the examined subset or in the complete osteoarchaeological series, the lack of representativeness means that such estimates would not provide any meaningful information about the actual prevalence of the disease in the once-lived population of mediaeval Pieve di Pava.

Based on the above, it is therefore clear that the lesion frequency (i.e., the number of cases with endocranial granular impressions) reported by Genchi & colleagues (2025) from the subset of the skeletal assemblage of mediaeval Pieve di Pava provides no reliable information on the actual prevalence of tuberculous meningitis in the once-lived population, and comparing it to modern disease prevalence data (i.e., the prevalence of tuberculous meningitis today) can easily lead to misinterpretation, as may have inadvertently occurred in the case of both Genchi & co-workers (2025) and you.

For instance, you state that “*In the paper of Genchi et al. (2025), GIs were found in half of the individuals examined from the Medieval population of Pava, and this strongly demonstrates that they are pathognomonic of TB infection and not TB meningitis*”. This conclusion is not appropriate. Genchi & colleagues (2025) did not study the once-lived population of mediaeval Pieve di Pava, but rather an osteoarchaeological series, and only a subset of it, which is not representative of the once-lived population (Milner & Boldsen, 2017). Therefore, the lesion frequency (i.e., the number of cases with endocranial granular impressions) in this subset provides no meaningful information on how many individuals in the once-lived population actually had endocranial granular impressions, nor on the actual prevalence of tuberculous meningitis in that population. In other words, we cannot claim that around 50% of the once-lived population of mediaeval Pieve di Pava had endocranial granular impressions, and it is even less justifiable to suggest that the prevalence of tuberculous meningitis in this population was around 50%.

You also state that “*in fact, it is unreasonable to hypothesize that half of the population died from this very rare complication of the central nervous system. Considering that today the prevalence of TB meningitis is 1-5%, and that in the Medieval population of Pava GIs affected half of the skeletal assemblage, even admitting that in the past the prevalence of TB meningitis could have been higher than today, it is absolutely unlikely that the disease reached a prevalence of 50%.*”. We fully agree with you that it is entirely unreasonable to conclude that half of the once-lived population of mediaeval Pieve di Pava died from tuberculous meningitis, solely based on the finding of Genchi & co-workers (2025) that endocranial granular impressions were observed in approximately half of the cases within the examined subset. However, our underlying rationale is fundamentally different. The presence of endocranial granular impressions, indicative of tuberculous meningitis, does not necessarily imply that the affected individual died from this disease. Even if the disease had developed, the individual could have died from another cause (e.g., cardiac arrest, accident or homicide) before tuberculous meningitis could become fatal. Therefore, even if the lesion frequency (i.e., the number of cases with endocranial granular impressions) in the subset examined by Genchi & colleagues (2025) was around 50% (bearing in mind that this subset is not representative of the once-lived population of mediaeval Pieve di Pava), it in no way suggests that half of the once-lived population actually died from tuberculous meningitis. Moreover, as we mentioned above, this lesion frequency provides no meaningful information on the prevalence of tuberculous meningitis in this population and certainly does not indicate that it was approximately 50%. In summary, it is indeed entirely unreasonable to claim, based solely on the lesion (i.e., endocranial granular impressions) frequency calculated by Genchi & co-workers (2025), that the prevalence of tuberculous meningitis could have been 50%, or that half of the once-lived population of mediaeval Pieve di Pava died from this disease.

When discussing MMG3-75 in our current manuscript, we were particularly cautious not to claim that this younger adult male died from tuberculous clivus osteomyelitis and associated meningitis. Instead, we stated that he suffered from the disease at the time of death. This distinction was crucial because other circumstances, such as falling during the main engagement of the Battle of Mohács, defending the camp, attempting to flee, or even being executed, could have caused his death before tuberculosis itself could have been fatal.

It should be noted that the two counterarguments we discussed in detail above were previously presented only in the *S1 Text* in the original version of our manuscript (lines 19–110); as a result, their significance may not have been fully appreciated during the review process, since they were neither explicitly acknowledged, refuted, nor accepted. To emphasise their importance, we have therefore included a concise, summarised version in the main text of the revised version of our manuscript (lines 447–464):

“*Genchi & colleagues [136] calculated only the frequency of cases with GIs (and based on this, the frequency of TBM), not the prevalence of TBM, in the examined osteoarchaeological series from mediaeval Pieve di Pava. Then, they compared the estimated frequency of TBM to modern disease prevalence data. However, only the prevalence of TBM in mediaeval Pieve di Pava may be appropriate to be compared to modern disease prevalence data. As it was highlighted by Milner & Boldsen [139], estimating the prevalence of a disease (e.g., TBM) in osteoarchaeological series is no easy task, and is much more complicated than considering only the frequency of a lesion type (e.g., GIs) or the number of diagnosed cases (e.g., number of diagnosed TB(M) cases) in the examined sample;*”; and

“*Only disease prevalence data deriving from a representative sample may be appropriate to be used for comparing to modern disease prevalence data but at the current state of research, it is not possible to properly estimate the prevalence of TBM in the once-lived population of mediaeval Pieve di Pava: a) the sample examined by Genchi & co-workers [136] does not seem to be representative of the once-lived population of mediaeval Pieve di Pava (e.g., children under the age of 2 years are missing; and the cemetery is associated with a religious building) [139] and b) although there are some promising palaeoepidemiological studies [e.g., 112] that try to assess the prevalence of TB disease in osteoarchaeological series, further research is needed to develop better methods;*”.

As for your comment “*Considering that today the prevalence of TB meningitis is 1-5%, and that in the Medieval population of Pava GIs affected half of the skeletal assemblage, even admitting that in the past the prevalence of TB meningitis could have been higher than today, it is absolutely unlikely that the disease reached a prevalence of 50%.*”, we would like to reiterate that the assumption made by Genchi & colleagues (2025) – namely, that the prevalence of tuberculous meningitis in the once-lived population of mediaeval Pieve di Pava was around 50% – is entirely unfounded. This conclusion was drawn solely from the observation that the frequency of cases with endocranial granular impressions was approximately 50% in a subset of the skeletal assemblage, which is not representative of the once-lived population.

Another important issue is that it appears that not only Genchi & co-workers (2025), but also you, have made assumptions about the actual prevalence of tuberculous meningitis in the once-lived population of mediaeval Pieve di Pava – more specifically, about how high it could not have been (i.e., that it could not have reached 50%). The problem with this line of reasoning is that, at present, we have no reliable data on what the prevalence of tuberculous meningitis was in the past or how it changed over time. To gain such insights, we would first need validated palaeoepidemiological methods that could accurately estimate disease prevalence in human osteoarchaeological series, thereby enabling meaningful comparisons between archaeological periods and geographic regions. Only such approaches would allow us to understand the past prevalence of tuberculous meningitis and its temporal and spatial variations, as well as the factors that might have influenced them.

It should also be noted that starting from the modern prevalence of the disease (i.e., tuberculous meningitis) and drawing conclusions about what its prevalence might or might not have been in the past is not a sound approach for several reasons. We addressed this issue in one of our other counterarguments, which was discussed both in the main text and in the *S1 Text* of the original version of our manuscript.

In the main text (lines 381–395), briefly summarised:

“*By comparing the frequency of cases with GIs (and based on this, the frequency of TBM) in mediaeval Pieve di Pava to modern disease prevalence data and then, stating that GIs cannot be pathognomonic features of clinically active TB(M) disease (as otherwise the prevalence of TBM would be too high in the once-lived population of Pieve di Pava compared to a prevalence of less than 1% of clinically active TB cases today), Genchi & colleagues [128] assume that the prevalence of TB(M) in the past (e.g., mediaeval period) was no different from (or very similar to) its prevalence today. However, the prevalence of TB(M) can be influenced by numerous factors (e.g., nutritional status, age, sex, and genetic factors of the host, and/or genetic factors of the pathogen) [131-132]. It has already been reported in some palaeopathological papers [e.g., 133-135] that before the introduction of the BCG vaccine and anti-TB chemotherapy with antibiotics, the prevalence of TB and its forms (e.g., TBM), and the frequency of some TB-associated bony changes were different from today’s trends. On the other hand, our understanding of the global burden of TBM today is limited but it is likely that numerous cases are not diagnosed; thus, the prevalence of TBM, especially in high-TB-burden countries, may be much higher than previously thought [136-137];*”.

In the *S1 Text* (lines 111–142), discussed in detail:

“*By comparing the calculated frequency of cases with GIs/frequency of TBM in mediaeval Pieve di Pava to modern disease prevalence data and then, stating that GIs cannot be pathognomonic features of clinically active TB(M) disease (as otherwise the prevalence of TBM would be too high in the once-lived population of Pieve di Pava compared to a prevalence of less than 1% of all clinically active TB cases today), Genchi & co-workers [7] assume that the prevalence of TB(M) in the past (e.g., mediaeval period) was no different from (or very similar to) its prevalence today. However, the prevalence of TB(M) can be influenced by numerous factors (e.g., nutritional status, age, sex, and genetic factors of the host, and/or genetic factors of the pathogen) [14-15].*

*Based on modern disease prevalence data, TB is a very rare disease in developed countries today [16]. If the authors’ above assumption would be correct, this would mean that TB was a very rare disease also in the past, but this is not true as we know that for instance, in the 19^th^ century and at the beginning of the 20^th^ century, the prevalence of TB disease was much higher [17-18]. We also know that for instance, after the introduction of the BCG vaccine, the prevalence of TBM substantially decreased as the BCG vaccination has a significant protective effect against the disease [19-20]. Despite this, the reported prevalence of TBM (e.g., ~7–12% of all cases with clinically active TB disease in [21]) was higher a couple of decades ago than today (e.g., ~1% of all cases with clinically active TB disease in [22]). Furthermore, recent studies [23-24] showed that because of a decrease in BCG immunization coverage or BCG vaccine supply shortages, the number of paediatric cases with TBM significantly increased in Bosnia and Herzegovina, and South Africa, respectively. Therefore, it is not correct if the authors assume that the prevalence of TBM in the past was no different from (or very similar to) its prevalence today. Currently, we do not know anything about how the prevalence of clinically active TB(M) disease has changed over time as we cannot properly estimate the prevalence of TB(M) in past human populations (e.g., mediaeval Pieve di Pava). Nevertheless, it has been reported in some palaeopathological papers [e.g., 25-27] that before the introduction of the BCG vaccine and anti-TB chemotherapy with antibiotics, the prevalence of TB and its forms (e.g., TBM), and the frequency of some TB-associated bony changes were different from today’s trends.*

*Finally, our understanding of the global burden of TBM today is limited but it is likely that numerous cases are not diagnosed; thus, the prevalence of TBM, especially in high-TB-burden countries, may be much higher than previously thought [28-29];*” (lines 111–142).

In summary, in both the main text (lines 381–395) and the *S1 Text* (lines 111–142) of the original version of our manuscript, we emphasised that it is methodologically unsound to assume that the prevalence of tuberculous meningitis in the past was the same as, or similar to, its prevalence today. The prevalence of tuberculosis and its manifestations, including tuberculous meningitis, is influenced by numerous host-, pathogen-, and environment-related factors, all of which have changed substantially over time. Historical and palaeopathological evidence clearly demonstrates that before the introduction of the BCG vaccine and anti-tuberculosis antibiotics, tuberculosis and its forms, including tuberculous meningitis, were far more common than they are today. Moreover, even the current global prevalence of tuberculous meningitis is likely underestimated due to diagnostic challenges and underreporting, particularly in high-burden regions. Therefore, comparisons between lesion frequencies in human osteoarchaeological series and modern disease prevalence data are inherently unreliable and can easily lead to erroneous conclusions about the actual prevalence of tuberculous meningitis in past human populations.

Finally, we would like to emphasise that the lesion frequency reported by Genchi & colleagues (2025) is not only unsuitable for estimating the prevalence of tuberculous meningitis in the once-lived population of mediaeval Pieve di Pava, but is also entirely inappropriate for drawing conclusions about the diagnostic efficacy of a lesion – namely, whether the presence of endocranial granular impressions indicates clinically active tuberculosis disease (specifically tuberculous meningitis) or merely tuberculosis infection.

To properly assess the diagnostic efficacy of a lesion – that is, to determine whether it can be reliably used as a diagnostic criterion for a given disease (e.g., whether endocranial granular impressions can serve as a diagnostic criterion for tuberculous meningitis) – one must rely on a study sample in which it is known which individuals suffered from tuberculosis and which did not. This requires either a pre-antibiotic-era documented human skeletal collection (e.g., Hamann-Todd Human Osteological Collection, Robert J. Terry Anatomical Skeletal Collection, and Coimbra Identified Skeletal Collection), where causes of death are reliably recorded in death certificates or morgue records, or a human osteoarchaeological series in which independent confirmation of tuberculosis diagnosis is provided by biomolecular methods (e.g., aDNA- or lipid biomarker-based analyses) (Roberts & co-workers, 2009). Unfortunately, the skeletal assemblage from mediaeval Pieve di Pava does not fall into either of these categories.

It is important to note, as further elaborated in response to a later comment from you, that palaeomicrobiological methods do not always provide reliable results: a negative outcome does not prove that an individual was disease-free, but merely that the pathogen could not be detected. While positive results can identify some affected individuals, others remain hidden due to false negatives and are indistinguishable from those who were truly disease-free. For this reason, human osteoarchaeological series cannot reliably inform the diagnostic efficacy of a lesion; only documented human skeletal collections, with causes of death confirmed by death certificates or morgue records, can provide an appropriate basis for such an evaluation.

Nevertheless, in support of their own hypothesis, Genchi & colleagues (2025) criticised studies conducted on pre-antibiotic-era documented human skeletal collections (Hershkovitz & co-workers, 2002; Spekker & colleagues, 2020a, 2022) that aimed to evaluate the diagnostic efficacy of endocranial granular impressions for tuberculous meningitis. Their main argument, supporting their own hypothesis (i.e., that endocranial granular impressions are only signs of tuberculosis infection rather than clinically active tuberculosis disease, specifically tuberculous meningitis) and opposing the earlier, long-standing hypothesis (i.e., that endocranial granular impressions are signs of clinically active tuberculosis disease (specifically tuberculous meningitis) rather than only tuberculosis infection), was that in these studies, conducted on the Hamann-Todd Human Osteological Collection (Hershkovitz & co-workers, 2002) and the Robert J. Terry Anatomical Skeletal Collection (Spekker & colleagues, 2020a, 2022), endocranial granular impressions were observed not only in individuals whose recorded cause of death was tuberculosis, but in some cases also in those who had died from other causes according to death certificates or morgue records.

In the original version of our manuscript, we also presented counterarguments regarding this issue: three in total, of which two were included only in the *S1 Text* (lines 194–314), while one was incorporated in the main text (lines 415–429) as well.

In the main text (lines 415–429), briefly summarised:

“*By stating that the presence of cases with GIs not only among TB patients but also non-TB individuals from documented skeletal collections supports that GIs are signs of TB infection rather than clinically active TB(M) disease, Genchi & colleagues [128] seem to overlook that in the two documented skeletal collections they mentioned (Hamann-Todd Human Osteological Collection [94] and Robert J. Terry Anatomical Skeletal Collection [96,99]), individuals were divided into two groups based on what they died of – TB group (those who died of TB) and non-TB group (those who died of non-TB causes) – as there is information about their recorded cause of death only. We do not know the complete medical history of these individuals; thus, we do not know if someone who died of a non-TB cause (e.g., accident, cancer or cardiovascular disease) and was placed into the non-TB group had clinically active TB(M) disease or not (i.e., was a TB(M) patient or not) at the time of death. Based on the above, in the non-TB groups of the aforementioned documented skeletal collections, there may be at least a few TB(M) patients [94,104] and this is why it is not surprising that some of the individuals who died of non-TB causes presented GIs, indicating that they suffered from clinically active TB(M) disease.*”.

In the *S1 Text* (lines 194–314), discussed in detail:

“*By stating that the presence of cases with GIs not only among TB patients but also non-TB individuals from documented skeletal collections (Hamann-Todd Human Osteological Collection [11] and Robert J. Terry Anatomical Skeletal Collection [5-6]) supports that GIs are signs of TB infection rather than clinically active TB(M) disease, Genchi & co-workers [7] seem to overlook that in the two aforementioned documented skeletal collections, individuals were divided into two groups based on what they died of – TB group (those who died of TB) and non-TB group (those who died of non-TB causes) – as there is information about their recorded cause of death only. We do not know the complete medical history of these individuals; thus, even if some of them died of a non-TB cause (e.g., accident, cancer or cardiovascular disease), it does not mean that they did not suffer from clinically active TB(M) disease (with presenting signs and symptoms) at the time of death or that they were not diagnosed with TB(M) disease before they died. The only thing we know is what they died of – TB or a non-TB cause. Based on the above, we do not know if someone who died of a non-TB cause; and thus, was placed into the non-TB groups of the above-mentioned two documented skeletal collections had clinically active TB(M) disease or not (i.e., was a TB(M) patient or not) at the time of death. Consequently, in the non-TB groups of the Hamann-Todd Human Osteological Collection and the Robert J. Terry Anatomical Skeletal Collection, there may be at least a few TB(M) patients [11,13] and this is why it is not surprising that some of the individuals who died of non-TB causes presented GIs, indicating that they suffered from clinically active TB(M) disease (even though TB(M) was not their recorded cause of death).*

*Not to mention that there can be cases in which an inaccurate cause of death was recorded. As it has been highlighted by Spekker & colleagues [5] regarding the Robert J. Terry Anatomical Skeletal Collection: “It must be noted that even if the recorded cause of death of individuals surveyed in the Terry Collection may not have been TB, individuals could still have suffered from the disease but their death was attributed to another medical condition [18–19]. Moreover, there is always the possibility that an inaccurate cause of death was registered on the morgue record and/or death certificate of individuals from the Terry Collection. Thus, it is possible that in the aforementioned six cases, the observed endocranial and non-endocranial bony changes resulted from TB. In summary, the findings of our study confirm those of Schultz [e.g., 24–26] and Schultz & Schmidt-Schultz [27] that GIs can be considered as pathognomonic features of TBM; and therefore, the paleopathological diagnosis of TBM can be established with a high certainty when GIs are present in ancient human bone remains. The localization pattern and distribution of GIs on the endocranial surface resemble that of the tubercles observed in the affected meninges during the pathogenesis of TBM that further strengthens their tuberculous origin.”. Santos [42] has also mentioned regarding another pre-antibiotic-era documented skeletal collection, the Coimbra Identified Skeletal Collection (individuals of the collection died during the first half of the 20^th^ century CE), that “Medical knowledge at that time suggests many cases of tuberculosis were misdiagnosed and diseases diagnosed as enteritis in children, typhoid in children and teenagers, pertussis, influenza, meningitis and pulmonary diseases, such as pleurisy and pneumonia (Carvalho, 1907), and also bronchitis (Carvalho, 1907; Porto, 1927) and emphysema, very frequent in elderly people (Porto, 1927; Lopo de Carvalho, 1935), were in fact tuberculosis. Moreover, tuberculosis in different organs of the body, such as liver, kidneys, suprarenal glands and genitals, among others, was very often not recognised (Carvalho, 1907).”, which further strengthens the above argument that some of the individuals who died of a non-TB cause; and thus, were placed into the non-TB groups of the aforementioned documented skeletal collections, could have suffered from clinically active TB(M) disease (i.e., could have been TB(M) patients) at the time of death (even though TB(M) was not their recorded cause of death).*

*Furthermore, as it has been presented by Spekker & co-workers [5], in the Robert J. Terry Anatomical Skeletal Collection, from the 193 individuals in the non-TB group, only six showed GIs (6/193; ~3%). Based on the above, there is a possibility that an inaccurate cause of death was recorded in at least some of these six cases but even if the recorded cause of death is accurate, these individuals are very likely to have been TB patients who died of non-TB causes. In the same paper by Spekker & colleagues [5], the S2 Table (“Basic biographic data of individuals in the NTB group (N=193)”) contains detailed information (e.g., age at death, sex, and cause of death based on the morgue record (MR) and/or death certificate (DC)) about the individuals in the non-TB group. Based on these data, the recorded cause of death of the aforementioned six individuals were the following: 1) Terry No. 4R died of antral carcinoma (MR)/metastasis of the left orbit (DC); 2) Terry No. 197R died of breast carcinoma (MR); 3) Terry No. 272 died of cardiac decompensation, nephritis (MR); 4) Terry No. 465 died of post-operative wound of the chest (MR)/aortic aneurysm (DC); 5) Terry No. 506 died of myocarditis (MR)/asthma, myocarditis (DC); and 6) Terry No. 1378 died of peritonitis (MR). As it can be seen, none of these non-TB causes of death can be responsible for the development of GIs on the inner skull surface, which also supports that the aforementioned six individuals were very likely to have suffered from TB(M) (i.e., were TB patients) but died of a non-TB cause (e.g., cancer or peritonitis) that was concurrently present with TB;*” (lines 194–262);

“*The Hamann-Todd Human Osteological Collection and the Robert J. Terry Anatomical Skeletal Collection were established when and where many people, possibly everyone, were exposed to TB bacilli [11,13,43]; thus, individuals from the above-mentioned documented skeletal collections are likely to had TB infection – in the TB group, they were not only infected by TB but the infection progressed into clinically active TB disease in all cases, whereas in the non-TB group, they had at least latent TB infection that may have progressed into clinically active TB disease in an unknown number of cases. As it has been highlighted by Hershkovitz & co-workers [11] regarding the Hamann-Todd Human Osteological Collection: “The presence of SES only among modern adults is explained by the nature of the HTH sample, i.e., comprised of black and white men and women born between the years 1825–1910 (prior to the initiation and widespread application of antimicrobial therapy). Moreover, all were of low socioeconomic status, living in a highly polluted urban industrial area. Of the 3,100 documented subjects in the collection, 570 had reportedly died of TB. The true number is probably much higher.”. Or by Dangvard Pedersen & colleagues [13] regarding the Robert J. Terry Anatomical Skeletal Collection: “Considering when and where these people lived, many of those who were not listed as sufferers of TB, and possibly all of them, were exposed to M. tuberculosis complex bacteria at some point in their lives. That is because TB was widespread in 18th to early-20th century North America, Europe, and Africa (Bates and Stead, 1993; Daniel, 2009; Madsen et al., 1942). It is likely that TB was not noted in the sparse documentation available on some individuals who had the disease with its accompanying skeletal lesions. A more immediate or obvious cause of death could have been reported, TB might not have been mentioned because of social stigma associated with acknowledging and reporting it, or the disease was not identified because diagnostic procedures were inexact and not always thorough.”.*

*Based on the above, if Genchi & co-workers’ [7] hypothesis would be correct and GIs would be signs of TB infection rather than clinically active TB(M) disease, the frequency of cases with GIs in the non-TB groups should be very similar to their frequency in the TB groups (as in the non-TB groups, the majority of the individuals (if not all of them) are likely to had at least latent TB infection, whereas in the TB groups, all individuals are unquestionably to had not only TB infection but clinically active TB disease). However, as it has been presented by Spekker & colleagues [5], in the Robert J. Terry Anatomical Skeletal Collection, the frequency of cases with GIs was about 10 times higher in the TB group than in the non-TB group. This difference between the two groups was statistically extremely significant (“The χ^2^ testing of the frequencies of GIs in individuals with TB as the cause of death and individuals with NTB causes of death revealed a statistically extremely significant difference between the two groups (χ^2^ = 47.922, df = 1, P<0.0001).”) [5], which confirms that GIs are signs of clinically active TB(M) disease rather than TB infection;*” (lines 263–301); and

“*If Genchi & co-workers’ [7] hypothesis would be correct and it would be the meningeal tubercles developing at the beginning of the TB infection rather than the meningeal tubercles forming during the pathogenesis of TBM that exert pressure on the adjacent endocranial surface for a long time (not weeks but more likely years or decades); and thus, lead to the development of GIs, the frequency of cases with GIs should be much higher not only in the non-TB groups of the Hamann-Todd Human Osteological Collection and Robert J. Terry Anatomical Skeletal Collection, where the majority of the individuals (if not all of them) are likely to had at least latent TB infection, but also in the TB groups, where all individuals are unquestionably to had not only TB infection but clinically active TB disease. However, as it has been presented by Spekker & colleagues [5], even in the TB group of the Robert J. Terry Anatomical Skeletal Collection, where everyone was unquestionably infected by TB, GIs were present in less than one-third of the examined cases (68/234; ~29%);*” (lines 302–314).

In summary, our three counterarguments outlined above specifically address Genchi & co-workers’ (2025) hypothesis that endocranial granular impressions are only signs of tuberculosis infection rather than clinically active tuberculosis disease (specifically tuberculous meningitis). The presence of endocranial granular impressions in a few individuals from the non-TB groups of the Hamann-Todd Human Osteological Collection and the Robert J. Terry Anatomical Skeletal Collection (only 6 out of nearly 200 in the latter) is not surprising, as we only know their cause of death and not their full medical history. Some individuals may have had clinically active tuberculosis disease (tuberculous meningitis) but died of another cause (e.g., accident, cancer or cardiovascular disease), or their cause of death may have been inaccurately recorded.

Furthermore, these pre-antibiotic-era documented human skeletal collections include individuals from times and regions where exposure to tuberculosis was highly likely for nearly everyone, meaning that the majority, if not all, were probably infected with tuberculosis regardless of their recorded cause of death. Therefore, if Genchi & colleagues’ (2025) hypothesis were correct, and endocranial granular impressions merely indicated tuberculosis infection rather than clinically active tuberculosis disease (specifically tuberculous meningitis), they should have occurred at similar frequencies in both the TB group (all certainly infected) and the non-TB group (most, if not all, likely infected). However, in the Robert J. Terry Anatomical Skeletal Collection, endocranial granular impressions were about 10 times more frequent among individuals who died of tuberculosis, a difference that was statistically extremely significant. Moreover, since everyone in the TB group was certainly infected and had progressed to clinically active tuberculosis disease, endocranial granular impressions would be expected to occur even more frequently in this group; yet they were observed in less than one-third of the cases.

Unfortunately, it appears that you did not engage with these three counterarguments, as they were neither acknowledged, refuted, nor accepted. Therefore, similar to our other points, we conclude that their importance can best be emphasised – and the risk of them being overlooked avoided – by including a shorter, more concise version of the two counterarguments currently only in *S1 Text* in the main text of the revised version of our manuscript (lines 514–540):

“*The Hamann-Todd Human Osteological Collection and the Robert J. Terry Anatomical Skeletal Collection were established when and where many people, possibly everyone, were exposed to TB bacilli [102,112,158]; thus, individuals from the above-mentioned documented skeletal collections are likely to had TB infection – in the TB group, they were not only infected by TB but the infection progressed into clinically active TB disease in all cases, whereas in the non-TB group, they had at least latent TB infection that may have progressed into clinically active TB disease in an unknown number of cases. Based on the above, if Genchi & co-workers’ [136] hypothesis would be correct and GIs would be signs of TB infection rather than clinically active TB(M) disease, the frequency of cases with GIs in the non-TB groups should be very similar to their frequency in the TB groups. However, as it has been presented by Spekker & colleagues [104], in the Robert J. Terry Anatomical Skeletal Collection, the frequency of cases with GIs was about 10 times higher in the TB group than in the non-TB group. This difference between the two groups was statistically extremely significant, which confirms that GIs are signs of clinically active TB(M) disease rather than TB infection;*” (lines 514–528); and

“*If Genchi & co-workers’ [136] hypothesis would be correct and it would be the meningeal tubercles developing at the beginning of the TB infection rather than the meningeal tubercles forming during the pathogenesis of TBM that exert pressure on the adjacent endocranial surface for a long time (not weeks but more likely years or decades); and thus, lead to the development of GIs, the frequency of cases with GIs should be much higher not only in the non-TB groups of the Hamann-Todd Human Osteological Collection and Robert J. Terry Anatomical Skeletal Collection, where the majority of the individuals (if not all of them) are likely to had at least latent TB infection, but also in the TB groups, where all individuals are unquestionably to had not only TB infection but clinically active TB disease. However, as it has been presented by Spekker & colleagues [104], even in the TB group of the Robert J. Terry Anatomical Skeletal Collection, GIs were present in less than one-third of the examined cases;*” (lines 529–540).

Based on the above, although Genchi & colleagues (2025) formulated their hypothesis on a fundamentally flawed assumption, namely, that a lesion (i.e., endocranial granular impressions) frequency of around 50% in a non-representative subset could be extrapolated to imply a similarly high disease (i.e., tuberculous meningitis) prevalence in the once-lived population of mediaeval Pieve di Pava (far exceeding the ~1% disease prevalence observed today), they nevertheless raised an interesting question worth exploring (i.e., whether endocranial granular impressions represent only tuberculosis infection or indicate clinically active tuberculosis disease, specifically tuberculous meningitis). However, the currently available evidence contradicts their hypothesis on multiple grounds, whereas the earlier, long-standing interpretation (Schultz, 1999, 2001, 2003; Schultz & Schmidt-Schultz, 2015; Spekker & co-workers, 2020a, 2022) that endocranial granular impressions are signs of clinically active tuberculosis disease (specifically tuberculous meningitis) remains well-supported.

We hope that, after considering our detailed responses to your comments, you will find this earlier, long-standing hypothesis reasonable as well.

d) As for your comment “*On the other hand, in the work of Spekker et al., 2020 carried out on the Robert J. Terry Anatomical Skeletal Collection, the prevalence of GIs was observed on individuals with a diagnosis of TB, not TB meningitis; this is a fundamental difference.*”, we appreciate this observation. However, it is not entirely clear to us why the fact that, in the studies of Spekker & colleagues (2020a, 2022) on the diagnostic value of endocranial granular impressions, conducted on the Robert J. Terry Anatomical Skeletal Collection, the recorded cause of death for some individuals was only “tuberculosis” rather than specifically “tuberculous meningitis” should represent a methodological issue.

As we mentioned above, in response to another of your comments, tuberculosis is an extremely variable disease: there are individuals in whom only the pulmonary form (i.e., pulmonary tuberculosis) develops, others in whom only one or more extrapulmonary manifestations (e.g., skeletal tuberculosis or tuberculous meningitis) occur, and there are also individuals who present with both pulmonary and one or more extrapulmonary forms concurrently. The latter are often categorised simply as cases of pulmonary tuberculosis, even if one or more extrapulmonary manifestations (e.g., skeletal tuberculosis or tuberculous meningitis) are also present; in addition, when an individual presents with extrapulmonary tuberculosis affecting multiple sites, the case is categorised according to the site considered to represent the most severe manifestation of the disease (Hoffmann & Churchyard, 2009; Lee, 2015; Kimera & co-workers, 2022;

<https://iris.who.int/bitstream/handle/10665/366365/9789240068919-eng.pdf?sequence=1>; <https://www.ncbi.nlm.nih.gov/books/NBK138741/>).

Therefore, the fact that only “tuberculosis” was recorded as the cause of death, or that a specific form of tuberculosis different from tuberculous meningitis (e.g., pulmonary tuberculosis or skeletal tuberculosis) was noted, does not mean that the individual could not have suffered from tuberculous meningitis at the time of death. Interestingly, Genchi & colleagues (2025) themselves acknowledge that “*In fact, data from the pre-antibiotic era corresponds to current clinical data, showing that TB meningitis was often a terminal event in adults with chronic pulmonary TB (Taylor, 1908; Warrington, 1910).*”, referring to the well-established observation that tuberculous meningitis often develops as a late complication of pulmonary tuberculosis – an association consistently supported by data from both the pre-antibiotic era and modern medical literature. Nevertheless, Genchi & co-workers (2025) disregard, without any justification, the possibility that this very relationship could readily explain why Spekker & colleagues (2020a) found a relatively high frequency (~29%) of cases with endocranial granular impressions among individuals who died of tuberculosis in the Robert J. Terry Anatomical Skeletal Collection, even though tuberculous meningitis was recorded as the cause of death in only a few of these cases, while in most, pulmonary tuberculosis, other forms of tuberculosis, or simply “tuberculosis” was listed.

It should also be emphasised that, beyond the studies by Spekker & co-workers (2020a, 2020b, 2021, 2022), the Robert J. Terry Anatomical Skeletal Collection has also been widely used as a reference sample by numerous other researchers over the past decades to investigate the diagnostic value of bony changes associated with either skeletal tuberculosis or with pulmonary tuberculosis/tuberculous pleurisy (e.g., Roberts & colleagues, 1994; Winland & co-workers, 1997; Dangvard Pedersen & colleagues, 2019). This highlights that the Terry Collection is a well-established and broadly accepted comparative dataset for studying skeletal lesions related to different forms of tuberculosis, not only by Spekker & co-workers (2020a, 2020b, 2021, 2022) but by other authors as well.

We would also like to respectfully note that in Spekker & colleagues (2020a), the authors did not determine the prevalence of cases with endocranial granular impressions, but rather assessed the frequency of cases with such lesions in individuals who died of tuberculosis (~29%) and in those who died of other, non-tuberculous causes (~3%), and subsequently compared these frequencies between the two groups.

e) Regarding your comment, *“Additionally, nor in that work nor in previous works reporting GIs as related to TB meningitis (Schultz, 1999; Schultz, 2001; Schultz, 2003; Schultz and Schmidt-Schultz, 2015) it was discussed on a clinical and pathophysiological basis the reason why GIs were supposed as a manifestation of TB meningitis”*, it seems that you are stating that previous studies did not provide a justification for interpreting endocranial granular impressions specifically as signs of tuberculous meningitis, either based on clinical observations or on an understanding of the underlying pathophysiological mechanisms.

However, this assertion is not true.

In Schultz (1999):

“*However, there are also specific changes, which are only caused by a tuberculous process of the meninges. These characteristic lesions are small, roundish, and relatively flat impressions in the endocranial lamina of the skull and* ***are caused by pressure atrophy of the tuberculars*** *(Figures 1, 2).*” (page 504); and

“*As a rule, pressure atrophy is a relatively slow process. In leptomeningitis tuberculosa, the tuberculars which cause the impressions grow relatively slowly. Therefore, the bone has the time to organize and to adapt to the new situation by building up mature structures such as regular laminae (Figure 3).*” (pages 505–506).

In Schultz (2001):

“*Vestiges of tuberculous meningitis are characterized not only by the changes described above, but also by relatively small, granular impressions whose diameter varies between 0.5–1.0 mm (Fig. 11C–F). Microscopically, the lesions are very characteristic and a reliable indicator of Leptomeningitis tuberculosa, because in polarized light, the intra vitam origin of these impressions can be easily differentiated (Fig. 21B,C). It is striking that these lesions could apparently be organized (remodeling) after a while (Fig. 21C) if the child survived long enough. The localization of these impressions, which are generally grouped in clusters, is very characteristic. They are situated at the endocranial face of the base, sometimes also at the lateral wall of the skull, especially in the middle and the posterior cranial fossa, and occasionally also in the cerebral fossa of the occipital bone (Schultz, 1999a; Templin, 1993; Templin and Schultz, 1994; Teschler-Nicola et al., 1994, 1996).* ***These lesions are caused by pressure atrophy of the tuberculars.*** *They represent specific features and are characteristic of this kind of tuberculous disease.*” (page 129).

In Schultz (2003):

“*Manifestations of tuberculous meningitis are characterized not only by the changes described (Figure 6-38), but also by relatively small, granular impressions that have diameters that vary between 0.5 and 1.0 mm (Figure 6-39 and 6-40). Microscopically, the lesions are very characteristic and a reliable indicator of Leptomeningitis tuberculosa because in polarized light, the intra vitam origin of these impressions can be easily differentiated (Figure 6-39). The fact that these lesions could apparently be organized (remodeled) after a certain lapse of time (Figure 6-40) if a child could survive long enough is interesting. The localization of these impressions, which are generally grouped in clusters, is very characteristic. They are situated at the endocranial surface of the base, sometimes also at the lateral wall of the skull, especially in the middle and the posterior cranial fossa, and occasionally also in the cerebral fossa of the occipital bone (Templin 1993; Templin and Schultz 1994; Teschler-Nicola et al. 1994, 1996; Schultz 1999a, 2001b).* ***They are caused by "pressure atrophy" of the tuberculars.*** *They are a specific feature and are characteristic of this kind of tuberculous disease.*” (pages 94–95).

In Schultz & Schmidt-Schultz (2015):

“*Flat, roundish impressions, mainly observed on the endocranial surface of the skull base, might be vestiges of L. tuberculosa (Figure 3aec). The lesions occur isolated (Figure 3c) or as confluent groups (Figure 3a). As a rule, the rims and the walls of the impressions are smooth. Therefore, in scanning-electron microscopic images (Figure 3aec), the impressions look as though* ***they were established relatively slowly by pressure atrophy*** *[3,5]. The light microscopic investigation of thin-ground sections, particularly in polarized light, reveals interesting features (Figure 4). There is no doubt that these impressions did develop intra vitam and that their walls grew slowly because they are surrounded by mature lamellar bone and collagen fibers following the contours of these impressions (Figure 4aec).*” (page 3);

“*Figure 5. A. Case 5:* ***Cranium of a 22-year-old German male, Collection of the Department of Anthropology of the Museum of Natural History Vienna (Austria), 1877 (Ind. 59), known clinical diagnosis of TB.*** *Occipital squama. Basilar region of left cerebral fossa and medial part of the left transverse sinus. In the cerebral fossa, there are* ***impressions of probable tubercles*** *and fine blood vessels which originated during the inflammatory process. The bony wall of the sinus shows vestiges of scarring. B. Male 25–30-year old, Burial 143, S-2, excavated from the medieval cemetery of Greding, Bavaria (Germany), 6the8th century AD. Endocranial cast of the left anterior cranial fossa: the superficial surface of the basilar part of the left frontal lobe of the brain exhibits numerous small formations which probably represent tubercles caused by Leptomeningitis tuberculosa. C. Basilar meningitis (Leptomeningitis tuberculosa). Small tubercles are situated at the base of the brain. Modified illustration according to Fig. 7 of Frank H. Netter, Farbatlanten der Medizin, Bd. 6, Nervensystem II (Krämer G, editor). Stuttgart/New York: G. Thieme Verlag 1989. p. 164.” (page 5)*; and

“*For the purpose of comparison,* ***recent skulls from anatomical pathological collections with known clinical diagnosis of TB exhibiting such endocranial lesions*** *were selected (Figure 5a). From endocranial regions affected by these impressions, casts were prepared to give a three-dimensional feeling of the character of theses lesions which, indeed, look like tubercles known from TB (Figure 5b). Furthermore, figures from the literature were used (Figure 5c) to compare the morphology of the structures of the casts with the morphology of known cases illustrated by drawings or photographs which also show tubercles pathognomonic of TB. Additionally, thin-ground sections were prepared from the recent skulls to check whether the morphology of the impressions at the micro-level observed in the archaeological cases might be the same as in the recent skulls with known clinical diagnosis TB (Figure 6). Indeed, the morphology in both groups, the archaeological and the recent specimens, is morphologically identical and the changes originated in all cases during the lifetime of the individuals. Thus, the identical macro- and micro-morphology of the impressions might allow the conclusion that they were caused by the tubercles, one of the pathognomonic features of TB.*” (page 6).

In Spekker & colleagues (2020a):

“*Schultz [24–26] and Schultz & Schmidt-Schultz [27] have identified granular impressions (GIs) on the inner surface of the skull as one of these bony changes. GIs are small (0.5–1.0 mm in diameter), relatively shallow (less than 0.5 mm in depth), roundish impressions with smooth margins and walls [24–28,30]. They represent a pathological process that affects only the superficial part of the inner lamina of the skull with no diploic and/or ectocranial involvement [24–28,30]. GIs generally appear as isolated or confluent lesions grouped in clusters on the inner surface of the skull base or sometimes of the lower lateral skull vault: they are particularly situated in the orbital part of the frontal bone, the greater wings of the sphenoid bone, the squamous part of the temporal bones, and the lateral and squamous parts of the occipital bone [24–28]. According to the results of Templin [28], Templin & Schultz [29], Schultz [24–26], and Schultz & Schmidt-Schultz [27],* ***GIs may be established by pressure atrophy of the tubercles formed in the dura mater during later stages of TBM*** *[24–29].*” (page 3);

“*In the paleopathological literature, GIs (sharply demarcated erosive defects [31]) on the inner surface of the skull have been considered as pathognomonic features of TBM–****established by pressure atrophy of the tubercles formed in the dura mater (in close vicinity to blood vessels affected by TBM) during later stages of the disease*** *[24–27].*” (page 6);

“*TBM usually develops subsequent to the rupture of one or more meningeal, subpial, and/or subependymal caseating Rich foci into the subarachnoid space or into the ventricular system, both occupied by the cerebrospinal fluid (CSF) [43–45,49,52–56]. The release of sufficient numbers of TB bacteria into the CSF triggers the onset of diffuse granulomatous inflammation of the leptomeninges (i.e., the pia and arachnoid mater), with a strong predilection for the basal areas of the brain [43–45,49,52–55]. In earlier stages of the disease, the pathological process extends predominantly along the blood vessels of the leptomeninges (particularly of the pia mater) [34]. TBM is characterized by the formation of tubercles on the pia and arachnoid mater [34,57]. Nonetheless, not only the leptomeninges but additionally, the outermost meningeal layer (i.e., the dura mater) that is directly adherent to the inner surface of the skull, can be affected by tubercles in later stages of TBM [35,57]. Besides the small tubercles formed in the meninges, characteristic pathological features of TBM include enhancing basal meningeal exudate, progressive hydrocephalus, and vasculitis of blood vessels adjacent to or traversing the exudate [34,43,45,52,55– 56]; as a rule, the formation of tubercles precedes the development of the latter ones [35].*” (page 9); and

“*On the one hand, bony changes associated with TBM were distinctly described in the pathological literature from the first half of the 20th century (preceding the introduction of antibiotics in the management of TB) [e.g., 34–36].* ***At autopsy of TBM patients, groups of isolated but mostly confluent, small, and dimpled impressions established by pressure atrophy of the tubercles (i.e., GIs)****, vestiges of hemorrhages developed in close vicinity to the affected blood vessels (i.e., abnormal blood vessel impressions and periosteal appositions), and certain roughnesses indicating characteristic resorption of the bone tissue (i.e., very flat and small erosive bone loss that can be recognized only with low-power microscopy) were observed on the endocranial surface of the skull base and of the lower lateral skull vault after the removal of the dura mater [34–36]. However, autopsy practices have changed over time, and in the present, the dura mater is not completely removed from the basal areas of the skull; thus, there is no detection of the aforementioned bony changes on the endocranial surface.*” (pages 9–10).

We would also like to note that at least three additional publications (Spekker & co-workers, 2022, 2024; Madai & colleagues, 2025), several of whose co-authors also contributed to the works cited by you, similarly provide a detailed discussion of the pathophysiological basis that you suggest is lacking.

In Spekker & co-workers (2022): in Table 1 (“*Endocranial alteration types indicative of TBM*”), in the pathophysiology column regarding endocranial granular impressions: “*Can be established by pressure atrophy of the tubercles formed on the outermost meningeal layer during later stages of TBM.*” (page 446).

In Spekker & colleagues (2024):

“*In all five cases, the pathogenic progression of this TB disease would have been that: via haematogenous or lymphogenous spread from the primary site of the infection (very likely the lungs), TB bacilli would have reached the central nervous system [66, 70–80]. Once there, the presence of the pathogens would have triggered the formation of small (0.5–2 mm) tubercles (i.e., Rich foci) in the meninges, and the brain and/or spinal cord parenchyma [66, 70–80]. Subsequently, TB bacilli would have been released into the cerebrospinal fluid (by rupture of one or more caseating Rich foci into the subarachnoid space or the ventricular system), triggering granulomatous inflammation of the leptomeninges (i.e., the pia and arachnoid mater encephali) and the tubercle formation on them [66, 72–82]. It should be noted that besides the enhancing basal meningeal exudate and vasculitis of the blood vessels adjacent to or traversing the exudate, the meningeal tubercles are one of the characteristic pathological features of TB meningitis [66, 72, 75, 78, 81–83]. In later stages of the disease, not only the leptomeninges but also the outermost meningeal layer (i.e., the dura mater encephali) could have become affected by the pathological process [66, 84, 85].* ***As the dura mater encephali is directly adherent to the inner surface of the cranium, the gradually growing and coalescing meningeal tubercles could have exerted localised pressure on the underlying bone, inducing temporary, circumscribed bone atrophy and subsequent formation of endocranial GIs (Figs 2, 4, 5, 6A, 6B, 6D–6E, 15 and 17) [62, 65, 66, 69].*** *By definition, GIs are small (0.5–1.0 mm in diameter), relatively shallow (less than 0.5 mm in depth), roundish impressions with smooth margins and walls [62–66, 69]. They can appear separately or confluently and are usually grouped in clusters on the inner surface of the cranium [62–66, 69].* ***Granular impressions match in their position to those tubercles of the dura mater encephali, which cause bone resorption by exploring pressure on the underlying bone [62–66, 69].***” (page 11).

In Madai & co-workers (2025):

“*What could have happened in the case of KD429 is that following the haematogenous or lymphogenous spread of TB bacilli from the primary site of the infection to the central nervous system, the presence of the pathogens would have triggered the formation of small (0.5–2 mm) tubercles (Rich foci) around them in the meninges, or the brain or spinal cord parenchyma [76–86]. Later, the release of sufficient numbers of TB bacilli into the cerebrospinal fluid due to the rupture of one or more caseating Rich foci into the subarachnoid space or the ventricular system would have triggered the onset of granulomatous inflammation of the leptomeninges (the pia and arachnoid mater encephali) with subsequent tubercle formation on them [78–88]. It should be noted that meningeal tubercles are one of the characteristic pathological features of TBM – besides an enhancing basal meningeal exudate and vasculitis of the blood vessels adjacent to or traversing the exudate [78,81,84,87–89]. In later stages of the disease, not only the leptomeninges but also the outermost meningeal layer (the dura mater encephali) would have become affected by the pathological process [90,91].* ***As the dura mater encephali is directly adherent to the inner surface of the skull, the gradually growing and coalescing tubercles formed on it would have exerted localised pressure on the underlying bone (endocranial surface), which could have induced temporary, circumscribed bone atrophy [28,32,33,36]. Consequently, endocranial GIs (Figs. 4–6) would have developed.*** *These small (0.5–1.0 mm in diameter), relatively shallow (less than 0.5 mm in depth), roundish impressions with smooth margins and walls can appear separately or confluently, and are usually grouped in clusters [28–30,32,33,36].* ***Granular impressions match in their position to the tubercles of the dura mater encephali that could have caused the bone resorption by exploring pressure on the inner skull surface.***” (page 4).

Importantly, in the original version of our manuscript (lines 328–334), we also addressed the pathophysiology of endocranial granular impressions in tuberculous meningitis, and, following the request of **Reviewer 2**, now we included two figures (*S2 Figure* and *S3 Figure*) illustrating how endocranial granular impressions may develop in cases of tuberculous meningitis in the revised version:

“*In addition to the above, the presence of endocranial alterations indicative of TBM (GIs and ABVIs) in multiple locations on the inner skull surface of MMG3-75 (Figs 4–6) suggests that the TB infection may have extended from the clivus towards the meninges, at least the dura mater encephali. In the palaeopathological literature, endocranial GIs have been described as specific signs of TBM [91-93,95-96,99].* ***Their development is secondary to the pressure atrophy of the cortical bone layers, caused by the tubercles formed on the dura mater encephali as a result of its TB granulomatous inflammation [91,95-96,99].***” (lines 328–334 in the original version of our manuscript).

**REFERENCES:**

Dangvard Pedersen D, Milner GR, Kolmos HJ, Boldsen JL. 2019. The association between skeletal lesions and tuberculosis diagnosis using a probabilistic approach. *International Journal of Paleopathology* **27**: 88–100. DOI: 10.1016/j.ijpp.2019.01.001

Genchi ML, Giuffra V, Campana S, Riccomi G. 2025. Are endocranial granular impressions pathognomonic of tuberculous meningitis or a marker of tuberculous infection? An investigation on a medieval osteoarcheological assemblage from Italy. *International Journal of Paleopathology* **49**: 81–92. DOI: 10.1016/j.ijpp.2025.03.002

Hershkovitz I, Greenwald CM, Latimer B, Jellema LM, Wish-Baratz S, Eshed V, Dutour O, Rothschild BM. 2002. *Serpens endocrania symmetrica* (SES): A new term and a possible clue for identifying intrathoracic disease in skeletal populations. *American Journal of Physical Anthropology* **118**(3): 201–216. DOI: 10.1002/ajpa.10077

Hoffmann CJ, Churchyard GJ. Pulmonary tuberculosis in adults. In: Schaaf HS, Zumla AI, editors. *Tuberculosis. A comprehensive clinical reference*. Amsterdam, The Netherlands: Elsevier; 2009. pp. 332–341. DOI: 10.1016/B978-1-4160-3988-4.00029-9

Kolbinger R, Heindel W, Pawlik G, Schröder R. 1994. Tuberculous meningoencephalitis with primary destruction of the cranial base and secondary development of multiple tuberculomas. *Nervenarzt* **65**(2): 132–135.

Kimera CL, Sinyinza F, Lukolo LN. 2022. Extra-pulmonary tuberculosis in the uterine cervix: A case report. *Case Reports in Clinical Medicine* **11**(10): 409–413. DOI: 10.4236/crcm.2022.1110057

Lee JY. 2015. Diagnosis and treatment of extrapulmonary tuberculosis. *Tuberculosis and Respiratory Diseases* **78**(2): 47–55. DOI: 10.4046/trd.2015.78.2.47

Madai Á, De Andrés Montero M, Kis L, Szalontai C, Szigeti A, Major I, Kiss PA, Spekker O. 2025. Chasing the “White Plague” in the Barbaricum of the Carpathian Basin – A case with tuberculous meningitis discovered in a Sarmatian-period (2^nd^–4^th^-century-CE) storage pit from the archaeological site of Kiskundorozsma–Daruhalom-dűlő II (Hungary). *Tuberculosis* **152**: 102632. DOI. 10.1016/j.tube.2025.102632

Milner GR, Boldsen JL. 2017. Life not death: Epidemiology from skeletons. *International Journal of Paleopathology* **17**: 26–39. DOI: [10.1016/j.ijpp.2017.03.007](https://doi.org/10.1016/j.ijpp.2017.03.007)

Park JH, Park K, Eom M. 2024. Tuberculous meningoencephalitis with skull perforation possibly mistaken for trauma: An autopsy case report. *Korean Journal of Legal Medicine* **48**(4): 175–179. DOI: [10.7580/kjlm.2024.48.4.175](https://doi.org/10.7580/kjlm.2024.48.4.175)

Roberts CA, Lucy D, Manchester K. 1994. Inflammatory lesions of ribs: An analysis of the Terry Collection. *American Journal of Physical Anthropology* **95**(2): 169–182. DOI: 10.1002/ajpa.1330950205

Roberts CA, Pfister L-A, Mays S. 2009. Letter to the editor: Was tuberculosis present in *Homo erectus* in Turkey?. *American Journal of Physical Anthropology* **139**: 442–444. DOI: 10.1002/ajpa.21056

Schultz M. The role of tuberculosis in infancy and childhood in prehistoric and historic populations. In: Pálfi G, Dutour O, Deák J, Hutás I, editors. *Tuberculosis: Past and present*. Szeged, Hungary: TB Foundation & Budapest, Hungary: Golden Book Publisher; 1999. pp. 503–507.

Schultz M. 2001. Paleohistopathology of bone: A new approach to the study of ancient diseases. *American Journal of Physical Anthropology* **116**(Suppl. 33): 106–147. DOI: 10.1002/ajpa.10024

Schultz M. Light microscopic analysis in skeletal paleopathology. In: Ortner DJ, editor. *Identification of pathological conditions in human skeletal remains*. San Diego, CA, USA: Academic Press; 2003. pp. 73–107. DOI: 10.1016/B978-012528628-2/50043-0

Schultz M, Schmidt-Schultz TH. 2015. Is it possible to diagnose TB in ancient bone using microscopy?. *Tuberculosis* **95**(Suppl. 1): S80–S86. DOI: 10.1016/j.tube.2015.02.035

Spekker O, Hunt DR, Paja L, Molnár E, Pálfi G, Schultz M. 2020a. Tracking down the White Plague: The skeletal evidence of tuberculous meningitis in the Robert J. Terry Anatomical Skeletal Collection. *PLOS ONE* **15**(3): e0230418. DOI: 10.1371/journal.pone.0230418

Spekker O, Schultz M, Paja L, Váradi OA, Molnár E, Pálfi G, Hunt DR. 2020b. Tracking down the White Plague. Chapter two: The role of endocranial abnormal blood vessel impressions and periosteal appositions in the paleopathological diagnosis of tuberculous meningitis. *PLOS ONE* **15**(9): e0238444. DOI: 10.1371/journal.pone.0238444

Spekker O, Hunt DR, Berthon W, Paja L, Molnár E, Pálfi G, Schultz M. 2021. Tracking down the White Plague. Chapter three: Revision of endocranial abnormally pronounced digital impressions as paleopathological diagnostic criteria for tuberculous meningitis. *PLOS ONE* **16**(3): e0249020. DOI: 10.1371/journal.pone.0249020

Spekker O, Hunt DR, Berthon W, Molnár E, Pálfi G. 2022. Insights into the diagnostic efficacy and macroscopic appearance of endocranial bony changes indicative of tuberculous meningitis: Three example cases from the Robert J. Terry Anatomical Skeletal Collection. *International Journal of Osteoarchaeology* **32**(2): 444–456. DOI: 10.1002/oa.3079

Spekker O, Kiss PA, Kis L, Király K, Varga S, Marcsik A, Schütz O, Török T, Hunt DR, Tihanyi B. 2024. White Plague among the “forgotten people” from the Barbaricum of the Carpathian Basin – Cases with tuberculosis from the Sarmatian-period (3^rd^–4^th^ centuries CE) archaeological site of Hódmezővásárhely–Kenyere-ér, Bereczki-tanya (Hungary). *PLOS ONE* **19**(1): e0294762. DOI: 10.1371/journal.pone.0294762

Vaid S, Lee YYP, Rawat S, Luthra A, Shah D, Ahuja AT. 2010. Tuberculosis in the head and neck – A forgotten differential diagnosis. *Clinical Radiology* **65**(1): 73–81. DOI: 10.1016/j.crad.2009.09.004

Winland K, Rothschild BM, Rothschild C. 1997. Reproducibility of patterns of tuberculosis-related periosteal reaction in the Hamman-Todd, Grant & Terry Collections. *American Journal of Physical Anthropology* **Suppl. 24**: 243.

5) **Reviewer 1** noted that “*Lastly, the final diagnosis of TB is not supported by either molecular analysis or macroscopic assessment of the postcranial bones.*”.

We thank you for this valuable and insightful comment, and would like to address separately your observations regarding the lack of success in molecular analyses and the absence of postcranial bony changes in MMG3-75.

a) It is important to emphasise that, although palaeomicrobiological analyses (e.g., aDNA- and lipid biomarker-based investigations) are highly useful to complement the findings of traditional morphology-based examinations, these approaches should not be considered exclusive or indispensable for confirming a diagnosis. As an adage notes: “*absence of evidence is not evidence of absence*” (Harkins & colleagues, 2015). In other words, in MMG3-75, the absence of detectable *Mycobacterium tuberculosis* DNA does not preclude a (morphology-based) diagnosis of tuberculosis.

The successful detection of *M. tuberculosis* aDNA in skeletal remains is often unpredictable, influenced by multiple taphonomic and biological factors, including the preservation state of the extant bones, and which skeletal elements are sampled. We discuss this issue in more detail in our response to **Reviewer 3**, who specifically inquired about the preservation of the bone and tooth samples used for aDNA analyses in MMG3-75, as well as whether the samples were taken from lesion-affected areas or from more distant regions (“*In line 203 the authors mentioned that “tooth and skull fragments” were selected for DNA analysis, but it is also important to mention which tooth and fragments were selected, their preservation and why they were selected.*”).

In summary, while molecular evidence would certainly strengthen the morphology-based diagnosis in MMG3-75, its absence should not be interpreted as definitive evidence against it. We already highlighted this aspect in the original version of our manuscript (lines 520–527):

“*The aDNA analysis could not provide evidence for the presence of Mycobacterium tuberculosis DNA in the hard tissue samples (tooth and skull fragment) taken from MMG3-75. Although aDNA analyses are very useful to complement the findings of traditional morphology-based investigations, they should not be considered as exclusive and indispensable tools to confirm a diagnosis in skeletal human remains. The successful detection of M. tuberculosis DNA in prehistoric and historic TB cases depends on a number of factors, including the state of preservation of the extant skeletal remains that was not excellent in the case of MMG3-75.*”.

Nevertheless, following the suggestion of **Reviewer 3**, we have now added a discussion of our study’s limitations in the newly created *Conclusions* section (separate from the *Discussion*), noting that the sample selection may have contributed to the negative aDNA results (lines 789–800 in the revised version of our manuscript):

“*It is important to emphasise that, although aDNA-based investigations are highly valuable in complementing the findings of traditional morphology-based examinations, they should not be considered as exclusive and indispensable tools for confirming a diagnosis of TB in skeletal human remains. The successful detection of M. tuberculosis DNA in prehistoric and historic TB cases depends on multiple factors. In MMG3-75, for instance, the aDNA analysis revealed an overall poor preservation of endogenous DNA in both samples, which may account for the negative result. Another possible explanation is that, to avoid irreversible damage to the clival bony changes through invasive sampling and to preserve these morphologically unique and diagnostically significant alterations for potential future investigations, no samples were taken directly from the affected bone area or its immediate surroundings, where the likelihood of detecting M. tuberculosis aDNA would presumably have been higher.*”.

We hope that our detailed response clarifies the limitations underlying the aDNA analysis in our study.

b) As noted above, even if postcranial lesions potentially associated with skeletal tuberculosis (e.g., vertebral or costal changes) had been present in MMG3-75, these would represent different forms of tuberculosis – for instance, vertebral changes would be consistent with spinal tuberculosis, whereas costal changes would be indicative of extra-spinal tuberculous osteomyelitis of the ribs. In contrast, the cranial alterations detected in MMG3-75 can be related to other manifestations of tuberculosis – the clival changes (mild cortical erosion and multiple, well-circumscribed osteolytic lesions) are suggestive of tuberculous clival osteomyelitis, whereas the endocranial alterations (granular impressions and abnormal blood vessel impressions) correspond to tuberculous meningitis.

Therefore, no direct causal link can be established between the postcranial bony changes you mentioned (which, in any case, were not observed in MMG3-75) and the cranial alterations actually detected in MMG3-75, as neither spinal tuberculosis nor extra-spinal tuberculous osteomyelitis of the ribs could account for the clival lesions or endocranial alterations in this younger adult male, as the former would explain only vertebral changes and the latter only costal changes.

Furthermore, different forms of tuberculosis do not necessarily occur simultaneously within the same individual. The skeletal manifestations underlying the vertebral and costal changes you mentioned (i.e., spinal tuberculosis and extra-spinal tuberculous osteomyelitis of the ribs) are distinct from those accounting for the cranial alterations observed in MMG3-75 (i.e., tuberculous clival osteomyelitis and tuberculous meningitis). Consequently, the absence of additional postcranial lesions characteristic of skeletal tuberculosis (e.g., vertebral or costal changes) in MMG3-75 is not unexpected.

**REFERENCE:**

Harkins KM, Buikstra JE, Campbell T, Bos KI, Johnson ED, Krause J, Stone AC. 2015. Screening ancient tuberculosis with qPCR: Challenges and opportunities. *Philosophical Transactions of the Royal Society B* **370**(1660): 20130622. [DOI: 10.1098/rstb.2013.0622](https://doi.org/10.1098/rstb.2013.0622)

6) **Reviewer 1** suggested that “*Page 8, table 1: “secondary hypertrophic osteoarthropathy”, not “hypertrophic pulmonary osteopathy”.*”.

We thank you for this valuable comment. Hypertrophic osteoarthropathy (HOA) is a pathological condition characterised by the classic triad of (1) digital clubbing, (2) periosteal new bone formation on the tubular bones, and (3) synovial effusions (Giancane & colleagues, 2015; Yap & co-workers, 2017; Bozzao & colleagues, 2018; Kim & Hoque, 2024); skin changes can also develop. The condition occurs either in a primary (hereditary) form (i.e., primary hypertrophic osteoarthropathy/PHO), or more commonly secondary (i.e., secondary hypertrophic osteoarthropathy) to an underlying pathology (e.g., pulmonary, cardiac, or intestinal disease) (Giancane & colleagues, 2015; Yap & co-workers, 2017; Kim & Hoque, 2024).

When secondary hypertrophic osteoarthropathy is associated with a pulmonary disease (e.g., lung cancer or chronic infectious disease, such as tuberculosis), it is referred to as hypertrophic pulmonary osteopathy (HPO) or hypertrophic pulmonary osteoarthropathy (HPOA) (Kim & Hoque, 2024). The distinction lies in the aetiological emphasis: “pulmonary” highlights the lung-origin of the secondary changes.

In *Table 1*, we list the “*signs of diffuse, bilateral, symmetrical periostitis on the diaphyses of the short and/or long tubular bones (i.e., hypertrophic pulmonary osteopathy)*” as a diagnostic criterion for pulmonary tuberculosis. As we aim to emphasise the association of the periosteal new bone formations with pulmonary disease (specifically pulmonary tuberculosis), we would like to continue using the term “*hypertrophic pulmonary osteopathy*” in our study.

**REFERENCES:**

Bozzao F, Bernardi S, Dore F, Zandonà L, Fischetti F. Hypertrophic osteoarthropathy mimicking a reactive arthritis: a case report and review of the literature. *BCM Muskuloskeletal Disorders* **19**: 145. DOI: 10.1186/s12891-018-2068-9

Giancane G, Diggle CP, Legger EG, Tekstra J, Prakken B, Brenkman AB, Carr IM, Markham AF, Bonthron DT, Wulffraat N. 2015. Prymary hypertrophic osteoarthropathy: An update on patient features and treatment. *The Journal of Rheumatology* **42**(11): 2211–2214. DOI: 10.3899/jrheum.150364

Kim J, Hoque F. 2024. Hypertrophic pulmonary osteoarthropathy – An overlooked clue of lung cancer. *Brown Hospital Medicine* **3**(3): . DOI: [10.56305/001c.117078](https://doi.org/10.56305/001c.117078)

Yap FY, Skalski MR, Patel DB, Schein AJ, White EA, Tomasian A, Masih S, Matcuk GR. 2017. Hypertrophic osteoarthropathy: Clinical and imaging features. *RadioGraphics* **37**(1): 157–195. DOI: 10.1148/rg.2017160052

7) **Reviewer 2** commented that “*First of all, I would like to congratulate the authors for their unique case and for the thorough research carried out. The paleopathological alterations observed in MMG3-75 are indeed intriguing representation of tuberculosis infection, which has been unseen or remained unnoticed in medieval bioarchaeological research until now. The findings are well documented, the descriptions are clear, detailed, and precise. The authors did not restrict their research to macromorphological observation solely but organized and carried out a complex interdisciplinary analysis. While such approaches are getting more and more available, they are unfortunately still not considered standard practice. The evidences are sound and steady, enriched with a rather detailed differential diagnosis.*” and “*Finally, I would like to congratulate the authors on this high-quality work. I hope that further paleopathological analyses describing a general picture of the individuals buried in the mass graves of The Mohács National Memorial Site will be also presented in future. This paper has the potential to revive interest in macromorphological and interdisciplinary paleopathological studies, especially those focusing on tuberculous meningitis and osteomyelitis. The importance of this contribution is increasing the knowledge of the evolution of human tuberculosis over the ages, and could be published as it stands*.”.

We thank you for your thoughtful and supportive review, and are truly pleased that you found our case study unique and the interdisciplinary approach valuable. Your recognition of the efforts we made to go beyond macromorphological observations is highly appreciated.

We are also especially grateful for your kind closing remarks and encouraging words regarding the broader relevance of our work. It is truly rewarding to hear that our study may contribute to reviving interest in interdisciplinary palaeopathological research, particularly in relation to tuberculosis. Your comment reinforces our commitment to continuing investigations at the Mohács National Memorial Site, and we sincerely hope that future analyses will help present a more comprehensive picture of the individuals buried there.

8) **Reviewer 2** noted that “*Despite the complexity and the highly professional manner which are attributing the article, I would have some recommendation for improvement to be considered*. *1.) Regarding Figure 1b and Figure 1c, they are good complementary figures. However, if MMG3-75 is not fully covered on the orthophoto, it would be helpful in the positioning if the remains are highlighted or at least marked with an arrow.*”.

We thank you for this helpful and constructive suggestion. In accordance with your recommendation, we have modified *Figure 1b* to highlight the skeletal remains of MMG3-75, thereby improving the clarity of their positioning within the orthophoto. Accordingly, we have also updated the caption of *Figure 1b*, which now reads as follows:

“*b) Orthophoto of mass grave No. 3 of the Mohács National Memorial Site showing the location of the skeleton of MMG3-75 (orthophoto by Gábor Bertók and Béla Simon);*”

9) **Reviewer 2** suggested that “*2.) The description of the possible scenarios regarding pathogenesis (line 332–360) is detailed and well formulated. However, for readers with different scientific expertise it could be difficult to follow. I would suggest adding a schematic figure summarizing the process either with small pictures or boxes containing one- or two-word prompts for each step.*”.

We thank you for this valuable and insightful comment. We fully agree that a schematic summary can greatly aid readers from diverse scientific backgrounds. In response to this suggestion, we have created two schematic figures illustrating the two proposed pathogenetic scenarios. These figures have been inserted into the revised manuscript as ***S2 Figure*** and ***S3 Figure***.

“***S2 Figure:*** *A possible pathogenetic scenario that could account for the development of the cranial lesions observed in MMG3-75: tuberculosis first affected the clivus (i.e., tuberculous clival osteomyelitis) and subsequently extended to the meninges (i.e., tuberculous meningitis) (image by Marcos De Andrés Montero and Olga Spekker).*”

“***S3 Figure:*** *A possible pathogenetic scenario that could account for the development of the cranial lesions observed in MMG3-75: tuberculosis involved the meninges first (i.e., tuberculous meningitis) and then reached the clivus (i.e., tuberculous clival osteomyelitis) (image by Marcos De Andrés Montero and Olga Spekker).*”

We hope they contribute to a clearer understanding of the discussed processes.

10) **Reviewer 2** noted that “*3.) Although, the section provided for differential diagnosis (line 436–510) is thorough, scientifically accurate, and provides further assurance for the results, it does not highlight the differences and similarities, complicating the understanding. For example, when the authors disputing chordomas (line 444–457) — “Chordomas are relatively rare, malignant bone tumours of the axial skeleton that account for about 2–4% of all primary bone malignancies [149-153]. These slow-growing, locally aggressive, and highly recurrent but rarely metastasising bone tumours originate from embryonic remnants of the primitive notochord [54,58,149-152,154-156]. The skull base is affected in about one-third of all chordoma cases, with the spheno-occipital synchondrosis of the clivus being the most common site of involvement (i.e., clival chordoma) [54,55,58,149,151,155-159]. Clival chordomas are usually centrally located (midline), well circumscribed, expansile, lobulated soft-tissue masses, which cause extensive lytic bone destruction without evidence of surrounding sclerosis [54-55,58,149,155,157,160-161]. They may present at any age but most occurs in patients between the ages of 20 and 40 years [58,157,160,162-164]; there appears to be no sex difference [160,165-167]. Based on the above, clival chordoma seems to be less likely to be responsible for the development of the mild cortical erosion and the multiple, well-circumscribed osteolytic lesions observed at the clivus of MMG3-75.” — it is unclear without remembering the detailed description of the lesions which points are making the pathological phenomena relevant and which points are making it excluded from the consideration. Giving a clear comparison in the text or in the form of a table with the key similarities and differences would be beneficial.*”.

We thank you for this thoughtful and constructive suggestion. We agree that a clearer presentation of the key similarities and differences between our case and the pathological conditions considered in the differential diagnosis (chordoma, chondrosarcoma, bone metastases, and pyogenic or fungal osteomyelitides) can greatly enhance the reader’s understanding. Following your recommendation, at the end of the discussion of each potential aetiology, we have added explicit comparisons with our case, outlining the key features on the basis of which we excluded or considered these pathological conditions less likely to underlie the observed bony lesions.

“*The biological sex (male) and estimated age at death (30–39 years) of MMG3-75 do not argue against the diagnosis of clival chordoma, as these malignant bone tumours show no sex predilection and most commonly affect individuals belonging to the 20–40-year age group. In contrast, the osteolytic lesions in MMG3-75, although also lacking surrounding sclerosis as typically seen in this tumour type, display a markedly different spatial pattern. They are neither situated along the midline nor confined to the spheno-occipital synchondrosis but are instead scattered across the clivus of this younger adult male in an apparently random manner (Figs 3–4 and 8–9). Furthermore, the three larger osteolytic lesions identified within the clivus of MMG3-75 are clearly separated from one another and show no evidence of interconnection, thus lacking the lobulated appearance that would be expected in a clival chordoma (Fig 9). Taken together, the combination of the spatial distribution pattern and non-lobulated morphology of the osteolytic lesions observed in this younger adult male is difficult to reconcile with a clival chordoma, which typically arises from the spheno-occipital synchondrosis and presents as a centrally located (midline), well-circumscribed, expansile, lobulated soft-tissue mass. Based on these observations, clival chordoma can be ruled out with high certainty as a diagnostic option in MMG3-75;*” (lines 585–600 in the revised version of our manuscript);

“*Although the biological sex (male) and estimated age at death (30–39 years) of MMG3-75 do not rule out the possibility of a skull-base (clival) chondrosarcoma, he falls slightly below the typical age range (middle-aged adults) for this tumour type. Furthermore, several morphological features, particularly the spatial distribution of the osteolytic lesions in the clivus of this younger adult male, argue against this diagnosis. The well-circumscribed lesions are neither laterally located (off-midline) nor confined to the petro-clival synchondroses, but are instead randomly scattered across the clivus of MMG3-75 (Figs 3–4 and 8–9), with the three larger osteolytic lesions being spatially separate and showing no signs of interconnection. Although a larger, non-perforating osteolytic lesion is present within the clivus of this younger adult male on each side, adjacent to the petro-clival synchondroses, a skull-base (clival) chondrosarcoma would be expected to arise in the synchondral cartilage and progressively erode the surrounding bone, compromising the cortical layers and producing perforating lesions. In contrast, CBCT images (Fig 8) reveal no evidence of erosion in the bones adjacent to either petro-clival synchondrosis (neither in the laterally adjacent petrous apex nor in the medially adjacent clivus), providing strong evidence that these osteolytic lesions did not develop from the synchondral cartilage inward into the surrounding bone. The observed spatial distribution of the osteolytic lesions detected in MMG3-75, together with the intact cortical bone adjacent to the petro-clival synchondroses and the lack of the characteristic ring-and-arc calcification pattern on the CBCT images (Fig 8), is difficult to reconcile with a skull-base (clival) chondrosarcoma, which typically presents as a laterally located (off-midline), well-circumscribed, expansile, lobulated soft-tissue mass with a ring-and-arc pattern of chondroid calcification, mostly arising from the petro-clival synchondrosis. Taken together, these observations strongly suggest that a skull-base (clival) chondrosarcoma is unlikely to account for the mild cortical erosion and multiple, well-circumscribed osteolytic lesions observed in MMG3-75;*” (lines 614–638 in the revised version of our manuscript);

“*Although the biological sex (male) of MMG3-75 is consistent with the slight male predominance reported for clival bone metastases, his estimated age at death (30–39 years) is younger than that of the older individuals typically affected by this pathological condition. Furthermore, beyond the clivus, no similar osteolytic lesions were identified elsewhere in the skull or postcranial skeleton of this younger adult male, whereas secondary bone malignancies usually present as multiple alterations, most often involving different parts of the axial skeleton. Finally, all clival changes in MMG3-75 are purely lytic, with no evidence of new bone formation. However, purely osteolytic or osteoblastic metastatic lesions are exceedingly rare; in most cases, both osteoclastic resorption and osteoblastic new bone formation are present to some extent, resulting in mixed alterations. Considering the relatively young estimated age at death of MMG3-75, the purely lytic nature of the clival lesions and their restriction to the clivus (with no similar alterations detected elsewhere in the axial skeleton), bone metastasis can be regarded as a less likely diagnosis in this younger adult male;*” (lines 656–669 in the revised version of our manuscript); and

“*The estimated age at death (30–39 years) of* ***MMG3-75*** *is not in favour of the diagnosis of pyogenic or fungal skull-base (clival) osteomyelitides, as he is considerably younger than the elderly individuals typically affected by these pathological conditions. Moreover, these osteomyelitides usually arise secondary to ear or paranasal sinus infections, but CBCT images (Fig 8) show that both the middle and inner ears of* ***MMG3-75*** *were intact, and the posterior wall of the sphenoid sinus – which became visible due to post-mortem damage – was also unaffected (Fig 4b). Although marginal sclerosis, reactive new bone formation, and sequestrum are frequently observed at the osteolytic lesions in such osteomyelitides, especially in pyogenic cases, none of these features were detected in this younger adult male, as demonstrated by the CBCT images (Fig 8). Considering the relatively young estimated age at death of* ***MMG3-75****, the absence of any evidence for preceding ear (i.e., otitis media or otitis interna) or paranasal sinus infection (i.e., sphenoid sinusitis), and the lack of marginal sclerosis, reactive new bone formation or sequestrum, it is highly improbable that a pyogenic or fungal skull-base (clival) osteomyelitis was responsible for the development of the mild cortical erosion and multiple, well-circumscribed osteolytic lesions observed in this younger adult male.*” (lines 695–710 in the revised version of our manuscript).

We hope that these additions meet your expectations, and provide a clearer and more convincing argument.

11) **Reviewer 2** mentioned that “*In addition to the above, I would like to take the chance to address a question to the authors. Due to the poor preservation of endogenous DNA, the palaeogenetic analysis did not provide further evidence of probable tuberculosis infection. Have the authors considered, or are they considering, to collect further evidence based on alternative methods? Although lipid biomarker-based methods are not as commonly applied, they are well-established and provide different approaches for mycolic acid-based diagnosis (Hershovitz et al., 2008; Szewczyk et al., 2013) and mycocerosic acid-based diagnosis (Redman et al., 2009; Váradi et al., 2021).*”.

We thank you for raising this important point and for highlighting alternative molecular diagnostic methods. While lipid biomarker-based methods are indeed less commonly applied, we fully agree with you that they could provide valuable additional evidence to support the diagnosis of tuberculosis – especially in cases where endogenous aDNA preservation is poor, as in MMG3-75. We consider this a highly promising direction for future research and intend to carry out such analyses once the necessary resources and conditions become available.

It should also be emphasised that lipid biomarker analysis requires invasive sampling, i.e., the removal of bone material – preferably from the area directly affected by pathological lesions or their immediate surroundings. This preference is based on the general assumption that the likelihood of detecting pathogen-specific biomarkers is usually higher in tissues located within or adjacent to the site where the disease was active (however, previous studies (e.g., Müller & co-workers, 2014; Molnár & colleagues, 2015) have already documented instances in which bones exhibiting lesions characteristic of tuberculosis (e.g., vertebrae) did not yield detectable *Mycobacterium tuberculosis* aDNA, while it was successfully recovered from bones without visible macroscopic signs of the disease). Although the endogenous human DNA content recovered from the two hard-tissue samples in MMG3-75 (the upper right lateral incisor and a fragment from the left parietal bone) was less than 0.01%, indicating poor biomolecular preservation overall, this does not necessarily preclude the potential success of lipid biomarker analysis on samples obtained from similar, anatomically distant areas. Nevertheless, given the nature of lipid biomarker analysis, sampling from the lesion-affected area would likely offer the best chance for diagnostically meaningful results. Furthermore, targeted sampling of these bone regions would be crucial to demonstrate a direct causal link between the clival alterations and any detected remnants of *Mycobacterium tuberculosis* DNA, and hence between these bony changes and tuberculosis itself.

After careful consideration – and as explained in our response to the **Academic Editor** – we made a conscious decision to preserve the morphological integrity of the clival lesions observed in MMG3-75. Due to their unique nature and diagnostic significance, we deliberately avoided sampling from the affected bone area of MMG3-75 to ensure that these pathological lesions remain available for future investigations, including those using less-destructive (molecular) methods. As referenced, in a recently published study from our team (Tihanyi & co-workers, 2024), previous sampling directly from an affected bone area (the rhinomaxillary region) resulted in the complete destruction of diagnostically important pathological (leprosy-related) lesions, rendering re-examination impossible.

In light of this experience and the exceptional nature of the clival lesions observed in MMG3-75, we believe that preservation currently outweighs the potential benefit of invasive molecular analyses, and this is why we have opted to refrain from further destructive investigations at this stage. Nevertheless, we remain committed to exploring these approaches in the future, when less-destructive sampling may be feasible.

As mentioned in our response to the **Academic Editor**, we refer to lipid biomarker-based analyses in the newly created *Conclusions* section. Following **Reviewer 3**’s recommendations, this section now provides a detailed summary of our study’s limitations, among which the lack of lipid biomarker investigations is specifically highlighted as an important aspect for future research (lines 801–815 in the revised version of our manuscript):

“*Although the endogenous human DNA content recovered from the two hard tissue samples in MMG3-75 was less than 0.01%, indicating generally poor biomolecular preservation in this younger adult male, this does not necessarily preclude the potential success of alternative molecular diagnostic approaches, such as lipid biomarker analyses. Lipid biomarker-based investigations could provide valuable additional evidence to support the morphology-based diagnosis of TB in skeletal human remains – particularly in cases where endogenous DNA preservation is poor, as in MMG3-75. However, like aDNA analyses, these methods also require invasive sampling, preferably from the lesion-affected bone or areas adjacent to it. Targeted sampling of these bone regions would be crucial to demonstrate a direct causal link between the clival alterations and any detected remnants of Mycobacterium tuberculosis DNA, and hence between these bony changes and TB itself. Nonetheless, because of the unique nature of the clival lesions observed in MMG3-75, we believe that preservation currently outweighs the potential benefits of further invasive molecular investigations. We remain committed, however, to exploring these approaches in the future, once the necessary resources and conditions (e.g., less-destructive sampling techniques) become available;*”.

**REFERENCES:**

Molnár E, Donoghue HD, Lee OY-C, Wu HHT, Besra GS, Minnikin DE, Bull ID, Llewellyn G, Williams CM, Spekker O, Pálfi G. 2015. Morphological and biomolecular evidence for tuberculosis in 8^th^ century AD skeletons from Bélmegyer–Csömöki domb, Hungary. *Tuberculosis* **95**(Suppl. 1): S35–S41. DOI: [10.1016/j.tube.2015.02.032](https://doi.org/10.1016/j.tube.2015.02.032)

Müller R, Roberts CA, Brown TA. 2014. Biomolecular identification of ancient *Mycobacterium tuberculosis* complex DNA in human remains from Britain and continental Europe. *American Journal of Physical Anthropology* **153**(2): 178–189. DOI: [10.1002/ajpa.22417](https://doi.org/10.1002/ajpa.22417)

Tihanyi B, Samu L, Koncz I, Hergott K, Medgyesi P, Pálfi G, Szabó KÁ, Kis L, Marcsik A, Molnár E, Spekker O. 2024. A glimpse into the the past of Hansen’s disease – Re-evaluation and comparative analysis of cases with leprosy from the Avar period of the Trans-Tisza region, Hungary. *Tuberculosis* **148**: 102552. DOI: 10.1016/j.tube.2024.102552

12) **Reviewer 3** commented that “*The reviewed manuscript is an interesting proposal of a rare location of a bone lesion related to tuberculous meningitis, not described so far. However, in my opinion, several issues need to be addressed before it can be published. Some of them are major problems concerning the differential diagnosis*.”.

**Thank you very much for your thorough review and for recognising the novelty of our case study.** We also acknowledge your concerns about several aspects of our manuscript, particularly the differential diagnosis.

We take your comments very seriously and have carefully revised the relevant sections of the manuscript in response to your suggestions. Our aim was to clarify and strengthen our arguments, and to address the issues you raised in a detailed and transparent manner. Specific responses to each of your points are provided below.

13) **Reviewer 3** noted that “*The INTRODUCTION is well written, including first a general semblance of tuberculosis at a global level nowadays, including at the end the mention of tuberculous lesion in the clivus as an extremely rare location. However, immediately after that, the aim of the paper is presented, without any clear mention about the relevance of the research or the paleopathological problem that could be solved or answered with the evidence offered by this paper. Thus, in my opinion, a major gap is detected between lines 112 and 113 of the Introduction, where the problems that this paper addresses need to be detailed.*”.

**Thank you very much for this important and insightful comment.** We appreciate your observation regarding the structure of the *Introduction* section and fully agree that a clearer articulation of the scientific relevance of our study was needed at this point. In response to your suggestion, we have inserted a new paragraph between lines 112 and 113 of the original version of our manuscript. This additional part (lines 118–133 in the revised version of our manuscript) explicitly outlines the scientific relevance of our research and the significance of our case:

“*Archaeological cases representing rare forms of TB, such as TB clival osteomyelitis, are of particular scientific significance, as they can expand our knowledge and understanding of how the disease manifested, progressed, and spread across space and time in past human populations. Detailed presentation and discussion of such cases can serve as valuable references in the palaeopathological evaluation of skeletal assemblages, since they facilitate the identification and interpretation of similar instances, improve diagnostic accuracy, and broaden the spectrum of TB forms recognisable in human osteoarchaeological series. Furthermore, these cases also hold relevance for modern medical practice, as they provide important comparative data that may assist clinicians in diagnosing TB manifestations that, owing to their rarity today, are almost entirely unfamiliar (e.g., TB clival osteomyelitis). Building on these considerations, the aim of our paper is to present and discuss in detail a younger adult male (MMG3-75) from the 16^th^-century-CE mass grave No. 3 of the Mohács National Memorial Site (Sátorhely, southwestern Hungary), who represents a unique manifestation of TB with concomitant involvement of the skull base (clivus) and the meninges. To the best of our knowledge, MMG3-75 is the first reported archaeological case of TB clival osteomyelitis with associated meningitis.*”.

14) **Reviewer 3** commented that “*In the MATERIAL AND METHODS, under the Material subtitle, there is not a detailed list (or at least a description) of the present bones in this particular skeleton, nor a description of the preservation of the skeleton, which is extremely necessary for a critical reading about the supported evidence, or the lack of postcranial lesions related to tuberculosis that the authors mention above in the manuscripts*.”. **Reviewer 4** also mentioned that “*In one part of the manuscript was written that “anthropological analysis was carried out on the fairly preserved and almost complete skeleton” and in the other that “It should be noted that the taphonomic damage, as well as the missing elements of the skeleton of MMG3-75 significantly hampered the macromorphological observations”. Maybe it would be useful to provide an overview of which parts of the skeleton have been preserved, either descriptively or schematically.*”.

**We thank both of you for raising this important issue and for your constructive comments regarding the description of the state-of-preservation of the skeleton of MMG3-75.** We fully agree that a clearer overview of the extant skeletal elements is essential for a better understanding of the macromorphological findings and for evaluating the absence of postcranial lesions potentially associated with tuberculosis.

In response to your comments, we have expanded the relevant part of the Methods section by including a more detailed description of the state-of-preservation of the skeleton of MMG3-75 to explain why it hampered the palaeopathological evaluation. The revised part reads as follows (lines 199–208 in the revised version of our manuscript):

“*Although the overall state-of-preservation of the skeleton of MMG3-75 was fairly good and almost complete (Fig 2), the taphonomic damage and the missing elements – particularly in skeletal regions regarded as predilection sites for TB-related lesions (e.g., vertebrae; ribs; large, weight-bearing joints; and the inner surface of the skull) significantly hampered the macromorphological observations. For instance, while most of the vertebrae and ribs were present, the latter, in particular, were extremely fragmentary and incomplete. The skull was considerably fragmentary, with some missing parts and several post-mortem damaged areas on the endocranial surface. Some of the large, weight-bearing joints also showed clear post-mortem damage (Fig 2).*”.

Additionally, to further aid the reader, we have added a photograph of the skeleton laid out in anatomical order, now included as ***Figure 2*** in the revised version of our manuscript. This visual representation is intended to complement the textual description and provide a more transparent overview of the state-of-preservation of the skeleton of MMG3-75.

“***Figure 2:*** *State-of-preservation of the skeleton of MMG3-75 (photo by Marcos De Andrés Montero, Luca Kis, and Olga Spekker).*”

We hope these additions address your concerns, and enhance the clarity and interpretability of the skeletal evidence presented in our study.

15) **Reviewer 3** mentioned that “*Also in this section, a map with the location of the archaeological site is included in Figure 1, but it could also include a location at the country or continental level that shows a more visual location to the reader.*”.

Thank you very much for this helpful observation. We regret that the originally submitted map did not clearly convey the country-level location of the site. As indicated in the caption, *Figure 1a* was intended to represent the map of Hungary with the archaeological site marked (“*Fig 1. a) Map of Hungary showing the location of mass grave No. 3 of the Mohács National Memorial Site (contains information from OpenStreetMap and OpenStreetMap Foundation, which is made available under the Open Database License);*”); we also included the borders of the country to provide a broader geographical context. However, we understand that this may not have been sufficiently visible to the reader.

In response to your suggestion, we have revised *Figure 1a* to make the borders of the country more prominent and have now explicitly labelled Hungary on the map. We hope this adjustment enhances the clarity of the figure and provides a clearer geographical reference for the location of the archaeological site (Mohács National Memorial Site) within the country.

16) **Reviewer 3** noted that “*In line 170 it is mentioned that the skeleton “is in his 30s at the time of death”, which is very ambiguous. It is necessary here to offer a wider range of estimated ages. Moreover, it is necessary to mention the methods employed for age and sex estimation, instead of just mentioning that the authors used the “methods that are among the standards of practice in the field of bioarchaeology”, as it is written in line 168.*”.
a) **Thank you very much for your valuable comment regarding the age-at-death estimation and sex determination.** In response to your suggestion, we have revised the text in the *Methods* section to clarify the estimated age-at-death of MMG3-75. Instead of stating that the examined individual “*was in his 30s at the time of death*”, we now specify that he likely died **between the ages of 30 and 39**, thereby providing a more precise and transparent age range in line with your recommendation. The revised sentence now reads (lines 190–192 in the revised version of our manuscript):

“*Based on the results of these investigations, the skeleton of MMG3-75* *presented morphological features and metric values consistent with a male who likely died between the ages of 30 and 39*.”.

b) We would also like to note that the methods applied for age-at-death estimation and sex determination were already referenced in the first part of the sentence you cited (line 168 in the original version of our manuscript) from the *Methods* section, and the corresponding references were listed in the bibliography: “*Age at death was estimated [73-77] and biological sex was determined [78-80] using macromorphological and/or metric methods that are among the standards of practice in the field of bioarchaeology*.”.

Nonetheless, we have slightly rephrased this sentence to make the applied methods clearer to the reader. The revised version now reads as follows (lines 186–190 in the revised version of our manuscript):

“*Age at death was estimated (Todd [81], Nemeskéri & colleagues [82], Brooks & Suchey [83], Buckberry & Chamberlain [84], and Schmitt [85]) and biological sex was determined (Éry & co-workers [86], Brůžek [87], and Brůžek & colleagues [88]) using macromorphological and/or metric methods that are among the standards of practice in the field of bioarchaeology.*”.

Once again, we appreciate your helpful feedback, which has contributed to improving the clarity and precision of the *Methods* section of our manuscript.

17) **Reviewer 3** mentioned that “*A minor suggestion is to include a new paragraph at the end of line 170, where the paleopathological methods are detailed*.”.

**Thank you very much for your suggestion regarding the inclusion of a separate paragraph detailing the used palaeopathological methods.** We would like to clarify that, in the original version of our manuscript, a couple of sentences following line 170 were already dedicated to outlining the approach and methods used in the palaeopathological evaluation of MMG3-75. In particular, we emphasised that the analysis focused specifically on tuberculosis-related bony changes, and that we systematically applied all diagnostic criteria for tuberculosis that have been described in the palaeopathological literature to date: “*The palaeopathological assessment following the basic anthropological analysis was carried out with the naked eye. It focused on the detection of skeletal lesions (Table 1) that have been associated with different forms of TB (pulmonary TB/TB pleurisy [e.g., 81-89], TBM [e.g., 90-99], and OATB [e.g., 100-105]) in the palaeopathological literature*.” (lines 170–174 in the original version of our manuscript).

**Reviewer 4** explicitly acknowledged this approach in their review, noting that "The authors used all known and acknowledged diagnostic criteria for tuberculous clival osteomyelitis and tuberculous meningitis, additionally illustrated with pictures and detailed explanations.".

These diagnostic criteria were not only referenced in the main text but also presented in a detailed and structured format in ***Table 1***, grouped according to the various forms of tuberculosis (e.g., osteoarticular tuberculosis and tuberculous meningitis), with corresponding references for each set of criteria. This comprehensive summary was designed to enhance transparency and reproducibility.

We hope that this clarifies our methodological approach and demonstrates our commitment to applying rigorous standards in the palaeopathological evaluation.

18) **Reviewer 3** commented that “*In line 203 the authors mentioned that “tooth and skull fragments” were selected for DNA analysis, but it is also important to mention which tooth and fragments were selected, their preservation and why they were selected*.”.
a) **Thank you very much for this important observation.** In response to your comment, and as also addressed in our reply to the **Academic Editor**, we have revised the manuscript to specify exactly which skeletal elements were selected for aDNA analysis. The **upper right lateral incisor** and a small fragment of the left **parietal bone** were used as samples. This information has been added to the revised version of our manuscript in the Palaeogenetic analysis subsection of the *Methods* section. The revised sentence now reads as follows (lines 235–237 in the revised version of our manuscript):

“*Hard tissue samples (the upper right lateral incisor and a fragment of the left parietal bone) of MMG3-75 were subjected to DNA-based analysis as described by Jäger & colleagues [118].*”.

b) As also explained in our replies to the **Academic Editor** and **Reviewer 2**, a key consideration during sample selection was to **avoid damaging the bone area affected by pathological lesions**, to preserve these morphologically unique and diagnostically significant bony changes for future investigations. For this reason, we deliberately chose a skull sample from a region **anatomically distant** from the clival lesions (a fragment of the left parietal bone), in the hope that, despite this distance, remnants of Mycobacterium tuberculosis DNA might still be detectable. Following the recommendations (e.g., Pósa & colleagues, 2012; Margaryan & co-workers, 2018; Mai & colleagues, 2020), we also collected a tooth sample (the upper right lateral incisor) in parallel with the bone sample to maximise the chance of detecting *M. tuberculosis* aDNA, since teeth are often regarded as good candidates for pathogen DNA preservation.

It should be noted that the successful detection of *M. tuberculosis* aDNA in skeletons often seems rather unpredictable, as it is influenced by multiple taphonomic and biological factors. Several studies have reported cases where samples taken from bones (e.g., vertebrae) with lesions typical of tuberculosis failed to yield *M. tuberculosis* DNA, whereas positive results were obtained from bones showing no macroscopic signs of tuberculosis (e.g., Müller & co-workers, 2014; Molnár & colleagues, 2015). Moreover, intra-bone variability has also been observed, indicating that even within the same skeletal element, DNA preservation can differ markedly across sampling locations (Jäger & co-workers, 2023).

c) Although both the selected tooth and bone fragment appeared macroscopically well-preserved, it is important to emphasise that good macroscopic preservation does not necessarily correlate with sufficient DNA preservation. In fact, DNA preservation is often more closely related to the microscopic preservation (Coulson-Thomas & colleagues, 2015). This can explain why DNA may fail to be recovered from samples that appear well-preserved macroscopically, while it can sometimes be successfully retrieved from samples that look poorly preserved macroscopically. As our case clearly demonstrates, despite the favourable macroscopic condition of the two selected hard-tissue samples, overall, both displayed poor endogenous DNA preservation.

Following your other suggestion, in the newly created *Conclusions* section (now separate from the *Discussion*), we included a discussion of our study’s limitations, among which we noted that the sample selection (a skull fragment from an area not directly affected by the pathological lesions and a tooth) may have contributed to the inability of aDNA analyses to detect remnants of *M. tuberculosis* DNA in the two hard-tissue samples taken from MMG3-75 (lines 789–800 in the revised version of our manuscript):

“*It is important to emphasise that, although aDNA-based investigations are highly valuable in complementing the findings of traditional morphology-based examinations, they should not be considered as exclusive and indispensable tools for confirming a diagnosis of TB in skeletal human remains. The successful detection of M. tuberculosis DNA in prehistoric and historic TB cases depends on multiple factors. In MMG3-75, for instance, the aDNA analysis revealed an overall poor preservation of endogenous DNA in both samples, which may account for the negative result. Another possible explanation is that, to avoid irreversible damage to the clival bony changes through invasive sampling and to preserve these morphologically unique and diagnostically significant alterations for potential future investigations, no samples were taken directly from the affected bone area or its immediate surroundings, where the likelihood of detecting M. tuberculosis aDNA would presumably have been higher.*”.

**REFERENCES:**

Coulson-Thomas YM, Norton AL, Coulson-Thomas VJ, Florencio-Silva R, Ali N, Elmrghni S, Gil CD, Sasso GRS, Dixon RA, Nader HB. 2015. DNA and bone structure preservation in medieval human skeletons. *Forensic Science International* **251**: 186–194. DOI: [10.1016/j.forsciint.2015.04.005](https://doi.org/10.1016/j.forsciint.2015.04.005)

Jäger HY, Zanotelli DA, Maixner F, Nicklisch N, Alt KW, Meller H, Pap I, Szikossy I, Pálfi Gy, Zink AR. 2023. Hit or miss – A metagenomic evaluation of intra-bone variability of host pathogen load in tuberculosis-infected human remains. *Tuberculosis* **143**(Suppl.): 102392. DOI: [10.1016/j.tube.2023.102392](https://doi.org/10.1016/j.tube.2023.102392)

Mai BHA, Drancourt M, Aboudharam G. 2020. *Molecular Genetics & Genomic Medicine* **8**(6): e1202. DOI: [10.1002/mgg3.1202](https://doi.org/10.1002/mgg3.1202)

Margaryan A, Hansen HB, Rasmussen S, Sikora M, Moiseyev V, Khoklov A, Epimakhov A, Yepiskoposyan L, Kriiska A, Varul L, Saag L, Lynnerup N, Willerslev E, Allentoft ME. 2018. Ancient pathogen DNA in human teeth and petrous bones. *Ecology and Evolution* **8**(6): 3534–3542. DOI: [10.1002/ece3.3924](https://doi.org/10.1002/ece3.3924)

Molnár E, Donoghue HD, Lee OY-C, Wu HHT, Besra GS, Minnikin DE, Bull ID, Llewellyn G, Williams CM, Spekker O, Pálfi G. 2015. Morphological and biomolecular evidence for tuberculosis in 8^th^ century AD skeletons from Bélmegyer–Csömöki domb, Hungary. *Tuberculosis* **95**(Suppl. 1): S35–S41. DOI: [10.1016/j.tube.2015.02.032](https://doi.org/10.1016/j.tube.2015.02.032)

Müller R, Roberts CA, Brown TA. 2014. Biomolecular identification of ancient *Mycobacterium tuberculosis* complex DNA in human remains from Britain and continental Europe. *American Journal of Physical Anthropology* **153**(2): 178–189. DOI: [10.1002/ajpa.22417](https://doi.org/10.1002/ajpa.22417)

Pósa A, Maixner F, Zink A, Lovász G, Molnár E, Bereczki Zs, Perrin P, Dutour O, Sola C, Pálfi Gy. 2012. Ancient human tooth samples used for TB paleomicrobial research. *Acta Biologica Szegediensis* **56**(2): 125–131.

19) **Reviewer 3** mentioned that “*The RESULTS section begins on line 218, with a statement (i.e., “the skull of MG3-75 showed numerous bony changes that can be attributed to TB”) that has to be the result of the differential diagnosis and not the starting point of the description of the skeletal evidence. Instead, just a clear description of the bone lesions needs to be detailed, as the authors did with the endocranial lesions.*”. Similarly, **Reviewer 1** also mentioned that “*The authors start from a pre-defined diagnosis (already anticipated at the beginning of the results!)*”*.*

We thank both of you for this valuable and constructive comment. In light of this, we realised that the original wording (“*The skull of MMG3-75 showed numerous bony changes that can be attributed to TB.*”) may have led to some confusion in its current form, as it could have inadvertently conveyed the impression of a predetermined diagnosis. This was certainly not our intention. We did not want to pre-empt the differential diagnosis, but to provide a brief introductory statement summarising the nature of the lesions observed in the skull of MMG3-75 – including both the clival and endocranial alterations – that are potentially consistent with tuberculosis. The cited sentence was meant to provide this statement.

As noted above, the palaeopathological analysis was focused on diagnostic criteria for tuberculosis that have been described in the palaeopathological literature to date. Accordingly, the *Results* section only reports bony changes that align with these published diagnostic criteria for tuberculosis, as detailed and grouped by disease manifestation type in *Table 1*. For this reason, we believe that an introductory statement such as the one you cited remains relevant and appropriate, as it serves to frame the subsequent detailed description of the cranial bony changes (both the clival lesions and the endocranial alterations) observed in MMG3-75.

Nevertheless, to avoid misunderstanding and to clarify that the statement is not intended to pre-empt the differential diagnosis, we have revised the sentence in question to improve its clarity, which reads now (lines 251–253 in the revised version of our manuscript):

“*Based on the diagnostic criteria compiled from the palaeopathological literature and summarised in Table 1, the skull of MMG3-75 showed numerous bony changes that are suggestive of TB.*”.

20) **Reviewer 3** commented that “*However, at the end of this first paragraph of the Results, there are no mentions of other skeletal lesions, which are described in the list of Table 1 (i.e., spinal and extraspinal bone lesions possibly related to tuberculosis). Thus, it needs to be clarified if any other bone changes were (or were not) recorded in the postcranial skeletal parts*.”.

Thank you very much for this useful and insightful comment. We agree that clarification was needed regarding the presence or absence of other bony changes, beyond those observed in the skull of MMG3-75.

In *Table 1*, we listed all diagnostic criteria for tuberculosis that have been described in the palaeopathological literature to date. This includes both cranial and postcranial lesions. However, in structuring the table, we did not group the criteria by anatomical location (i.e., cranial vs. postcranial), but rather by the forms of tuberculosis they are associated with (e.g., osteoarticular tuberculosis and tuberculous meningitis).

Among the diagnostic criteria summarised in *Table 1*, only cranial lesions were observed in the case of MMG3-75. As described in detail in the *Results* section, these included mild cortical erosion and multiple, well-circumscribed osteolytic lesions in the clivus region, as well as granular impressions and abnormal blood vessel impressions in multiple locations on the endocranial surface.

To avoid any potential misunderstanding, we have now added a clarifying sentence at the end of the *Results* section, stating explicitly that no lesions consistent with the diagnostic criteria for tuberculosis listed in *Table 1* were identified on the postcranial skeleton of MMG3-75 (lines 269–271 in the revised version of our manuscript):

“*It should be noted that no lesions indicative of TB were observed in the postcranial skeleton of MMG3-75, based on the diagnostic criteria compiled from the palaeopathological literature (summarised in Table 1)*.”.

21) **Reviewer 3** noted that “*In the DISCUSSION AND CONCLUSIONS section, again the authors begin with a statement that has to be the result of the differential diagnosis and not the first option to explain the endocranial lesions described before. This statement says, “The pathological skeletal lesions observed in the skull remains of MMG3-75 suggest that this younger adult male suffered from TB with concomitant involvement of the skull base (clivus).” I notice two main and important issues here.
    The first one is the lack of evidence that supports the hypothesis of any relationship between the lesions at the clivus and the other endocranial lesions. This is also mentioned in line 319 as a fact, without a previous differential diagnosis. A differential diagnosis of other possible causes that could have affected this particular portion of the skull is just at the very end of this section. No co-occurrence of two (or more) different causes for the recording lesions was explored.*”.
    a) We thank you for this valuable and insightful comment, and acknowledge that the sentence cited (“*The pathological skeletal lesions observed in the skull remains of MMG3-75 suggest that this younger adult male suffered from TB with concomitant involvement of the skull base (clivus) and the meninges*.”) from the beginning of the *Discussion and Conclusions* section of the original version of our manuscript may have been misleading in its original form, and we regret any confusion it may have caused. (Unfortunately, we could not locate the other problematic sentence you mentioned in line 319. The line fell within the *Palaeogenetic analysis* subsection of the *Results* section and does not contain any discussion related to the diagnosis: “*By combining the data from both hard tissue samples, approximately 3,000 human sequences were recovered, enabling genetic sex determination using the method described by Mittnik & colleagues [121].*”.)

As also noted in our response to your and **Reviewer 1**’s similar comments regarding the *Results* section, our intention was not to pre-empt the differential diagnosis, but rather to provide a brief introductory statement summarising the nature of the cranial bony changes observed on the skull of MMG3-75 – including both the clival lesions and the endocranial alterations – that are potentially consistent with tuberculosis based on the diagnostic criteria compiled from the palaeopathological literature and summarised in *Table 1*. The clival lesions are indicative of tuberculous clival osteomyelitis, while the endocranial granular impressions and abnormal blood vessel impressions are suggestive of tuberculous meningitis.

Accordingly, the sentence you cited from the beginning of the *Discussion and Conclusions* section of the original version of our manuscript was intended to express our working hypothesis that the observed cranial lesions indicate that MMG3-75 may have suffered from tuberculosis at the time of death, involving both the skull base (clivus) and the meninges. To avoid any possible misunderstanding, this sentence has been rephrased in the revised version of our manuscript (lines 348–351), which now reads as follows:

“*The pathological skeletal lesions observed in the skull remains of MMG3-75 correspond to several diagnostic criteria for TB compiled from the palaeopathological literature (summarised in Table 1), suggesting that this younger adult male may have suffered from TB with concomitant involvement of the skull base (clivus) and the meninges.*”.

b) To substantiate the aforementioned hypothesis, we employed a three-step analytical approach in the *Discussion and Conclusions* section of the original version of our manuscript. First, we explored two possible pathogenetic scenarios that could account for the development of the bony changes observed in the skull of MMG3-75, assuming that tuberculosis was indeed the underlying cause: (1) the disease originated in the clivus (i.e., tuberculous clival osteomyelitis) and subsequently extended to the meninges (i.e., tuberculous meningitis), or (2) the disease originated in the meninges (i.e., tuberculous meningitis) and subsequently extended to the clivus (i.e., tuberculous clival osteomyelitis). This analysis demonstrated that the formation of such lesions is indeed plausible in the context of tuberculosis.

After identifying these plausible pathogenetic scenarios, our second step was to evaluate whether MMG3-75 indeed suffered from TB. Unfortunately, the aDNA analyses did not yield positive results for the presence of *Mycobacterium tuberculosis* DNA in the two hard-tissue samples taken from MMG3-75. Nevertheless, the macromorphological evidence – particularly the presence of granular impressions in multiple locations on the endocranial surface – supports our conclusion that this younger adult male suffered from tuberculosis at the time of death, since these lesions have long been regarded in the palaeopathological literature as specific signs of the disease (specifically tuberculous meningitis) (Schultz, 1999, 2001, 2003; Schultz & Schmidt-Schultz, 2015; Spekker & co-workers, 2020, 2022).

We acknowledge, however, that a recently published study (Genchi & colleagues, 2025) has questioned this aforementioned interpretation of endocranial granular impressions, proposing that they may indicate only tuberculosis infection rather than clinically active tuberculosis disease (specifically tuberculous meningitis). Accordingly, we addressed and critically discussed this alternative interpretation in detail in the *Discussion and Conclusions* section of the original version of our manuscript. Since we were able to refute all the arguments raised by Genchi & co-workers (2025) against the earlier hypothesis (and earlier works), we continue to endorse the original hypothesis, namely that endocranial granular impressions represent tuberculosis-specific lesions indicative of clinically active tuberculosis disease rather than only tuberculosis infection. Based on this interpretation, we can indeed conclude that a diagnosis of tuberculosis (specifically tuberculous meningitis) can be established in MMG3-75.

As a third (and final) step, we conducted a detailed differential diagnosis of the bony changes observed in the clivus of MMG3-75. We considered this necessary for precisely the reason you highlighted (“*The first one is the lack of evidence that supports the hypothesis of any relationship between the lesions at the clivus and the other endocranial lesions. This is also mentioned in line 319 as a fact, without a previous differential diagnosis. A differential diagnosis of other possible causes that could have affected this particular portion of the skull is just at the very end of this section. No co-occurrence of two (or more) different causes for the recording lesions was explored.*”). Although – based on the presence of endocranial granular impressions in multiple locations on the endocranial surface of MMG3-75 – we can conclude that this younger adult male suffered from tuberculosis (specifically tuberculous meningitis) at the time of death, which could have led not only to the formation of the endocranial granular impressions but also to that of the abnormal blood vessel impressions, this does not necessarily mean that the clival lesions were also caused by tuberculosis. It is possible that these bony changes were instead associated with another pathological condition that was concomitantly present with tuberculosis in MMG3-75. We acknowledge that the phrasing of the original sentence (“*Although based on the overall nature and association of the bony changes (mild cortical erosion and multiple, well-circumscribed osteolytic lesions) presented by the clivus of MMG3-75, as well as their co-occurrence with endocranial alterations indicative of TBM (GIs and ABVIs), TB clival osteomyelitis seems to be the most likely underlying cause, other infectious and non-infectious aetiologies should also be considered in the differential diagnosis*.”) may not have clearly conveyed this intended meaning, and we regret any confusion this may have caused. Accordingly, we have revised the text to make explicit that, in addition to tuberculosis, another concurrent pathological condition might have contributed to the development of the clival lesions in MMG3-75, which justified the need for a detailed differential diagnosis (lines 561–572 in the revised version of our manuscript):

“*Although the overall nature and co-occurrence of the clival bony changes observed in MMG3-75 (mild cortical erosion and multiple, well-circumscribed osteolytic lesions) suggest that TB (specifically TB clival osteomyelitis) could plausibly have been the underlying cause, and the presence of endocranial GIs in multiple locations on the endocranial surface of MMG3-75 provides corroborative evidence that this younger adult male suffered from TB(M) at the time of death, we cannot rule out the possibility that the clival lesions were associated with another pathological condition that was concurrently present with TB in MMG3-75. This consideration makes a detailed differential diagnosis of the clival bony changes indispensable. The most relevant infectious and non-infectious aetiologies that should be considered include* *clival malignancies (e.g., chordoma, chondrosarcoma or metastases), pyogenic osteomyelitis (e.g., caused by Pseudomonas or Staphylococcus spp.), and fungal osteomyelitis (e.g., caused by Aspergillus or Mucor spp.) [24,26,50,53,68-69].*”.

To further clarify that the sentence you cited (“*The pathological skeletal lesions observed in the skull remains of MMG3-75 suggest that this younger adult male suffered from TB with concomitant involvement of the skull base (clivus) and the meninges*.”) was intended as an initial working hypothesis, we have supplemented the *Discussion* section in the revised version of our manuscript. In this addition, we also explain how we sought to substantiate this working hypothesis in three analytical steps throughout the remainder of the *Discussion* section, including (1) reconstructing plausible pathogenetic scenarios that could account for the development of the cranial lesions observed in MMG3-75, assuming tuberculosis as the underlying cause; (2) evaluating whether MMG3-75 indeed suffered from tuberculosis at the time of death; and (3) providing a detailed differential diagnosis for the bony changes observed in the clivus of MMG3-75 (lines 351–361 in the revised version of our manuscript):

“*To substantiate this hypothesis, we employed a three-step analytical approach: (1) we attempted to reconstruct the possible pathogenetic scenarios that could account for the development of the cranial bony changes observed in MMG3-75, assuming that TB was indeed the underlying cause, thereby demonstrating that the formation of such lesions is plausible in the context of the disease; (2) we sought to provide corroborative evidence that MMG3-75 indeed suffered from TB at the time of death, thereby reinforcing that it is plausible for the disease to have been the underlying cause of the detected cranial alterations; and (3) we performed a detailed differential diagnosis of the clival lesions observed in MMG3-75, thereby taking into account the possibility that another pathological condition – concurrently present with TB in this younger adult male – could have contributed to the development of these bony changes.*”.

We sincerely thank you for this valuable and constructive comment. Your observation helped us to make the *Discussion* section much clearer and to present our line of reasoning in a more transparent and coherent way.

In line with another of your suggestions, in the newly created *Conclusions* section, which is now separate from the *Discussion*, we provide a summary of our study’s limitations, explicitly noting that uncertainties in the diagnosis of tuberculous clival osteomyelitis represent an important aspect to consider (lines 728–780 in the revised version of our manuscript):

“*Although the pathological bony changes observed in the skull remains of MMG3-75 correspond to several diagnostic criteria for TB compiled from the palaeopathological literature (summarised in Table 1), indicating that this younger adult male may have suffered from TB with concomitant involvement of the clivus (i.e., TB clival osteomyelitis) and the meninges (i.e., TBM), it was necessary to employ a three-step analytical approach to substantiate this hypothesis.*

*First, to demonstrate that the formation of the cranial lesions detected in MMG3-75 is plausible in the context of TB, we had to reconstruct the possible pathogenetic scenarios that could account for their development. Two pathways were proposed: (1) TB first affected the clivus (i.e., TB clival osteomyelitis) and subsequently extended to the meninges (i.e., TBM) (S2 Figure), or (2) it involved the meninges first (i.e., TBM) and then reached the clivus (i.e., TB clival osteomyelitis) (S3 Figure). It should be noted that although we were able to outline these potential pathogenetic scenarios, illustrating how the cranial bony changes could have developed if our hypothesis is correct (i.e., if TB was indeed the underlying cause in MMG3-75), unfortunately, we could not clarify which of these scenarios took place in MMG3-75, nor could we establish the primary focus of the TB infection within this younger adult male’s body (from where the TB bacilli presumably first reached either the clivus (S2 Figure) or the meninges (S3 Figure) via haematogenous spread).*

*Second, to reinforce that TB could indeed plausibly have been the underlying cause of the cranial lesions observed in MMG3-75, it was necessary to obtain corroborative evidence that this younger adult male suffered from the disease at the time of death. The presence of GIs in multiple locations on the inner surface of the skull of MMG3-75 provides such (macromorphological) evidence supporting a diagnosis of TB(M), which may also underlie the formation of the detected endocranial ABVIs. The observation of clival bony changes, potentially consistent with TB clival osteomyelitis, suggests that TB may have contributed not only to the development of the GIs and ABVIs but also to these lesions, and that this younger adult male may have been affected by both disease forms (i.e., TBM and TB clival osteomyelitis) simultaneously. We outlined two plausible pathogenetic scenarios explaining how the TB infection might have spread between the clivus and the meninges (either from the clivus to the meninges or vice versa), and how, in each case, the detected cranial alterations could have formed, with the GIs and ABVIs resulting from TBM and the clival bony changes from TB clival osteomyelitis (S2 Figure and S3 Figure). Nonetheless, a direct causal link between TB and the clival lesions cannot be established, as the presence of GIs in MMG3-75 provides (macromorphological) evidence only for TBM, which cannot account for the clival bony changes, and not for concurrent TB clival osteomyelitis, which could explain these alterations. Therefore, while TB clival osteomyelitis is indeed a plausible underlying cause of the observed clival lesions, we cannot conclusively assert that it occurred alongside TBM in this younger adult male, nor can we exclude the possibility that another pathological condition concomitantly present with TB in MMG3-75 contributed to the formation of these alterations.*

*Third, to substantiate that TB clival osteomyelitis was indeed the most likely primary diagnosis underlying the bony changes detected in the clivus of MMG3-75, we had to conduct a detailed differential diagnosis. During this process, both non-infectious (chordoma, chondrosarcoma, and bone metastases) and infectious (pyogenic and fungal osteomyelitides) aetiologies were considered. Taking into account the macromorphological characteristics of the alterations, together with the individual’s age at death and sex, we were able to either exclude or consider the aforementioned pathological conditions as less likely to explain the clival lesions observed in MMG3-75. Consequently, TB clival osteomyelitis is regarded as the most plausible – and therefore primary – diagnosis in the case of this younger adult male. Unfortunately, it still cannot be ruled out with absolute certainty that another pathological condition, concurrently presenting with TB in MMG3-75, contributed to the development of these bony changes, as neither the individual lesions nor their co-occurrence can be regarded as pathognomonic of TB (specifically TB clival osteomyelitis);*”.

**REFERENCES:**

Genchi ML, Giuffra V, Campana S, Riccomi G. 2025. Are endocranial granular impressions pathognomonic of tuberculous meningitis or a marker of tuberculous infection? An investigation on a medieval osteoarcheological assemblage from Italy. *International Journal of Paleopathology* **49**: 81–92. DOI: 10.1016/j.ijpp.2025.03.002

Schultz M. The role of tuberculosis in infancy and childhood in prehistoric and historic populations. In: Pálfi G, Dutour O, Deák J, Hutás I, editors. *Tuberculosis: Past and present*. Szeged, Hungary: TB Foundation & Budapest, Hungary: Golden Book Publisher; 1999. pp. 503–507.

Schultz M. 2001. Paleohistopathology of bone: A new approach to the study of ancient diseases. *American Journal of Physical Anthropology* **116**(Suppl. 33): 106–147. DOI: 10.1002/ajpa.10024

Schultz M. Light microscopic analysis in skeletal paleopathology. In: Ortner DJ, editor. *Identification of pathological conditions in human skeletal remains*. San Diego, CA, USA: Academic Press; 2003. pp. 73–107. DOI: 10.1016/B978-012528628-2/50043-0

Schultz M, Schmidt-Schultz TH. 2015. Is it possible to diagnose TB in ancient bone using microscopy?. *Tuberculosis* **95**(Suppl. 1): S80–S86. DOI: 10.1016/j.tube.2015.02.035

Spekker O, Hunt DR, Paja L, Molnár E, Pálfi G, Schultz M. 2020. Tracking down the White Plague: The skeletal evidence of tuberculous meningitis in the Robert J. Terry Anatomical Skeletal Collection. *PLOS ONE* **15**(3): e0230418. DOI: 10.1371/journal.pone.0230418

Spekker O, Hunt DR, Berthon W, Molnár E, Pálfi G. 2022. Insights into the diagnostic efficacy and macroscopic appearance of endocranial bony changes indicative of tuberculous meningitis: Three example cases from the Robert J. Terry Anatomical Skeletal Collection. *International Journal of Osteoarchaeology* **32**(2): 444–456. DOI: 10.1002/oa.3079

22) **Reviewer 3** commented that “*The second issue is the authors assume that TB affected the skeleton of this person based on the proposal that granular impressions are a pathognomonic feature of TB, when in fact it has been discussed by other authors, as the authors discussed further in this section. This is not a problem itself, and I do not have any particular problem with this hypothesis, but this paper does not offer any evidence to support that proposal or for discussing the evidence offered by other authors, as the paper published by Genchi & colleagues does. The discussion then turns to explore if these last authors are or are not right in their hypothesis, which is not the real aim of this paper. Instead, there is not enough space dedicated to the differential diagnosis and to discussing if a clivus lesion could or could not be used as a tool for paleopathological diagnosis of tuberculous meningitis.*”.
    a) Thank you very much for your detailed and thoughtful comment. We agree that the primary aim of our study is to present the case of MMG3-75, who likely suffered from tuberculosis at the time of death. The observed pathological lesions suggest that the disease affected both the clivus and the meninges, indicating concurrent tuberculous clival osteomyelitis and tuberculous meningitis. Therefore, we agree that the refutation of Genchi & co-workers’ (2025) hypothesis is not the main goal of our paper.

However, we consider this refutation an essential component of establishing a plausible diagnosis in MMG3-75. As **Reviewer 4** also pointed out: “*Special weight and importance of the work is given by Detailed refutation of Genchi & colleagues’ (2025) hypothesis that endocranial granular impressions are signs of tuberculosis infection rather than clinically active tuberculosis disease in Supporting Information where authors in eight points strengthen the previous hypothesis that GIs are pathognomonic features of TB(M) (rather than TB infection)*”.

The inclusion of this discussion is crucial because, unfortunately, aDNA analyses did not yield positive results for the presence of *Mycobacterium tuberculosis* DNA in the two hard-tissue samples taken from MMG3-75. Consequently, the only corroborative (macromorphological) evidence supporting a diagnosis of tuberculosis in MMG3-75 is the presence of endocranial granular impressions, which have been described in the palaeopathological literature as specific signs of tuberculosis (specifically tuberculous meningitis) (Schultz, 1999, 2001, 2003; Schultz & Schmidt-Schultz, 2015; Spekker & colleagues, 2020, 2022).

In contrast, the recent paper by Genchi & co-workers (2025) challenged this long-standing interpretation, proposing instead that endocranial granular impressions indicate only tuberculosis infection rather than clinically active tuberculosis disease. As we already highlighted in the *Discussion and Conclusions* section (lines 367–371 in the original version of our manuscript): “*The authors’ argument is based on that they found a quite high frequency of cases with GIs in a mediaeval (10^th^–12^th^-century-CE) osteoarchaeological series (Pieve di Pava, Italy) [128] that seems to be in conflict with modern disease prevalence data as TBM is a very rare extra-pulmonary form, occurring in less than 1% of all clinically active TB cases today [124,129-130]. Genchi & co-workers [128] state that their hypothesis is supported by 1) the short duration of TBM that is not long enough to allow bony changes (e.g., GIs) to develop on the endocranial surface and 2) the presence of cases with GIs not only among TB patients but also non-TB individuals from documented skeletal collections (Hamann-Todd Human Osteological Collection [94] and Robert J. Terry Anatomical Skeletal Collection [96,99]).*”.

We respectfully disagree with your statement that *“this paper does not offer any evidence to support that proposal or for discussing the evidence offered by other authors, as the paper published by Genchi & colleagues does.”*. In the original version of our manuscript (lines 381–429), we already addressed and refuted the three aforementioned arguments proposed by Genchi & co-workers (2025) regarding the invalidity of the earlier interpretation that endocranial granular impressions are pathognomonic features of clinically active tuberculosis disease (specifically tuberculous meningitis).

While we considered these three counterarguments sufficient to demonstrate the weaknesses of Genchi & colleagues’ (2025) hypothesis and to support the validity of the earlier interpretation, five additional, more detailed counterarguments were presented in the *S1 Text*. In that file, we also provided an extended discussion of the three counterarguments already summarised in the main text. Moreover, in several of these counterarguments (particularly in points 5–8 of the *S1 Text*), we cited and critically analysed other key studies addressing the tuberculosis-specific nature of endocranial granular impressions (e.g., Hershkovitz & co-workers, 2002; Spekker & colleagues, 2020), including how the examined groups were created, how reliable the recorded causes of death were, and how frequent the cases with endocranial granular impressions were in the examined groups. These analyses further clarify why, in contrast to Genchi & co-workers’ (2025) hypothesis, we continue to support the earlier interpretation that endocranial granular impressions are signs of clinically active tuberculosis disease (specifically tuberculous meningitis) rather than only tuberculosis infection.

Taking into account not only your but also the other reviewers’ valuable comments on this section, we now realise that including a shortened version of the five additional counterarguments in the main text would improve the clarity and completeness of our reasoning. We have therefore integrated the additional points – originally presented only in the *S1 Text* – into the revised *Discussion* section.

“*Genchi & colleagues [136] calculated only the frequency of cases with GIs (and based on this, the frequency of TBM), not the prevalence of TBM, in the examined osteoarchaeological series from mediaeval Pieve di Pava. Then, they compared the estimated frequency of TBM to modern disease prevalence data. However, only the prevalence of TBM in mediaeval Pieve di Pava may be appropriate to be compared to modern disease prevalence data. As it was highlighted by Milner & Boldsen [139], estimating the prevalence of a disease (e.g., TBM) in osteoarchaeological series is no easy task, and is much more complicated than considering only the frequency of a lesion type (e.g., GIs) or the number of diagnosed cases (e.g., number of diagnosed TB(M) cases) in the examined sample;*” (lines 447–455 in the revised version of our manuscript; point 1 in the refutation of Genchi & co-workers’ (2025) hypothesis);

“*Only disease prevalence data deriving from a representative sample may be appropriate to be used for comparing to modern disease prevalence data but at the current state of research, it is not possible to properly estimate the prevalence of TBM in the once-lived population of mediaeval Pieve di Pava: a) the sample examined by Genchi & co-workers [136] does not seem to be representative of the once-lived population of mediaeval Pieve di Pava (e.g., children under the age of 2 years are missing; and the cemetery is associated with a religious building) [139] and b) although there are some promising palaeoepidemiological studies [e.g., 112] that try to assess the prevalence of TB disease in osteoarchaeological series, further research is needed to develop better methods;*” (lines 456–464 in the revised version of our manuscript; point 2 in the refutation of Genchi & co-workers’ (2025) hypothesis);

“*The Hamann-Todd Human Osteological Collection and the Robert J. Terry Anatomical Skeletal Collection were established when and where many people, possibly everyone, were exposed to TB bacilli [102,112,158]; thus, individuals from the above-mentioned documented skeletal collections are likely to had TB infection – in the TB group, they were not only infected by TB but the infection progressed into clinically active TB disease in all cases, whereas in the non-TB group, they had at least latent TB infection that may have progressed into clinically active TB disease in an unknown number of cases. Based on the above, if Genchi & co-workers’ [136] hypothesis would be correct and GIs would be signs of TB infection rather than clinically active TB(M) disease, the frequency of cases with GIs in the non-TB groups should be very similar to their frequency in the TB groups. However, as it has been presented by Spekker & colleagues [104], in the Robert J. Terry Anatomical Skeletal Collection, the frequency of cases with GIs was about 10 times higher in the TB group than in the non-TB group. This difference between the two groups was statistically extremely significant, which confirms that GIs are signs of clinically active TB(M) disease rather than TB infection;*” (lines 514–528 in the revised version of our manuscript; point 6 in the refutation of Genchi & co-workers’ (2025) hypothesis);

“*If Genchi & co-workers’ [136] hypothesis would be correct and it would be the meningeal tubercles developing at the beginning of the TB infection rather than the meningeal tubercles forming during the pathogenesis of TBM that exert pressure on the adjacent endocranial surface for a long time (not weeks but more likely years or decades); and thus, lead to the development of GIs, the frequency of cases with GIs should be much higher not only in the non-TB groups of the Hamann-Todd Human Osteological Collection and Robert J. Terry Anatomical Skeletal Collection, where the majority of the individuals (if not all of them) are likely to had at least latent TB infection, but also in the TB groups, where all individuals are unquestionably to had not only TB infection but clinically active TB disease. However, as it has been presented by Spekker & colleagues [104], even in the TB group of the Robert J. Terry Anatomical Skeletal Collection, GIs were present in less than one-third of the examined cases; and*” (lines 529–540 in the revised version of our manuscript; point 7 in the refutation of Genchi & co-workers’ (2025) hypothesis);

“*The localisation pattern of GIs on the endocranial surface is not random as we would expect it if Genchi & co-workers’ [136] hypothesis would be correct and it would be the meningeal tubercles forming at the beginning of the TB infection after haematogenous dissemination of TB bacilli into the central nervous system that exert pressure on the inner skull surface for a long time; and thus, result in the development of GIs. As it has been presented by Schultz [99], Schultz & Schmidt-Schultz [103], and Spekker & colleagues [104], instead of being randomly scattered on the endocranial surface, GIs are localised on the skull base and lower lateral skull vault. During the pathogenesis of TBM, the formation of meningeal tubercles presents a very specific pattern (first the disease involves the meninges of the basal areas but in the end, it extends to the meninges covering the cerebral convexities). The localisation pattern of GIs on the endocranial surface follows this specific pattern of tubercle development during the pathogenesis of TBM (see Figure 1 in [104]).*” (lines 541–552 in the revised version of our manuscript; point 8 in the refutation of Genchi & co-workers’ (2025) hypothesis).

We hope that these clarifications and additions will make our reasoning clearer and demonstrate why the refutation of Genchi & colleagues’ (2025) hypothesis is necessary and directly relevant to the accurate diagnosis of MMG3-75.

b) We respectfully disagree with your statement that insufficient space was dedicated to the differential diagnosis. In the original version of our manuscript (lines 436–510), we provided a detailed discussion of the potential aetiologies (chordoma, chondrosarcoma, bone metastases, and pyogenic or fungal osteomyelitides) that should be considered in the differential diagnosis of the observed clival lesions. We would like to note that **Reviewer 4** expressed a very positive opinion regarding our differential diagnosis section, identifying it as one of the strengths of the manuscript (“*The strength of this paper is in listed and explained differential diagnoses…*”). **Reviewer 2** also gave a favourable evaluation, describing the section as “*thorough, scientifically accurate, and provides further assurance for the results*”.

Nonetheless, to further strengthen the scientific justification of our diagnosis (i.e., tuberculous clival osteomyelitis as the primary diagnosis in MMG3-75), we have expanded the description of the aetiologies considered alongside tuberculous clival osteomyelitis in the differential diagnosis, with particular emphasis on the macromorphological characteristics of the osteolytic lesions:

“*… in TB osteomyelitis, the osteolytic lesions are typically well-defined, with minimal or no marginal sclerosis and little or no reactive new bone formation in the surrounding bone, while sequestration is relatively uncommon [70-73].*” (tuberculous clival osteomyelitis; lines 114–116 in the revised version of our manuscript);

“*… which cause extensive lytic bone destruction without evidence of surrounding sclerosis (i.e., well-defined or ill-defined osteolytic lobulated lesions without sclerotic margins) [54-55,58,159,165,167,170-172].*” (clival chordoma; lines 580–582 in the revised version of our manuscript);

“*… which cause lytic bone destruction (i.e., ill-defined osteolytic lesions) [159,171-172,177-178,180,186,189].*” (clival chondrosarcoma; lines 611–612 in the revised version of our manuscript);

“*… the disease can be classified as 1) (predominantly) osteolytic, 2) (predominantly) osteoblastic, or 3) mixed [172,195,197,199,201-202]; consequently, it exhibits highly variable morphological patterns and radiological appearances [172].*” (clival bone metastasis; lines 645–647 in the revised version of our manuscript); and

“*In pyogenic osteomyelitis, osteolytic lesions are typically ill-defined, often with thick sclerotic margins; reactive new bone formation in the surrounding bone and sequestrum are common [71-73,239-240]. Fungal osteomyelitis can show similar features (e.g., ill-defined osteolytic lesions or marginal sclerosis), but they are less consistent and may be absent [240-241].*” (pyogenic and fungal clival osteomyelitis; lines 690–694 in the revised version of our manuscript).

Finally, we regret but it is unclear to us why it would be necessary to include a discussion in our manuscript about whether the observed clival lesions could or could not serve as palaeopathological diagnostic criteria for tuberculous meningitis. These bony changes most likely developed as a consequence of tuberculous osteomyelitis of the clivus, not tuberculous meningitis. Throughout the original (and also the revised) version of our manuscript, we took great care to clearly distinguish between the two forms of the disease concurrently affecting MMG3-75: the clival lesions were attributed to tuberculous clival osteomyelitis, while the endocranial granular impressions and abnormal blood vessel impressions were considered signs of tuberculous meningitis.

**REFERENCES:**

Genchi ML, Giuffra V, Campana S, Riccomi G. 2025. Are endocranial granular impressions pathognomonic of tuberculous meningitis or a marker of tuberculous infection? An investigation on a medieval osteoarcheological assemblage from Italy. *International Journal of Paleopathology* **49**: 81–92. DOI: 10.1016/j.ijpp.2025.03.002

Hershkovitz I, Greenwald CM, Latimer B, Jellema LM, Wish-Baratz S, Eshed V, Dutour O, Rothschild BM. 2002. *Serpens endocrania symmetrica* (SES): A new term and a possible clue for identifying intrathoracic disease in skeletal populations. *American Journal of Physical Anthropology* **118**(3): 201–216. DOI: 10.1002/ajpa.10077

Schultz M. The role of tuberculosis in infancy and childhood in prehistoric and historic populations. In: Pálfi G, Dutour O, Deák J, Hutás I, editors. *Tuberculosis: Past and present*. Szeged, Hungary: TB Foundation & Budapest, Hungary: Golden Book Publisher; 1999. pp. 503–507.

Schultz M. 2001. Paleohistopathology of bone: A new approach to the study of ancient diseases. *American Journal of Physical Anthropology* **116**(Suppl. 33): 106–147. DOI: 10.1002/ajpa.10024

Schultz M. Light microscopic analysis in skeletal paleopathology. In: Ortner DJ, editor. *Identification of pathological conditions in human skeletal remains*. San Diego, CA, USA: Academic Press; 2003. pp. 73–107. DOI: 10.1016/B978-012528628-2/50043-0

Schultz M, Schmidt-Schultz TH. 2015. Is it possible to diagnose TB in ancient bone using microscopy?. *Tuberculosis* **95**(Suppl. 1): S80–S86. DOI: 10.1016/j.tube.2015.02.035

Spekker O, Hunt DR, Paja L, Molnár E, Pálfi G, Schultz M. 2020. Tracking down the White Plague: The skeletal evidence of tuberculous meningitis in the Robert J. Terry Anatomical Skeletal Collection. *PLOS ONE* **15**(3): e0230418. DOI: 10.1371/journal.pone.0230418

Spekker O, Hunt DR, Berthon W, Molnár E, Pálfi G. 2022. Insights into the diagnostic efficacy and macroscopic appearance of endocranial bony changes indicative of tuberculous meningitis: Three example cases from the Robert J. Terry Anatomical Skeletal Collection. *International Journal of Osteoarchaeology* **32**(2): 444–456. DOI: 10.1002/oa.3079

23) **Reviewer 3** mentioned that “*In consequence, in my opinion, the discussion is long and disorganized and does not offer a well-sustained answer about the possible relationship of lesions in the clivus and TB. The differential diagnosis at the end of the paper rejects some possible explanations for other causes of these lesions, arguing that they are very rare, but also that the clivus lesions in TB are. Therefore, the evidence offered is not quite convincing.*”.
a) Thank you for your thorough and constructive comments on the *Discussion* section. We regret if the discussion appeared lengthy or insufficiently structured. The complexity of the presented case, especially the detailed consideration of pathogenesis and differential diagnosis as well as the refutation of Genchi & colleagues’ (2025) hypothesis, necessitated a comprehensive approach that we consider indispensable for establishing tuberculosis as the primary diagnosis in MMG3-75. Therefore, we were unable to shorten these parts without compromising scientific rigor.

Nevertheless, to facilitate the reader’s understanding and to support the logical flow of our argument, we divided the *Discussion* section into subsections – with separate units dedicated to pathogenesis (*Pathogenesis of TB in MMG3-75* subsection), the refutation of Genchi & co-workers’ (2025) hypothesis (*Corroborative evidence for TB in MMG3-75* subsection), and the differential diagnosis of the clival lesions (*Differential diagnosis of the clival lesions in MMG3-75* subsection). Furthermore, at the beginning of each aforementioned subsection, we inserted brief introductory sentences explaining their focus and rationale, as well as transitional statements to guide the reader smoothly through the logical progression of our discussion.

“*Assuming our hypothesis (i.e., MMG3-75 suffered from TB at the time of death, and that this disease caused the observed cranial lesions) is correct, two plausible pathogenetic scenarios can be proposed: (1) TB first affected the clivus (i.e., TB clival osteomyelitis) and subsequently extended to the meninges (i.e., TBM) (S2 Figure), or (2) it involved the meninges first (i.e., TBM) and later reached the clivus (i.e., TB clival osteomyelitis) (S3 Figure).*” (lines 364–369 in the revised version of our manuscript; *Pathogenesis of TB in MMG3-75* subsection);

“*Unfortunately, aDNA analyses did not detect Mycobacterium tuberculosis DNA in the two hard tissue samples (a tooth and a skull fragment) taken from MMG3-75. Consequently, the only corroborative (macromorphological) evidence supporting a diagnosis of TB(M) in this younger adult male comes from the presence of GIs in multiple locations on the inner skull surface.*” (lines 423–427 in the revised version of our manuscript; *Corroborative evidence for TB in MMG3-75* subsection);

“*Given that in MMG3-75 the palaeopathological diagnosis of TBM was established based on the presence of GIs in multiple locations on the inner skull surface, and considering that these lesions constitute the only corroborative evidence indicating that this younger adult male suffered from TB at the time of death, it is inevitable to critically examine Genchi & colleagues’ above arguments [136]*.” (lines 440–444 in the revised version of our manuscript; *Corroborative evidence for TB in MMG3-75* subsection); and

“*Although the overall nature and co-occurrence of the clival bony changes observed in MMG3-75 (mild cortical erosion and multiple, well-circumscribed osteolytic lesions) suggest that TB (specifically TB clival osteomyelitis) could plausibly have been the underlying cause, and the presence of endocranial GIs in multiple locations on the endocranial surface of MMG3-75 provides corroborative evidence that this younger adult male suffered from TB(M) at the time of death, we cannot rule out the possibility that the clival lesions were associated with another pathological condition that was concurrently present with TB in MMG3-75. This consideration makes a detailed differential diagnosis of the clival bony changes indispensable. The most relevant infectious and non-infectious aetiologies that should be considered include clival malignancies (e.g., chordoma, chondrosarcoma or metastases), pyogenic osteomyelitis (e.g., caused by Pseudomonas or Staphylococcus spp.), and fungal osteomyelitis (e.g., caused by Aspergillus or Mucor spp.) [24,26,50,53,68-69].*” (lines 561–572 in the revised version of our manuscript; *Differential diagnosis of the clival lesions in MMG3-75* subsection).

We hope that this subdivision and added clarity will help the reader follow our reasoning more easily and that, despite the length, the revised *Discussion* section will be perceived as better structured.

b) We also regret that you found the differential diagnosis insufficiently convincing. A similar concern was also raised by **Reviewer 1**, who noted that “*Again, they exclude other conditions based on their clinical rarity, but the TB clivus osteomyelitis is extremely rare as well.*”. However, we would like to clarify again that the exclusion of other possible aetiologies considered alongside tuberculous clival osteomyelitis (chordoma, chondrosarcoma, bone metastases, and pyogenic or fungal osteomyelitides) during the detailed differential diagnosis was not based on the rarity of these pathological conditions. We are fully aware that tuberculous clival osteomyelitis is itself an extremely rare pathological condition, which is precisely why we found it important to report this case. In line with another of your valuable suggestions, we have elaborated in the *Conclusions* section – which was separated from the *Discussion* based on your recommendation – on the scientific relevance and significance of our case (lines 828–857 in the revised version of our manuscript):

“*Our study contributes to expanding knowledge and understanding of the manifestations and pathogenesis of TB in past human populations by presenting and discussing in detail a unique case of probable TB involvement of the clivus in MMG3-75. To the best of our knowledge, MMG3-75 represents the first reported archaeological case of TB clival osteomyelitis with associated meningitis. It provides an exceptional insight into an extremely rare form of TB, the geographical, temporal, and social distribution of which is still unknown from both prehistoric and historic times, including in mediaeval Hungary from where MMG3-75 derives. Furthermore, MMG3-75 not only serves as a reference and aid for the identification and interpretation of similar cases in other human osteoarchaeological series but also offers an invaluable perspective on the disease experience of past individuals who, like this younger adult male, may have suffered from TB clival osteomyelitis.*

*Although once thought to have been conquered, TB still poses a tremendous public health threat today – largely due to the global emergence and spread of antibiotic-resistant strains. This underlines the urgent need to better understand the disease, including its history, in line with the notion that “the past informs the present”. Widespread use of antibiotics in the treatment of TB caused certain forms of the disease to vanish or become extremely rare, yet we must now consider the possibility that such pre-antibiotic-era manifestations may reappear or occur more frequently in the future. This is particularly concerning because these disease forms are now almost entirely unfamiliar to clinicians, complicating timely diagnosis and initiation of appropriate treatment, which can negatively impact clinical outcomes in modern cases resembling pre-antibiotic-era manifestations of TB. Although we currently have no information on the prevalence of TB clival osteomyelitis in the past, contemporary knowledge of this disease form is also extremely limited, as the number of reported cases does not exceed two dozen to date. Therefore, beyond its palaeopathological significance, MMG3-75 also holds relevance for modern medical practice, providing comparative data that may aid in the diagnosis and clinical management of similar modern TB cases, thereby contributing to improved outcomes in such instances. More broadly, our research highlights how archaeological case studies can enhance modern medical knowledge and understanding, helping to bridge the gap between past and present manifestations of TB – one of the world’s oldest and still most lethal human infectious diseases.*”.

As we already highlighted above, **Reviewer 4** expressed a very positive opinion regarding our differential diagnosis section, identifying it as one of the strengths of the manuscript (“*The strength of this paper is in listed and explained differential diagnoses…*”). **Reviewer 2** also gave a favourable evaluation, describing the section (lines 436–510 in the original version of our manuscript) as “*thorough, scientifically accurate, and providing further assurance for the results*”.

Nevertheless, **Reviewer 2** suggested that adding clearer emphasis on the similarities and differences between our case (MMG3-75) and alternative diagnoses would improve the reader’s understanding. Following this suggestion, at the end of the discussion of each potential aetiology (chordoma, chondrosarcoma, bone metastases, and pyogenic or fungal osteomyelitides), we have included explicit comparisons with our case, listing the key characteristics based on which we excluded or considered these pathological conditions less likely to underlie the observed bony changes:

“*The biological sex (male) and estimated age at death (30–39 years) of MMG3-75 do not argue against the diagnosis of clival chordoma, as these malignant bone tumours show no sex predilection and most commonly affect individuals belonging to the 20–40-year age group. In contrast, the osteolytic lesions in MMG3-75, although also lacking surrounding sclerosis as typically seen in this tumour type, display a markedly different spatial pattern. They are neither situated along the midline nor confined to the spheno-occipital synchondrosis but are instead scattered across the clivus of this younger adult male in an apparently random manner (Figs 3–4 and 8–9). Furthermore, the three larger osteolytic lesions identified within the clivus of MMG3-75 are clearly separated from one another and show no evidence of interconnection, thus lacking the lobulated appearance that would be expected in a clival chordoma (Fig 9). Taken together, the combination of the spatial distribution pattern and non-lobulated morphology of the osteolytic lesions observed in this younger adult male is difficult to reconcile with a clival chordoma, which typically arises from the spheno-occipital synchondrosis and presents as a centrally located (midline), well-circumscribed, expansile, lobulated soft-tissue mass. Based on these observations, clival chordoma can be ruled out with high certainty as a diagnostic option in MMG3-75;*” (lines 585–600 in the revised version of our manuscript);

“*Although the biological sex (male) and estimated age at death (30–39 years) of MMG3-75 do not rule out the possibility of a skull-base (clival) chondrosarcoma, he falls slightly below the typical age range (middle-aged adults) for this tumour type. Furthermore, several morphological features, particularly the spatial distribution of the osteolytic lesions in the clivus of this younger adult male, argue against this diagnosis. The well-circumscribed lesions are neither laterally located (off-midline) nor confined to the petro-clival synchondroses, but are instead randomly scattered across the clivus of MMG3-75 (Figs 3–4 and 8–9), with the three larger osteolytic lesions being spatially separate and showing no signs of interconnection. Although a larger, non-perforating osteolytic lesion is present within the clivus of this younger adult male on each side, adjacent to the petro-clival synchondroses, a skull-base (clival) chondrosarcoma would be expected to arise in the synchondral cartilage and progressively erode the surrounding bone, compromising the cortical layers and producing perforating lesions. In contrast, CBCT images (Fig 8) reveal no evidence of erosion in the bones adjacent to either petro-clival synchondrosis (neither in the laterally adjacent petrous apex nor in the medially adjacent clivus), providing strong evidence that these osteolytic lesions did not develop from the synchondral cartilage inward into the surrounding bone. The observed spatial distribution of the osteolytic lesions detected in MMG3-75, together with the intact cortical bone adjacent to the petro-clival synchondroses and the lack of the characteristic ring-and-arc calcification pattern on the CBCT images (Fig 8), is difficult to reconcile with a skull-base (clival) chondrosarcoma, which typically presents as a laterally located (off-midline), well-circumscribed, expansile, lobulated soft-tissue mass with a ring-and-arc pattern of chondroid calcification, mostly arising from the petro-clival synchondrosis. Taken together, these observations strongly suggest that a skull-base (clival) chondrosarcoma is unlikely to account for the mild cortical erosion and multiple, well-circumscribed osteolytic lesions observed in MMG3-75;*” (lines 614–638 in the revised version of our manuscript);

“*Although the biological sex (male) of MMG3-75 is consistent with the slight male predominance reported for clival bone metastases, his estimated age at death (30–39 years) is younger than that of the older individuals typically affected by this pathological condition. Furthermore, beyond the clivus, no similar osteolytic lesions were identified elsewhere in the skull or postcranial skeleton of this younger adult male, whereas secondary bone malignancies usually present as multiple alterations, most often involving different parts of the axial skeleton. Finally, all clival changes in MMG3-75 are purely lytic, with no evidence of new bone formation. However, purely osteolytic or osteoblastic metastatic lesions are exceedingly rare; in most cases, both osteoclastic resorption and osteoblastic new bone formation are present to some extent, resulting in mixed alterations. Considering the relatively young estimated age at death of MMG3-75, the purely lytic nature of the clival lesions and their restriction to the clivus (with no similar alterations detected elsewhere in the axial skeleton), bone metastasis can be regarded as a less likely diagnosis in this younger adult male;*” (lines 656–669 in the revised version of our manuscript); and

“*The estimated age at death (30–39 years) of MMG3-75 is not in favour of the diagnosis of pyogenic or fungal skull-base (clival) osteomyelitides, as he is considerably younger than the elderly individuals typically affected by these pathological conditions. Moreover, these osteomyelitides usually arise secondary to ear or paranasal sinus infections, but CBCT images (Fig 8) show that both the middle and inner ears of MMG3-75 were intact, and the posterior wall of the sphenoid sinus – which became visible due to post-mortem damage – was also unaffected (Fig 4b). Although marginal sclerosis, reactive new bone formation, and sequestrum are frequently observed at the osteolytic lesions in such osteomyelitides, especially in pyogenic cases, none of these features were detected in this younger adult male, as demonstrated by the CBCT images (Fig 8). Considering the relatively young estimated age at death of MMG3-75, the absence of any evidence for preceding ear (i.e., otitis media or otitis interna) or paranasal sinus infection (i.e., sphenoid sinusitis), and the lack of marginal sclerosis, reactive new bone formation or sequestrum, it is highly improbable that a pyogenic or fungal skull-base (clival) osteomyelitis was responsible for the development of the mild cortical erosion and multiple, well-circumscribed osteolytic lesions observed in this younger adult male.*” (lines 695–710 in the revised version of our manuscript).

We hope these additions meet your expectations, and provide a clearer and more convincing argument.

**REFERENCE:**

Genchi ML, Giuffra V, Campana S, Riccomi G. 2025. Are endocranial granular impressions pathognomonic of tuberculous meningitis or a marker of tuberculous infection? An investigation on a medieval osteoarcheological assemblage from Italy. *International Journal of Paleopathology* **49**: 81–92. DOI: 10.1016/j.ijpp.2025.03.002

24) **Reviewer 3** suggested that “*I suggest also including a separate section of CONCLUSIONS, where the authors also include a list of possible limitations and the relevance of their study. I hope all these suggestions will be helpful to the authors to improve their research.*”.

Thank you very much for your valuable suggestion regarding the structure of our manuscript. In accordance with your recommendation, we have separated the *Discussion* and *Conclusions* sections. In the newly created *Conclusions* section, we have included a detailed summary of our study’s limitations, as well as its scientific relevance and contributions (lines 713–857 in the revised version of our manuscript):

“*In summary, the younger adult male (MMG3-75) from the 16^th^-century-CE mass grave No. 3 of the Mohács National Memorial Site (Sátorhely, southwestern Hungary) represents a unique case of OATB with regard to the localisation and nature of the observed skeletal lesions. This uniqueness stems from two major aspects:*

(1) *Based on the macromorphological characteristics and co-occurrence of the clival bony changes (mild cortical erosion and multiple, well-circumscribed osteolytic lesions), and after careful consideration of the possible differential diagnoses, the alterations are most consistent with TB clival osteomyelitis. The clivus is an extremely rare site of involvement in TB, with fewer than two dozen of cases reported to date [21,23-24,26-28,50,53,59,61-68]; and*
(2) *Based on the presence of GIs (and ABVIs) in multiple locations on the endocranial surface, MMG3-75 also suffered from TBM. In the existing modern medical literature, TB clival osteomyelitis associated with meningitis has been mentioned in only a few cases [21,27-28,53,65,67].*

*Nevertheless, several limitations should be considered when interpreting our findings:*

(1) *Although the pathological bony changes observed in the skull remains of MMG3-75 correspond to several diagnostic criteria for TB compiled from the palaeopathological literature (summarised in Table 1), indicating that this younger adult male may have suffered from TB with concomitant involvement of the clivus (i.e., TB clival osteomyelitis) and the meninges (i.e., TBM), it was necessary to employ a three-step analytical approach to substantiate this hypothesis.*

*First, to demonstrate that the formation of the cranial lesions detected in MMG3-75 is plausible in the context of TB, we had to reconstruct the possible pathogenetic scenarios that could account for their development. Two pathways were proposed: (1) TB first affected the clivus (i.e., TB clival osteomyelitis) and subsequently extended to the meninges (i.e., TBM) (S2 Figure), or (2) it involved the meninges first (i.e., TBM) and then reached the clivus (i.e., TB clival osteomyelitis) (S3 Figure). It should be noted that although we were able to outline these potential pathogenetic scenarios, illustrating how the cranial bony changes could have developed if our hypothesis is correct (i.e., if TB was indeed the underlying cause in MMG3-75), unfortunately, we could not clarify which of these scenarios took place in MMG3-75, nor could we establish the primary focus of the TB infection within this younger adult male’s body (from where the TB bacilli presumably first reached either the clivus (S2 Figure) or the meninges (S3 Figure) via haematogenous spread).*

*Second, to reinforce that TB could indeed plausibly have been the underlying cause of the cranial lesions observed in MMG3-75, it was necessary to obtain corroborative evidence that this younger adult male suffered from the disease at the time of death. The presence of GIs in multiple locations on the inner surface of the skull of MMG3-75 provides such (macromorphological) evidence supporting a diagnosis of TB(M), which may also underlie the formation of the detected endocranial ABVIs. The observation of clival bony changes, potentially consistent with TB clival osteomyelitis, suggests that TB may have contributed not only to the development of the GIs and ABVIs but also to these lesions, and that this younger adult male may have been affected by both disease forms (i.e., TBM and TB clival osteomyelitis) simultaneously. We outlined two plausible pathogenetic scenarios explaining how the TB infection might have spread between the clivus and the meninges (either from the clivus to the meninges or vice versa), and how, in each case, the detected cranial alterations could have formed, with the GIs and ABVIs resulting from TBM and the clival bony changes from TB clival osteomyelitis (S2 Figure and S3 Figure). Nonetheless, a direct causal link between TB and the clival lesions cannot be established, as the presence of GIs in MMG3-75 provides (macromorphological) evidence only for TBM, which cannot account for the clival bony changes, and not for concurrent TB clival osteomyelitis, which could explain these alterations. Therefore, while TB clival osteomyelitis is indeed a plausible underlying cause of the observed clival lesions, we cannot conclusively assert that it occurred alongside TBM in this younger adult male, nor can we exclude the possibility that another pathological condition concomitantly present with TB in MMG3-75 contributed to the formation of these alterations.*

*Third, to substantiate that TB clival osteomyelitis was indeed the most likely primary diagnosis underlying the bony changes detected in the clivus of MMG3-75, we had to conduct a detailed differential diagnosis. During this process, both non-infectious (chordoma, chondrosarcoma, and bone metastases) and infectious (pyogenic and fungal osteomyelitides) aetiologies were considered. Taking into account the macromorphological characteristics of the alterations, together with the individual’s age at death and sex, we were able to either exclude or consider the aforementioned pathological conditions as less likely to explain the clival lesions observed in MMG3-75. Consequently, TB clival osteomyelitis is regarded as the most plausible – and therefore primary – diagnosis in the case of this younger adult male. Unfortunately, it still cannot be ruled out with absolute certainty that another pathological condition, concurrently presenting with TB in MMG3-75, contributed to the development of these bony changes, as neither the individual lesions nor their co-occurrence can be regarded as pathognomonic of TB (specifically TB clival osteomyelitis);*

(2) *The only corroborative (macromorphological) evidence supporting a diagnosis of TB(M) in MMG3-75 comes from the presence of GIs in multiple locations on the inner skull surface, since these endocranial alterations have been described as specific signs of TB(M) in the palaeopathological literature. Unfortunately, attempts to obtain complementary (molecular) evidence for this morphology-based diagnosis were unsuccessful, as the aDNS analysis failed to detect remnants of Mycobacterium tuberculosis DNA in the two hard tissue samples (the upper right lateral incisor and a fragment of the left parietal bone) taken from MMG3-75.*

*It is important to emphasise that, although aDNA-based investigations are highly valuable in complementing the findings of traditional morphology-based examinations, they should not be considered as exclusive and indispensable tools for confirming a diagnosis of TB in skeletal human remains. The successful detection of M. tuberculosis DNA in prehistoric and historic TB cases depends on multiple factors. In MMG3-75, for instance, the aDNA analysis revealed an overall poor preservation of endogenous DNA in both samples, which may account for the negative result. Another possible explanation is that, to avoid irreversible damage to the clival bony changes through invasive sampling and to preserve these morphologically unique and diagnostically significant alterations for potential future investigations, no samples were taken directly from the affected bone area or its immediate surroundings, where the likelihood of detecting M. tuberculosis aDNA would presumably have been higher.*

*Although the endogenous human DNA content recovered from the two hard tissue samples in MMG3-75 was less than 0.01%, indicating* *generally poor biomolecular preservation in this younger adult male, this does not necessarily preclude the potential success of alternative molecular diagnostic approaches, such as lipid biomarker analyses. Lipid biomarker-based investigations could provide valuable additional evidence to support the morphology-based diagnosis of TB in skeletal human remains – particularly in cases where endogenous DNA preservation is poor, as in MMG3-75. However, like aDNA analyses, these methods also require invasive sampling, preferably from the lesion-affected bone or areas adjacent to it. Targeted sampling of these bone regions would be crucial to demonstrate a direct causal link between the clival alterations and any detected remnants of Mycobacterium tuberculosis DNA, and hence between these bony changes and TB itself. Nonetheless, because of the unique nature of the clival lesions observed in MMG3-75, we believe that preservation currently outweighs the potential benefits of further invasive molecular investigations. We remain committed, however, to exploring these approaches in the future, once the necessary resources and conditions (e.g., less-destructive sampling techniques) become available; and*

(3) *Finally, it should also be noted that, at present, the contextual interpretation of the case of MMG3-75 remains limited, as anthropological analyses, including palaeopathological investigations, have so far been completed for only a subset of the individuals buried in mass grave No. 3 of the Mohács National Memorial Site, with the majority of skeletons still awaiting evaluation. Based on preliminary results from the already examined skeletons, several individuals appear to exhibit GIs and other endocranial alterations consistent with TBM. These findings suggest that MMG3-75 may not have been the only individual affected by TB among those interred in the burial pit. However, to determine the frequency of the disease, and to draw further conclusions from these observations, a comprehensive assessment of the entire skeletal assemblage will be required. Consequently, a more detailed contextualisation of the case of MMG3-75 will only be possible upon completion of the ongoing analyses.*

*Our study contributes to expanding knowledge and understanding of the manifestations and pathogenesis of TB in past human populations by presenting and discussing in detail a unique case of probable TB involvement of the clivus in MMG3-75. To the best of our knowledge, MMG3-75 represents the first reported archaeological case of TB clival osteomyelitis with associated meningitis. It provides an exceptional insight into an extremely rare form of TB, the geographical, temporal, and social distribution of which is still unknown from both prehistoric and historic times, including in mediaeval Hungary, from where MMG3-75 derives. Furthermore, MMG3-75 not only serves as a reference and aid for the identification and interpretation of similar cases in other human osteoarchaeological series but also offers an invaluable perspective on the disease experience of past individuals who, like this younger adult male, may have suffered from TB clival osteomyelitis.*

*Although once thought to have been conquered, TB still poses a tremendous public health threat today – largely due to the global emergence and spread of antibiotic-resistant strains. This underlines the urgent need to better understand the disease, including its history, in line with the notion that “the past informs the present”. Widespread use of antibiotics in the treatment of TB caused certain forms of the disease to vanish or become extremely rare, yet we must now consider the possibility that such pre-antibiotic-era manifestations may reappear or occur more frequently in the future. This is particularly concerning because these disease forms are now almost entirely unfamiliar to clinicians, complicating timely diagnosis and initiation of appropriate treatment, which can negatively impact clinical outcomes in modern cases resembling pre-antibiotic-era manifestations of TB. Although we currently have no information on the prevalence of TB clival osteomyelitis in the past, contemporary knowledge of this disease form is also extremely limited, as the number of reported cases does not exceed two dozen to date. Therefore, beyond its palaeopathological significance, MMG3-75 also holds relevance for modern medical practice, providing comparative data that may aid in the diagnosis and clinical management of similar modern TB cases, thereby contributing to improved outcomes in such instances. More broadly, our research highlights how archaeological case studies can enhance modern medical knowledge and understanding, helping to bridge the gap between past and present manifestations of TB – one of the world’s oldest and still most lethal human infectious diseases.*”.

We believe these additions have enhanced the clarity of our manuscript. We greatly appreciate your constructive feedback, which has helped us to improve the quality of our work.

25) **Reviewer 4** commented that “*The manuscript The first archaeological case of tuberculous clival osteomyelitis with associated meningitis from a 16th-century-CE mass grave from Mohács (southwestern Hungary) is worth to be published in PLOS ONE. The overall impression is that this study is written in detail and comprehensively. The authors used all known and acknowledged diagnostic criteria for tuberculous clival osteomyelitis and tuberculous meningitis, additionally illustrated with pictures and detailed explanations. The strength of this paper is in listed and explained differential diagnoses, in consulted clinical studies on the basis of which it was possible to reconstruct the first case of tuberculous clival osteomyelitis from archaeological context but also in impressive reference list. Special weight and importance of the work is given by Detailed refutation of Genchi & colleagues’ (2025) hypothesis that endocranial granular impressions are signs of tuberculosis infection rather than clinically active tuberculosis disease in Supporting Information where authors in eight points strengthen the previous hypothesis that GIs are pathognomonic features of TB(M) (rather than TB infection).
    This is a useful work and I believe that it can add to the body of literature on the subject of tuberculosis in the ancient populations in general but more specifically in manifestations of tuberculosis of the skull. There are some minor suggestions that can be added to the manuscript, but it is up to authors to accept it or not because they don’t have an extra relevance to the topic of this paper.*”.

We would like to express our sincere gratitude for your thorough and thoughtful review of our manuscript. We truly appreciate your kind words and positive assessment of our work and are delighted that you consider our contribution a useful addition to the growing body of literature on tuberculosis in the past.

Your acknowledgement of the detailed differential diagnoses and the integration of relevant clinical studies is greatly appreciated. We are especially thankful for your recognition of our efforts to critically engage with and refute the hypothesis proposed by Genchi & co-workers (2025) regarding endocranial granular impressions.

**REFERENCE:**

Genchi ML, Giuffra V, Campana S, Riccomi G. 2025. Are endocranial granular impressions pathognomonic of tuberculous meningitis or a marker of tuberculous infection? An investigation on a medieval osteoarcheological assemblage from Italy. *International Journal of Paleopathology* **49**: 81–92. DOI: 10.1016/j.ijpp.2025.03.002

26) **Reviewer 4** noted that “*In one part of the manuscript was written that “anthropological analysis was carried out on the fairly preserved and almost complete skeleton” and in the other that “It should be noted that the taphonomic damage, as well as the missing elements of the skeleton of MMG3-75 significantly hampered the macromorphological observations”. Maybe it would be useful to provide an overview of which parts of the skeleton have been preserved, either descriptively or schematically*.”.

We would like to thank you for this insightful comment regarding the state-of-preservation of the skeleton of MMG3-75. As also noted in response to **Reviewer 3**, we fully agree that providing a clearer overview of the extant skeletal elements is essential for the accurate interpretation of the macromorphological observations. In the revised version of our manuscript, we have therefore expanded the relevant part of the *Methods* section with a more detailed description of the state-of-preservation of the skeleton of MMG3-75 to explain why it hampered the palaeopathological evaluation. The revised part reads as follows (lines 199–208 in the revised version of our manuscript):

“*Although the overall state-of-preservation of the skeleton of MMG3-75 was fairly good and almost complete, the taphonomic damage and the missing elements – particularly in skeletal regions regarded as predilection sites for TB-related lesions (e.g., vertebrae; ribs; large, weight-bearing joints; and the inner surface of the skull) significantly hampered the macromorphological observations. For instance, while most of the vertebrae and ribs were present, the latter, in particular, were extremely fragmentary and incomplete. The skull was considerably fragmentary, with some missing parts and several post-mortem damaged areas on the endocranial surface. Some of the large, weight-bearing joints also showed clear post-mortem damage.*”.

To further assist the reader, we have also included a photograph of the skeleton laid out in anatomical order (now ***Figure 2***), which complements the textual description and provides a transparent overview of the skeleton’s state-of-preservation.

“***Figure 2:*** *State-of-preservation of the skeleton of MMG3-75 (photo by Marcos De Andrés Montero, Luca Kis, and Olga Spekker)*.”

We hope that these additions address your concern and enhance the clarity and interpretability of the skeletal evidence presented in our study.

27) **Reviewer 4** commented that “*Since the skeleton of MMG3-75 derives from a mass grave that contained approximately 320 individuals who were the victims of the Battle of Mohács it would be interesting to mention (although that is not the topic of this paper) whether this male exhibited perimortem trauma*.”.
    a) We thank you for this insightful comment. As we have also noted in response to the **Academic Editor**'s comment, the exact identity of the individuals buried in mass grave No. 3 of the Mohács National Memorial Site remains uncertain at this stage of research. While the burial pit is associated with the aftermath of the Battle of Mohács (1526), any interpretation regarding who these individuals were is still hypothetical. At the current stage of research, it is therefore not possible to determine whether MMG3-75 was a soldier who fought in the battle, a member of the Hungarian camp or a civilian from a nearby settlement, etc. Likewise, the exact circumstances of the death of MMG3-75 remain uncertain – it is unclear whether they perished in the battle, died while retreating or were executed afterwards, etc.

To clarify this point, we have inserted an additional sentence in the revised version of our manuscript immediately following the relevant part of the *Material* section, which reads as follows (lines 150–156 in the revised version of our manuscript):

“*It is important to note that, to date, several hypotheses have been proposed regarding the identity of the individuals buried in the five known mass graves at the Mohács National Memorial Site, suggesting that they may have been soldiers, members of the Hungarian camp or civilians from nearby settlements [77,79]. Ongoing research seeks to reassess these hypotheses and to shed further light on both the identities of these individuals and the circumstances of their death – whether they perished in the battle, died while retreating or were executed afterwards.*”.

We hope this clarification resolves any ambiguity regarding the context of mass grave No. 3 and the identity of MMG3-75.

b) We fully agree with you that the presence or absence of *perimortem* trauma on the skeletal remains of MMG3-75 is not the focus of our study, which is why such evidence was not discussed in the manuscript. Nonetheless, we would like to note that several *perimortem* injuries were indeed identified on the skeleton of MMG3-75: signs of non-lethal *perimortem* trauma were observed on the distal third of the diaphysis of the left humerus and on the proximal epiphysis of the left radius. These injuries exhibit the typical morphology of cut marks and align perfectly when the elbow is flexed, suggesting that they likely resulted from a single traumatic event. While a possible interpretation of the observed *perimortem* traumas is that they were defensive in nature, several hypotheses can still be proposed regarding the circumstances under which such defensive behaviour occurred (e.g., during close-range combat, while retreating, in the course of an execution, or in other violent encounters). That said, even the presence of such *perimortem* traumas do not allow us to draw firm conclusions about whether MMG3-75 was a soldier who fought in the battle, a member of the Hungarian camp or a civilian from a nearby settlement, etc.

Within the framework of the *Mohács 500 project*, a dedicated subproject is focused on the study of *perimortem* trauma observed on the skeletal remains of individuals buried in mass grave No. 3 of the Mohács National Memorial Site. Analyses are currently ongoing and are expected to provide a more comprehensive understanding of the circumstances surrounding their deaths in the future.

28) **Reviewer 4** mentioned that “*It would also be interesting to state whether other individuals with signs of tuberculosis were found during the analysis of other skeletons from the mass grave.*”.

We thank you for this important observation. Within the Mohács 500 *project*, another subproject is specifically focused on investigating the occurrence of infectious diseases (e.g., tuberculosis and syphilis) among the individuals buried in mass grave No. 3. These analyses are currently still in progress.

Preliminarily, we can report that several of the skeletons examined so far have shown granular impressions and other endocranial alterations indicative of tuberculous meningitis. Based on these findings, it seems that, in addition to MMG3-75, other individuals from mass grave No. 3 also have suffered from tuberculosis. However, as the research is ongoing, we are not yet able to provide definitive frequency data or a detailed discussion of these observations.

In accordance with your suggestion, we have inserted a paragraph into the relevant part of the *Conclusions* section of the manuscript (limitations) to indicate that the analysis of all individuals buried in mass grave No. 3 of the Mohács National Memorial Site is still in progress. We also note there that, among those already examined, several exhibit endocranial alterations consistent with tuberculous meningitis, suggesting that MMG3-75 may not have been the only individual affected by the disease, but a full assessment of the entire skeletal assemblage is required to determine the frequency of tuberculosis and the significance of these findings, which will only be possible upon completion of the ongoing analyses (lines 816–827 in the revised version of our manuscript):

“*Finally, it should also be noted that, at present, the contextual interpretation of the case of MMG3-75 remains limited, as anthropological analyses, including palaeopathological investigations, have so far been completed for only a subset of the individuals buried in mass grave No. 3 of the Mohács National Memorial Site, with the majority of skeletons still awaiting evaluation. Based on preliminary results from the already examined skeletons, several individuals appear to exhibit GIs and other endocranial alterations consistent with TBM. These findings suggest that MMG3-75 may not have been the only individual affected by TB among those interred in the burial pit. However, to determine the frequency of the disease, and to draw further conclusions from these observations, a comprehensive assessment of the entire skeletal assemblage will be required. Consequently, a more detailed contextualisation of the case of MMG3-75 will only be possible upon completion of the ongoing analyses.*”.

In the revised version of our manuscript, we tried to execute all suggestions of the reviewers. We received rather contrasting opinions from them regarding certain aspects (e.g., the differential diagnosis of the clival lesions and the refutation of Genchi & colleagues’ (2025) hypothesis). Nonetheless, we aimed to find balanced, compromise solutions whenever possible, and, when this was not feasible, we provided detailed justifications for our choices. We hope this new version of our manuscript will be suitable for publication in PLOS ONE.

Thank you again for the reviewers’ insightful and constructive comments and your editorial work!

Yours sincerely,

Dr. Olga Spekker, PhD

corresponding author

Department of Biological Anthropology, University of Szeged

Közép fasor 52, H-6726, Szeged, Hungary

Email: [olga.spekker@gmail.com](mailto:olga.spekker@gmail.com)

Tel: +36 20 807 72 94
